# C6-Alkoxy substituted penicillins are potent non-covalently binding inhibitors of the SARS-CoV-2 main protease

**DOI:** 10.1039/d5md00789e

**Published:** 2025-10-27

**Authors:** Dorian-Gabriel Muntean, Wojtek Treyde, Linda Kinena, Eidarus Salah, Hani Choudhry, Fernanda Duarte, Lennart Brewitz, Christopher J. Schofield

**Affiliations:** a Chemistry Research Laboratory and the Ineos Oxford Institute for Antimicrobial Research, University of Oxford 12 Mansfield Road OX1 3TA Oxford UK lennart.brewitz@chem.ox.ac.uk christopher.schofield@chem.ox.ac.uk; b Latvian Institute of Organic Synthesis Aizkraukles 21 LV-1006 Riga Latvia; c Department of Biochemistry, Center for Artificial Intelligence in Precision Medicines, King Abdulaziz University Jeddah The Kingdom of Saudi Arabia

## Abstract

Inhibition of the SARS-CoV-2 main protease (M^pro^) by small-molecules is a validated strategy for COVID-19 treatment. There is a need for improved M^pro^ inhibitors, including because M^pro^ mutations can confer resistance to clinically used M^pro^ inhibitors. Previous work has revealed the potential of penicillin derivatives as covalently reacting M^pro^ inhibitors. Here we report studies on M^pro^ inhibition by C6-alkoxy substituted penicillin derivatives. The combined mass spectrometric and computational evidence imply most of the tested penicillin C6-alkoxy derivatives bind *via* non-covalent interactions at the M^pro^ active site, resulting in potent substrate-competitive inhibition. Some penicillin C6-alkoxy derivatives ((*R*)-, but not (*S*)-sulfoxides) manifest covalent reaction to different extents. Penicillins and related drugs are widely used antibiotics, acting *via* covalent reaction of their β-lactam with a nucleophilic serine in their transpeptidase targets to give an acyl–enzyme complex. The results imply penicillin derivatives can be developed to inhibit enzymes via mechanisms other than formation of stable acyl–enzyme complexes.

## Abstract 2

Introduction of a C6-alkoxy group on the penicillin scaffold can alter the mechanism of SARS-CoV-2 main protease (M^pro^) inhibition.

## Graphical abstract

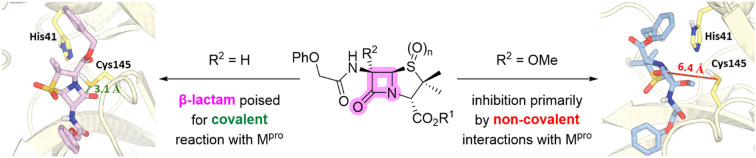

## Introduction

**Fig. 1:**
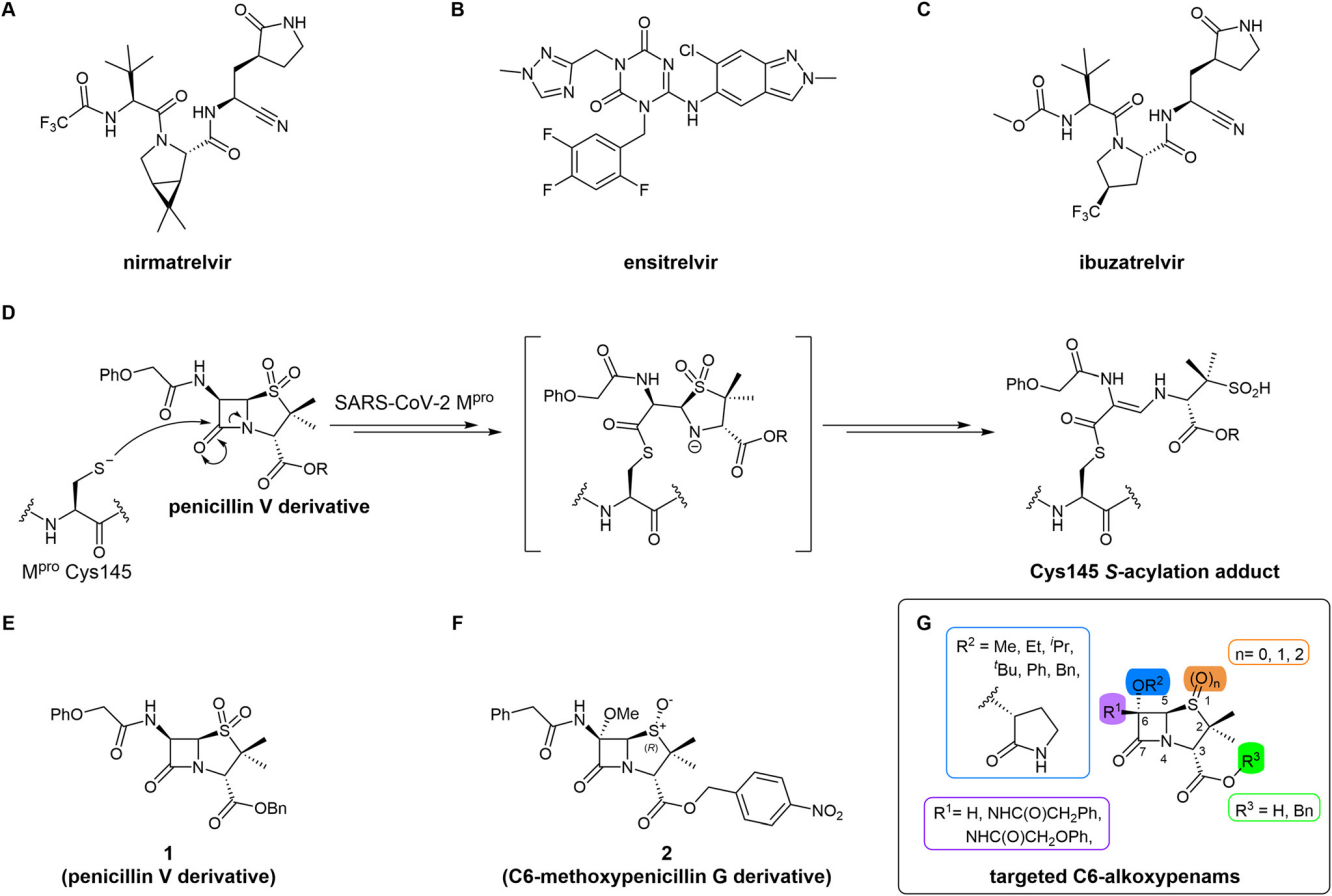
SARS-CoV-2 M^pro^ inhibitors. (A) Nirmatrelvir;^6^ (B) ensitrelvir;^9^ and (C) ibuzatrelvir.^28^ (D) Outline mechanism for covalent modification of M^pro^ Cys145 by penicillin V sulfone esters showing a crystallographically observed adduct.^40,41^ (E) The penicillin V sulfone derivative 1.^40,41^ (F) The C6-methoxy penicillin G (*R*)-sulfoxide derivative 2.^40^ (G) The penicillin C6-alkoxy derivatives investigated for M^pro^ inhibition in this work.

Small-molecule inhibitors of the severe acute respiratory syndrome coronavirus-2 (SARS-CoV-2) main protease (M^pro^) have helped to overcome the coronavirus disease 2019 (COVID-19) pandemic.^1–3^ M^pro^ is a nucleophilic cysteine protease that catalyses hydrolysis of the viral polyproteins 1a and 1ab (pp1a/1ab) to give non-structural proteins; inhibiting M^pro^ catalysis disrupts viral replication.^2–5^ Nirmatrelvir^6^ (Fig. 1A) was the first M^pro^ inhibitor to be widely used in the clinic – it inhibits M^pro^ through reversible covalent reaction of its nitrile group with the nucleophilic cysteine (Cys145). Several derivatives of nirmatrelvir are also clinically used,^7,8^ but to date only one non-covalently binding M^pro^ inhibitor, ensitrelvir^9^ (Fig. 1B), has been developed for clinical use. Multiple other M^pro^ inhibitor scaffolds have also been investigated,^2,3,10,11^ including for inhibiting by irreversible covalent reaction,^12–16^ and tight, non-covalent binding to M^pro^.^11,17–24^

There is continued interest in the development of improved M^pro^ inhibitors^25–30^ in part because evidence suggests that variations in the M^pro^ active site can confer resistance against clinically used M^pro^ inhibitors.^31–35^ Furthermore, the clinical scope of nirmatrelvir is limited, as it requires co-administration with ritonavir in order to limit metabolic degradation.^6^ The second-generation investigational M^pro^ inhibitor ibuzatrelvir (PF-07817883; Fig. 1C) has improved metabolic stability compared to nirmatrelvir and does not require co-administration with ritonavir.^28,29^ Other factors to consider in the development of improved M^pro^ inhibitors that may be used on a large scale in a future pandemic include the cost of production (in part to facilitate global use), the need for sustainable synthesis, and environmental impacts.

Since their clinical introduction, penicillins and subsequent generations of β-lactam antibiotics became and, despite the emergence of widespread resistance, continue to be the most extensively used class of antibacterials.^36,37^ Their effectiveness, proven safety profiles, affordable manufacturing,^38^ and rapid environmental degradation due to the reactive nature of the β-lactam ring,^39^ foster their continued use. Considering the distinctive reactivity profile of the β-lactam drugs from an antimicrobial perspective, we developed a mass spectrometry (MS)-based screen for M^pro^ inhibitors at the onset of the COVID-19 pandemic, investigating β-lactams and related antimicrobial compounds.^40–43^

MS and crystallographic analyses have shown that M^pro^ Cys145 covalently reacts with the β-lactam group of some penicillin V derivatives (*e.g.*, 1; Fig. 1D and E) to give stable acyl–enzyme complexes resulting in M^pro^ inhibition.^40,41^ By contrast, the results of these MS studies also suggested that a penicillin G derivative with a methoxy substituent at the C6 position (2, Fig. 1F) apparently inhibited *via* non-covalent binding to M^pro^.^40^ This was an interesting observation, as it is known that the introduction of a C6-methoxy group onto the penicillin scaffold or of a C7-methoxy group onto the cephalosporin scaffold can alter the stability and conformations of the acyl–enzyme adducts formed when reacting with their transpeptidase targets and nucleophilic serine β-lactamases (Fig. S1).^44–46^

Here we report a structure–activity relationship (SAR) analysis and computational studies on C6-alkoxy penicillins, which provide insights into their mechanism of SARS-CoV-2 M^pro^ inhibition. The results imply that most C6-alkoxy penicillins inhibit M^pro^ through non-covalent binding at the active site, suggesting that this inhibition mode should also be considered when investigating the effects of related β-lactams on other nucleophilic enzymes beyond M^pro^.

## Results

### Computational studies suggest stable non-covalent binding of C6-methoxy penicillins to the M^pro^ active site

**Fig. 2:**
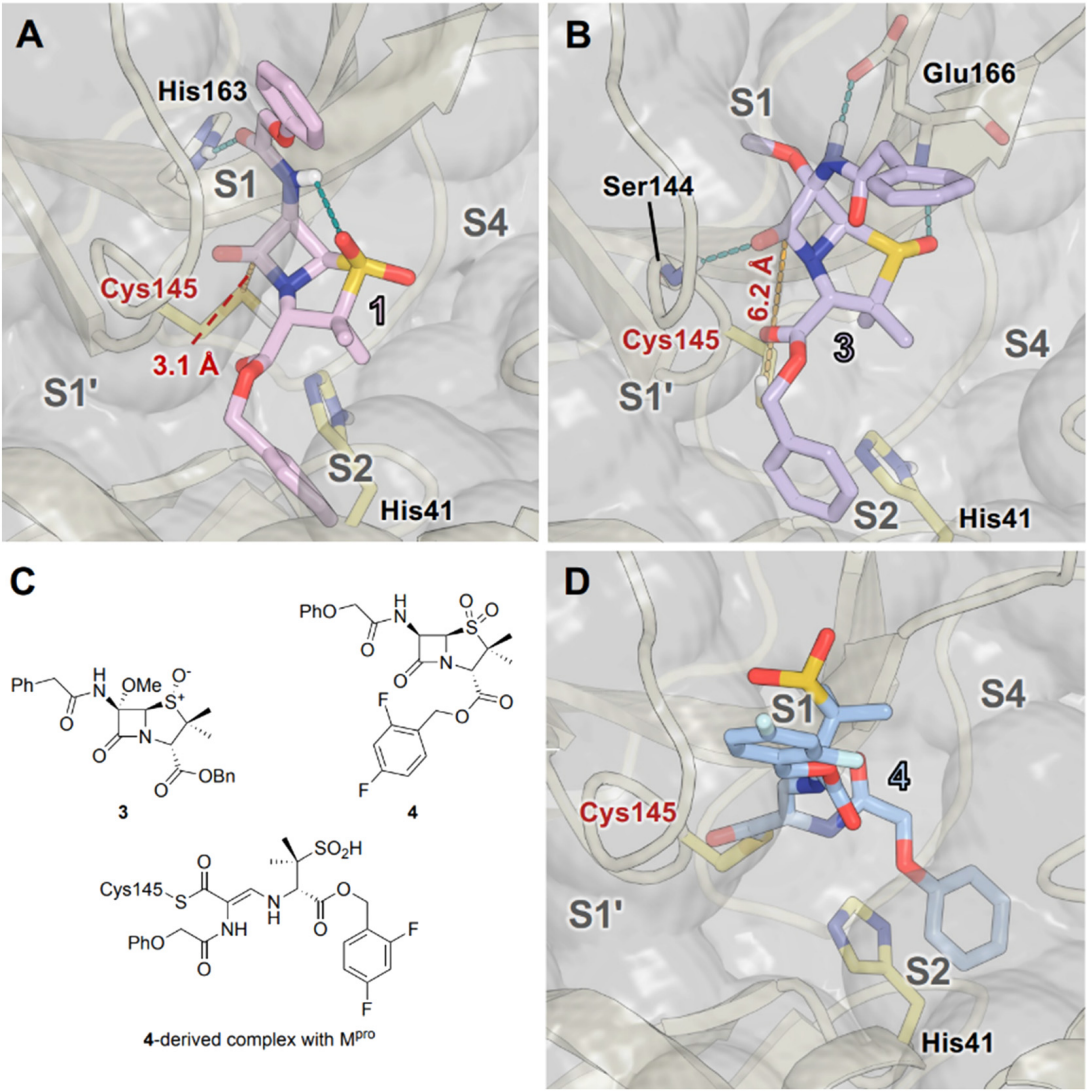
Modelling studies predict the penicillin C6-methoxy group may impair covalent reaction with M^pro^. (A and B) Representative structures of (A) M^pro^ : 1 and (B) M^pro^ : 3 complexes from cluster analysis of 3 × 100 ns MD simulations. The C6-amido and C3-ester groups of 1 occupy the M^pro^ S1 and S2 pockets, respectively. 1 is predicted to interact with His163 and to form an intramolecular hydrogen bond involving its C6 amide NH and the pro-*R* sulfone O. The C6-methoxy group of 3 is predicted to bind in the S1 pocket; 3 is also predicted to interact with Ser144 and Glu166. (C) Penicillin G derivative 3, penicillin V derivative 4, and the structure of 4 following covalent reaction with M^pro^. (D) Crystal structure view of the complex formed following covalent reaction of 4 with M^pro^ (PDB ID: 7Z59).^41^ The S(Cys145)–C(O)(β-lactam) distances for these representative snapshots are in red (dashed yellow lines), with average values from MD simulations provided in Fig. S5. Atoms are coloured by element (N: blue, O: red, S: yellow, F: cyan, H: white), with C-atoms coloured differently for each penicillin derivative. Active site His41 and Cys145 side chain C-atoms are in bright yellow; C-atoms of other residues are in pale yellow.

To explore how C6-alkoxy substitutions may influence penicillin binding at the M^pro^ active site, we initially conducted docking studies and molecular dynamics (MD) simulations. Specifically, we compared the binding of penicillin V sulfone benzyl ester 1 with the C6-methoxy penicillin G (*R*)-sulfoxide derivative 3, which is the benzyl ester analogue of the initial hit 2 (Fig. 2C). This comparison maintains consistency in terms of C3 substitution, as previous structure–activity relationship studies indicate that C3 modifications have minimal impact on inhibition potency.^42^

The MD simulations predict a distinct binding mode for 1 within the M^pro^ active site. The C6-phenoxyacetamido substituent of 1 is positioned in the S1 pocket, and the benzyl ester group of 1 occupies the S2 pocket (Fig. 2A). This binding mode differs from that of nirmatrelvir, for which crystal structures show that it interacts with the S1, S2, S3 and S4 pockets (Fig. S2A). It also differs from the crystallographically observed structure of the covalently bound product of 4 (Fig. 2D), a derivative of 1, where the C6-phenoxyacetamido substituent binds in the S2 pocket (Fig. S2B). Despite these differences, the MD simulations indicate that 1 is well positioned for covalent reaction with Cys145 (Fig. 2A), as evidenced by a S(Cys145)–C(O)(β-lactam) distance of 4.8 Å ± 1.5 Å and a Bürgi–Dunitz angle of 97° ± 24° (mean ± standard deviation (SD) across three concatenated independent replicates of 100 ns MD simulations; Fig. S5A), consistent with MS evidence.^40,41^ The simulations also show hydrogen bond interactions between the C6 amido NH group of 1 and the pro-*S* sulfone oxygen, potentially promoting a conformation favourable for M^pro^ binding.

Modelling of 3, which contains a C6-methoxy group, suggests a binding mode similar to 1, with the C6-methoxy group occupying the S1 pocket (Fig. 2B and S3). While additional hydrogen interactions with Ser144 and Glu166 are observed with 3, the interaction with His163 is lost and the average S(Cys145)–C(O)(β-lactam) distance increases to 6.9 Å ± 1.7 Å, suggesting a binding mode that is likely not productive for covalent reaction (Fig. S5B). Overall, the modelling results suggest that C6-alkoxy substitution may hinder proximity of Cys145 to the β-lactam ring in a manner that is not suitable for nucleophilic attack by Cys145, but may enable stable, non-covalent binding involving hydrogen bonding interactions.

### C6-Methoxy penicillin V derivatives inhibit M^pro^*via* non-covalent binding

**Fig. 3:**
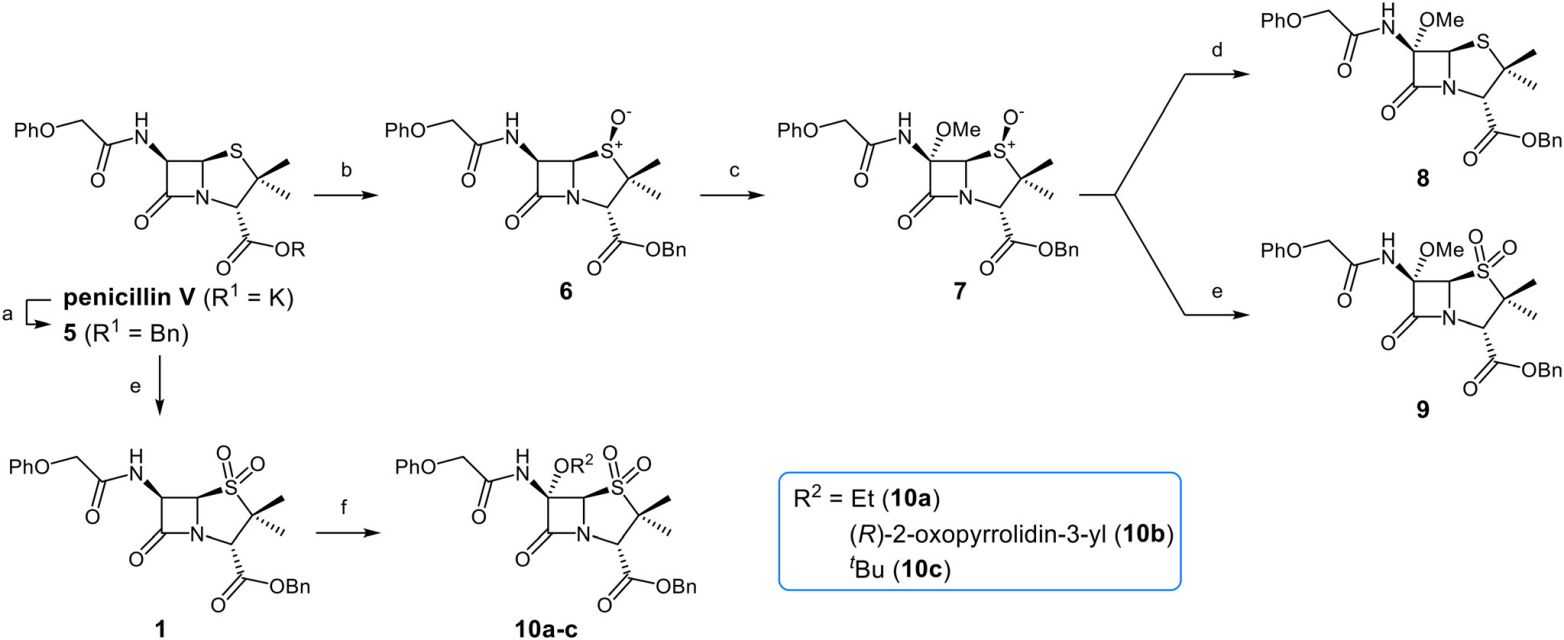
Synthesis of C6-methoxypenicillin V derivatives. Reagents and conditions: a) BnBr (1.1 equiv.), *N*,*N*-dimethylformamide, rt, 18 h, 82%; b) *m*CPBA (1.1 equiv.), CH_2_Cl_2_, 0 °C to rt, 4 h, 72%; c) ^*t*^BuOCl (1.5 equiv.), LiOMe solution in MeOH (1 M; 1.1 equiv.), CH_2_Cl_2_, −30 °C, 1 h, 54%; d) KI (18 equiv.), AcCl (7 equiv.), *N*,*N*-dimethylformamide, 0 °C, 1 h, 99%; e) *m*CPBA (2–2.5 equiv.), CH_2_Cl_2_, rt, 16 h, 39–65%; f) ^*t*^BuOCl (1.5 equiv.), base (EtONa or Et_3_N; 1.1 equiv.), ROH (EtOH or (*R*)-3-hydroxypyrrolidin-2-one; 6–10 equiv.), CH_2_Cl_2_, − 30 °C, 1 h, 7–35%.

Building on these results, a set of penicillin V C6-methoxy derivatives were synthesised in 3–4 steps from penicillin V to investigate whether the presence of a C6-methoxy group affects the mechanism of M^pro^ inhibition as predicted by the modelling studies (Fig. 3). Thus, penicillin V was alkylated using benzyl bromide to give the reported ester 5,^41^ which was oxidized using *meta*-chloroperbenzoic acid (*m*CPBA) to give the corresponding (*S*)-sulfoxide 6 or sulfone 1, depending on the stoichiometry of the reaction. Conversion of the (*S*)-sulfoxide 6 to the C6-methoxy derivative 7 was achieved in 45% yield employing a variation of a reported protocol that used *tert*-butyl hypochlorite (^*t*^BuOCl) and LiOMe solution in methanol (1 M) at −30 °C.^47^ The (*S*)-sulfoxide 7 was efficiently reduced to the thioether 8 using KI/AcCl (99% yield)^48^ or oxidized to the sulfone 9 using *m*CPBA (65% yield).

The effects of the C6-methoxy penicillin V derivatives 7–9 on M^pro^ catalysis were investigated using reported solid phase extraction coupled to mass spectrometry (SPE-MS) assays,^40^ and compared to those of the corresponding penicillin V derivatives lacking the C6-methoxy substituent (Table 1). Analysis of the half-maximal inhibitory concentrations (IC_50_ values) reveals that increasing the thiazolidine sulfur oxidation state of the penicillin V derivatives from the sulfide 5*via* the (*S*)-sulfoxide 6 to the sulfone 1 correlated with increased inhibition potency (Table 1, entries i–iii), in accord with reported results.^41^ Note that the IC_50_ for 6 differs from that reported, an observation which may reflect differences in assay conditions.^12,40^

**Table 1:** Effects of penicillin V derivatives on SARS-CoV-2 M^pro^ catalysis

	Penicillin derivative	IC_50_^a^ [μM]		Penicillin derivative	IC_50_^a^ [μM]
i	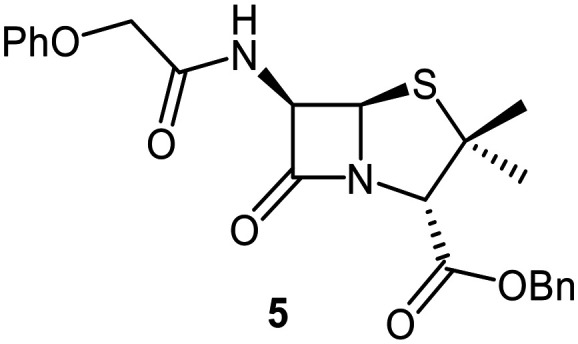	>50 (reported:^41^ >50)	vi	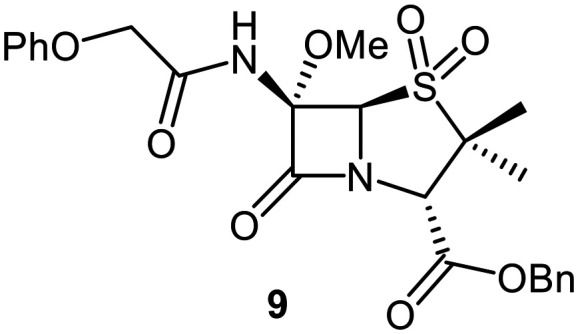	>50
ii	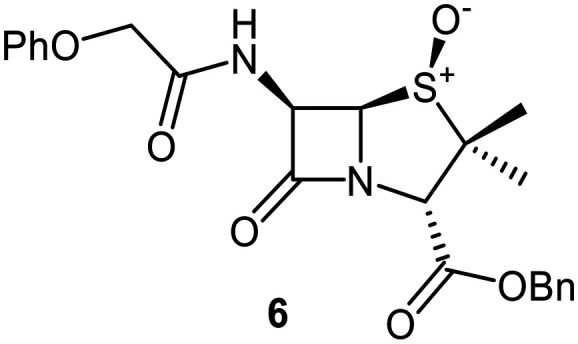	6.3 ± 1.0 (reported:^41^ ∼23)	vii	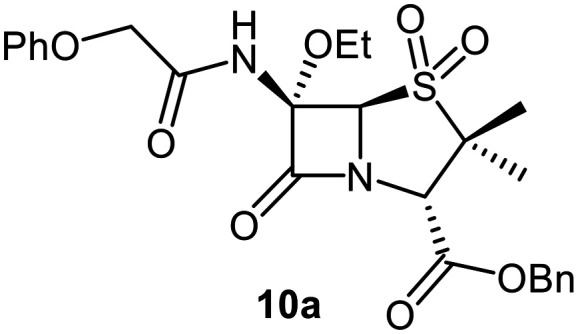	5.8 ± 1.9
iii	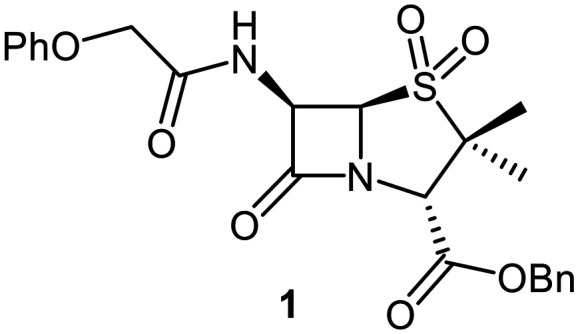	2.9 ± 0.4 (reported:^41^ ∼6.6)	viii	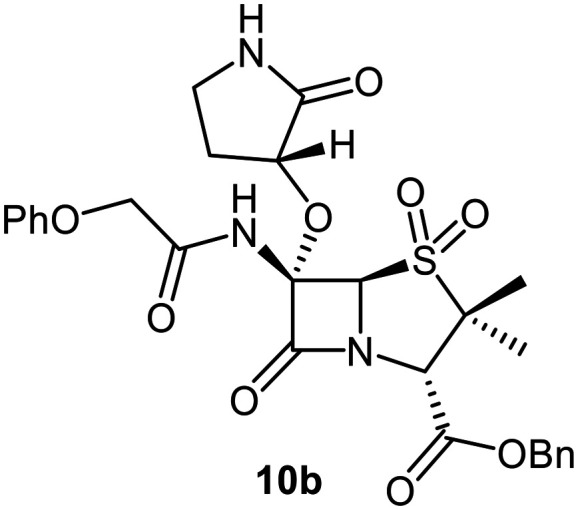	5.3 ± 1.6
iv	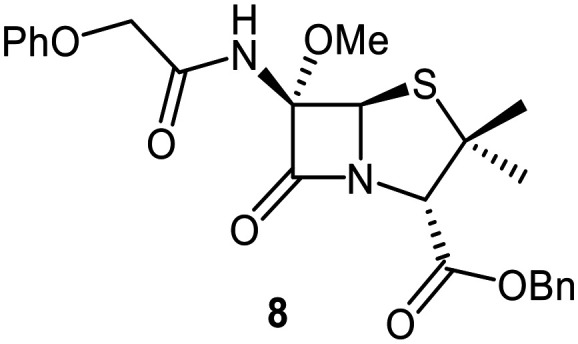	>50	ix	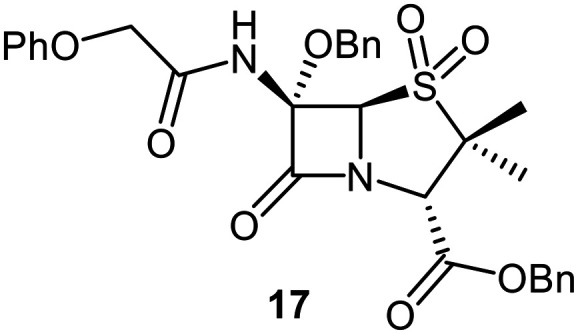	8.3 ± 2.6
v	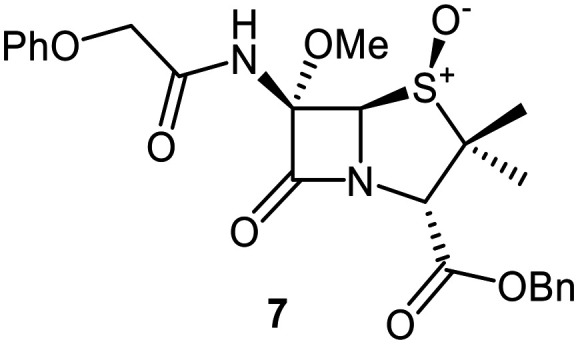	2.3 ± 0.3	x	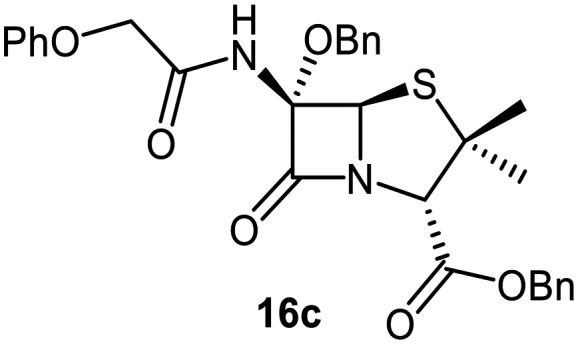	>50

a M^pro^ inhibition assays were performed using SPE-MS as reported,^12,40^ utilizing SARS-CoV-2 M^pro^ (0.05 μM) and substrate (2.0 μM). Results are means of three independent experiments (*n* = 3; mean ± SD), each in technical duplicates. Representative dose–response curves are shown in Fig. S8. Bn: –CH_2_Ph.

C6-Methoxy substitution substantially affected the inhibition profiles of the penicillin V derivatives (Table 1, entries iv–vi): addition of a C6-methoxy substituent to the penicillin V sulfone benzyl ester 1 ablated M^pro^ inhibition within the tested concentration range (9; IC_50_ > 50 μM; Table 1, entry vi), whereas its addition to the corresponding (*S*)-sulfoxide 6 did not alter potency within experimental error (7; IC_50_ ∼ 2.3 μM; Table 1, entry v). Note that variable IC_50_ values were observed for the C6-methoxy penicillin V derivative 8, the C6-methoxy analogue of 5. Like 5, 8 showed no inhibition in the tested concentration range in independent triplicates using freshly prepared DMSO stock solutions. However, additional replicates using premade DMSO stock solutions indicated moderate inhibition by 8 (IC_50_ ∼ 5.0 μM), perhaps reflecting the limited stability of 8 in DMSO, with partial oxidation of 8 to the corresponding more potent (*S*)-sulfoxide inhibitor 7, possibly accounting for the observed discrepancies. This proposal is in accord with observations that penicillin derivatives degrade gradually in DMSO, potentially compromising reproducibility. Alternatively, the mode of inhibition (see below) may influence reproducibility for penicillin derivatives with relatively low inhibitory potency (IC_50_ > 10 μM).

**Fig. 4:**
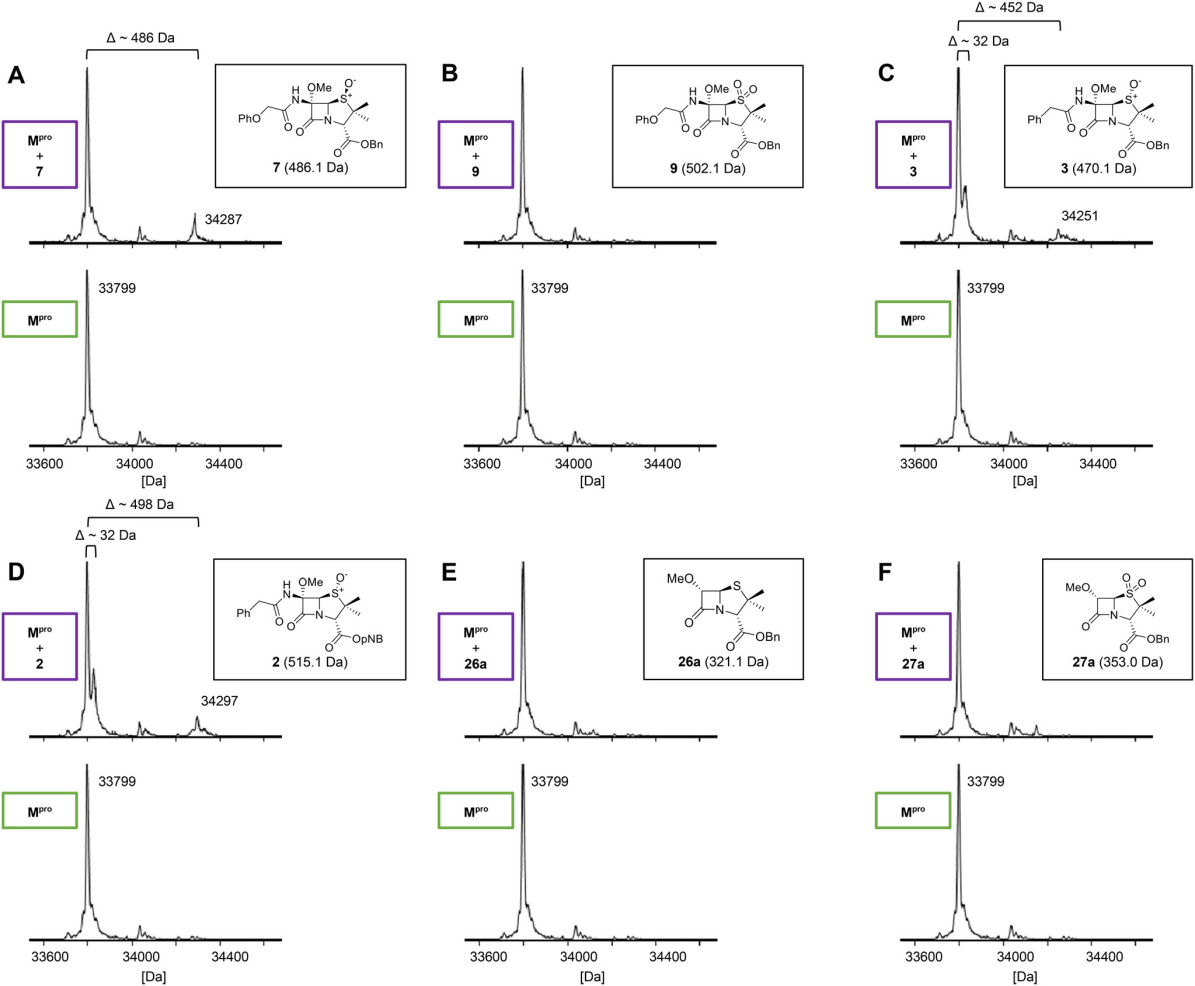
Reaction of C6-methoxy penams with isolated recombinant M^pro^. Analysis of M^pro^ reactions with C6-methoxy penam derivatives: (A) 7, (B) 9, (C) 3, (D) 2, (E) 26a, (F) 27a prior (bottom) and after >15 h of incubation (top) with the respective β-lactam. Assays were performed using SPE-MS as reported,^40^ employing SARS-CoV-2 M^pro^ (3.0 μM) and, if appropriate, a β-lactam (15 μM) in buffer (20 mM HEPES, pH 7.5). Representative spectra of technical duplicates are shown. Protein-observed mass spectra for other tested β-lactams are in Fig. S9. Note that in no case were substantial amounts of species corresponding to covalent modification observed, suggesting inhibition by these compounds is (at least, principally) *via* non-covalent binding; in the cases of 2 and 3 low levels of species with mass shifts corresponding to potential acyl–enzyme complexes were observed.

Protein-observed MS studies were performed to test for covalent reaction of the penicillin V derivatives with M^pro^ under denaturing conditions.^40^ The penicillin V derivatives were incubated with M^pro^ (5 : 1 ratio) for >15 h prior to analysis by MS (Fig. 4). The results reveal that, in accord with the predicted binding modes of β-lactams 1 and 3 (Fig. 2) and reported results on their reactivity with SARS-CoV-2 M^pro^,^40,41^ the introduction of a C6-methoxy substituent onto the penicillin V scaffold prevents, at least, efficient covalent reaction with M^pro^ (Fig. 4A and B).

### The nature of the penam C6-alkoxy substituent affects inhibition potency

C6-Alkoxy derivatives of penicillin V were synthesised to investigate the effects of C6 substituents other than methoxy groups on M^pro^ inhibition potency and mechanism. The synthesis of the C6-ethoxy penicillin V sulfone derivative 10a was achieved from 1 in 35% yield by substituting LiOMe/MeOH for NaOEt/EtOH (Fig. 3). Notably, this route simplifies reported syntheses of 10a that employed more steps and/or undesirable, potentially toxic reagents.^49,50^ Similarly, the secondary alcohol (*R*)-3-hydroxypyrrolidin-2-one was employed to synthesize 10b in 6% yield from 1 under slightly modified conditions, with triethylamine as the base; the γ-lactam group was introduced to possibly mimic the interactions of the nirmatrelvir γ-lactam group with the M^pro^ S1 pocket.^6^

**Fig. 5:**
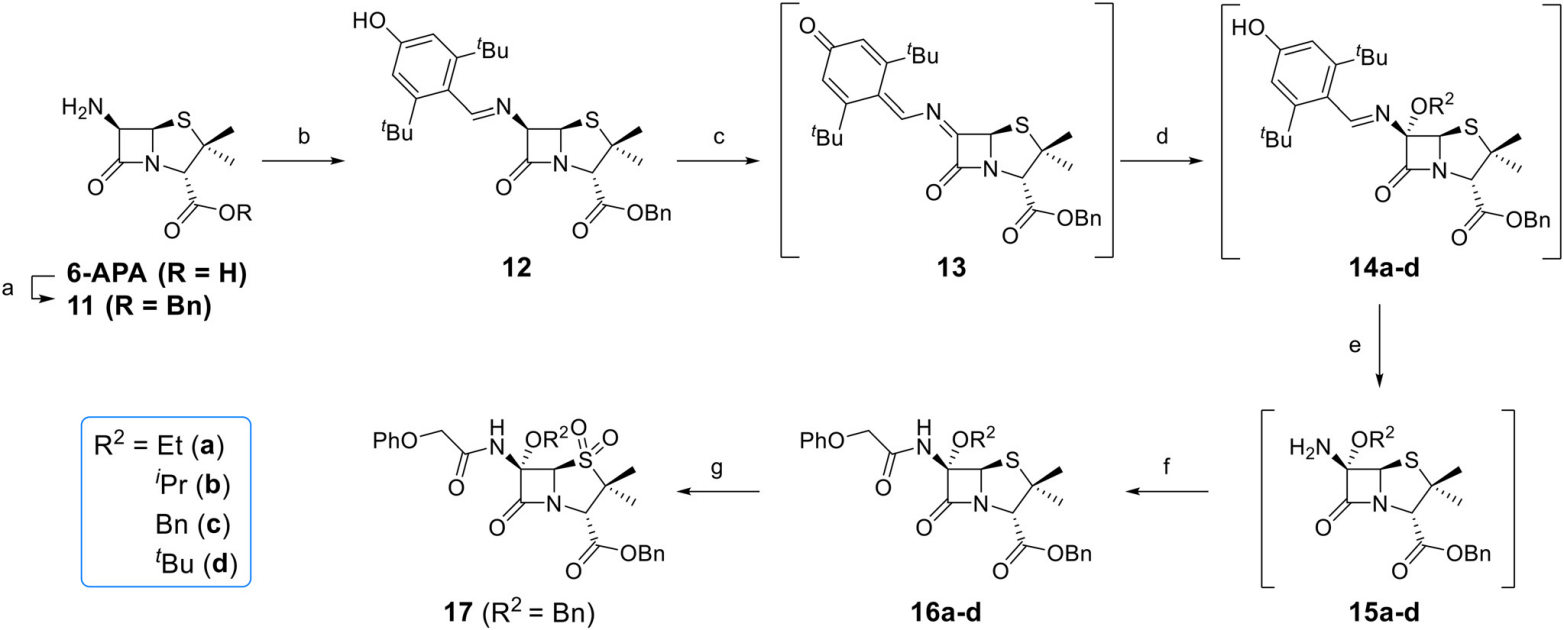
Synthesis of C6-alkoxy penicillin V derivatives. Reagents and conditions: a) BnBr (1.1 equiv.), Et_3_N (1.1 equiv.), acetone, 0 °C, 4 h, 33%; b) 3,5-di-*tert*-butyl-4-hydroxy-benzaldehyde,^51^ benzene, reflux, 1 h; c) 2,3-dichloro-5,6-dicyano-1,4-benzoquinone^51^ (0.9 equiv.), CH_2_Cl_2_, rt, 1 h; d) ROH (10 equiv.), CH_2_Cl_2_, rt, 21 h; e) trimethylacetohydrazideammonium chloride (0.8 equiv.),^51^ MeOH, CH_2_Cl_2_, rt, 3 h; f) PhOCH_2_Cl_2_ (5 equiv.), Et_3_N (7 equiv.), CH_2_Cl_2_, rt, 16 h, 1–12% (4 steps); g) *m*CPBA (2.2 equiv.), CH_2_Cl_2_, 0–10 °C, 1 h, 21%.

Attempts to convert 1 to the corresponding C6-*tert*-butoxy derivative 10c using LiO^*t*^Bu/^*t*^BuOH failed, perhaps reflecting the increased steric bulk of *tert*-butoxide, an observation which reveals the limitation of this method for preparation of C6-alkoxy penicillins. An alternative route to C6-*tert*-butoxy penicillin derivatives *via* imine 13 was trialled (Fig. 5); 13 is a reported intermediate in the synthesis of related C6-alkoxy and C6-formamido penicillins.^51,52^ However, imine 13 and related reaction intermediates were unstable/challenging to purify, resulting in poor reproducibility. Although the C6-benzyloxy penicillin 16c was obtained (12% yield from 12 over 4 steps) *via* this route, the corresponding C6-ethoxy and C6-iso-propoxy derivatives 16a and 16b were obtained in <1% yield, while the C6-*tert*-butoxy derivative 16d could not be obtained; 16c was converted to the corresponding C6-benzyloxy penicillin V sulfone benzyl ester 17 using *m*CPBA.

SPE-MS assay^40^ results revealed that C6-alkoxy penicillin substituents which were sterically bulkier than a methoxy group restored M^pro^ inhibition compared to the inactive C6-methoxy penicillin sulfone derivative 17 (Table 1, entries vii–ix). The C6-ethoxy (10a), C6-benzyloxy (17), and C6-alkoxy 10b penicillin sulfones manifested similar levels of M^pro^ inhibition (Table 1, entries vii–ix), whereas the C6-benzyloxy penicillin V sulfide 16c, like 9, was inactive. SPE-MS assays revealed that none of the penicillin V derivatives bearing C6-alkoxy substituents covalently reacted, at least efficiently, with M^pro^ (Fig. S9). The low levels of reaction observed in some cases (*e.g.*, 7; Fig. 4A) may reflect non-selective reaction with one or more of the 12 cysteine residues in M^pro^.^40,42^

**Fig. 6:**
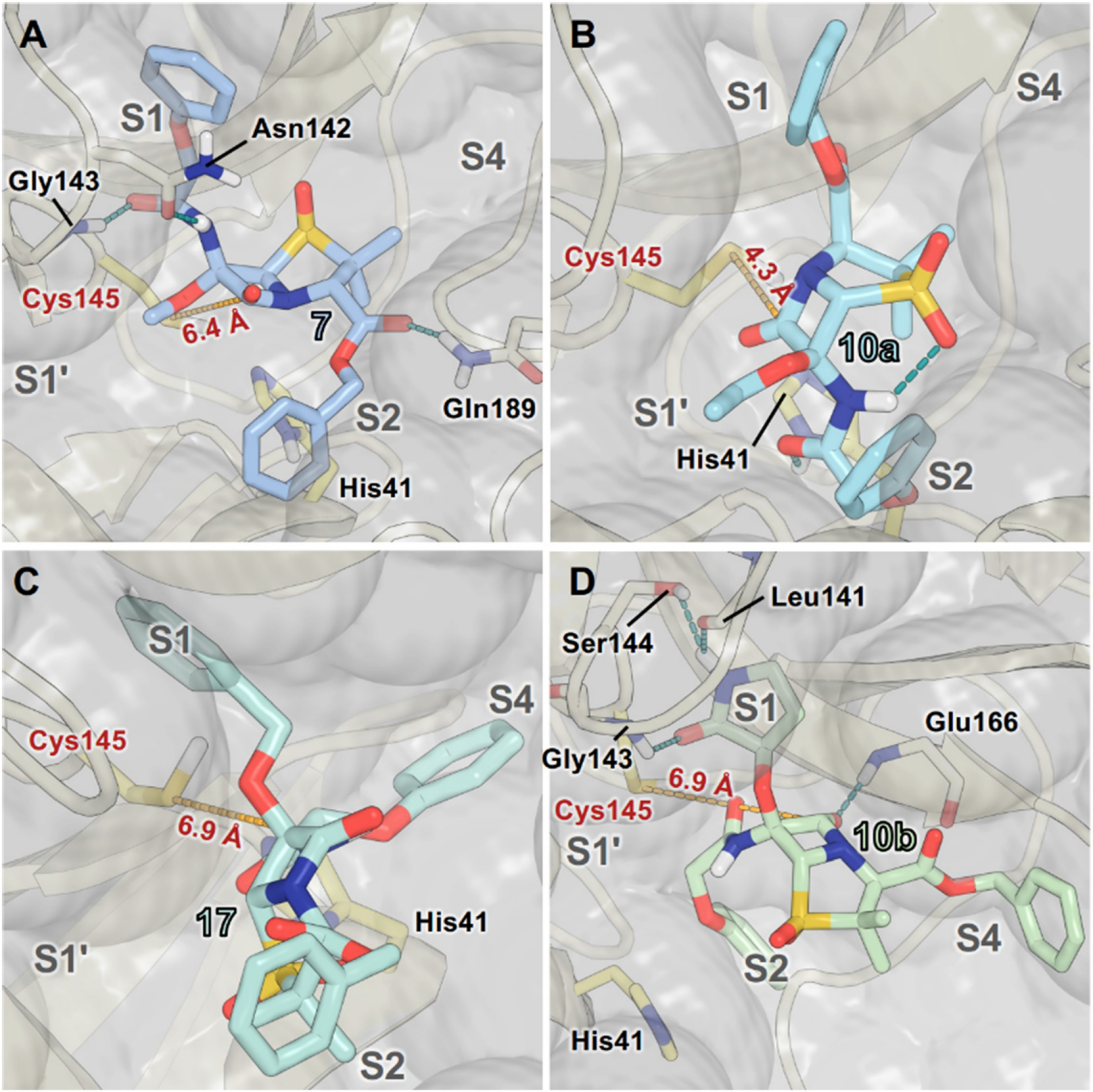
Representative M^pro^ binding modes of selected penicillin V derivatives from cluster analysis of MD simulations. (A) The C6-amide and the C6-methoxy groups of penicillin V derivative 7 are predicted to occupy the S1 and S1′ pockets, respectively, with the C3-ester in the S2 pocket. 7 is predicted to form hydrogen bonds with Asn142, Gly143, and Gln189. (B) The C6-amide group of the penicillin V derivative 10a is predicted to occupy the S2 pocket, while its C6-ethoxy and C3-ester groups occupy the S1′ and S1 sites, respectively. 10a is predicted to interact with His41 and to form an intramolecular hydrogen bond between its C6-amido N–H and the pro-*R*-sulfone O. (C) The C6-benzyloxy group of penicillin V derivative 17 is predicted to occupy the S1 site and its C6-amide the S4 site. (D) The C6-γ-lactam group of penicillin V derivative 10b is predicted to occupy the S1 pocket, its C6-amide the S2 pocket, and its C3 ester the S4 pocket. 10b is predicted to hydrogen bond with Leu141, Gly143, Ser144, and Glu166. The S(Cys145)–C(O)(β-lactam) distances for the representative snapshots are in red (dashed yellow lines), with average values from MD simulations provided in Fig. S6. Atoms are coloured by element (N: blue, O: red, S: yellow, F: cyan, H: white), with C-atoms coloured differently for each penicillin derivative. Active site His41 and Cys145 side chain C-atoms are in bright yellow; C-atoms of other residues are in pale yellow. MD simulations were performed for 3 × 100 ns.

MD simulations indicate that changing the S-oxidation state, *e.g.*, to a sulfoxide (7), or increasing the steric bulk of the C6-alkoxy substituent beyond methoxide, *e.g.*, to the ethoxy (10a), benzyloxy (17), and γ-lactam bearing C6-ether (10b) will alter the binding mode at the M^pro^ active site (Fig. 6A–D), rationalising differences in the observed IC_50_ values. The modelling results predict that the C6-benzyloxy group of 17 binds in the S1 pocket, whereas the C6-methoxy group of 7 and the C6-ethoxy group of 10a bind in the S1′ pocket. Importantly, however, none of these compounds are predicted to be oriented for productive covalent reaction with Cys145 (Fig. S6), in accord with the MS assay results (Fig. 5). Note that the modelled pose of 10b involves binding of its γ-lactam moiety in the S1 pocket, in an analogous manner to the γ-lactam of nirmatrelvir (Fig. S4).^6^

### C6-Methoxy penicillin G (*R*)-sulfoxide ester derivatives are potent M^pro^ inhibitors

**Fig. 7:**
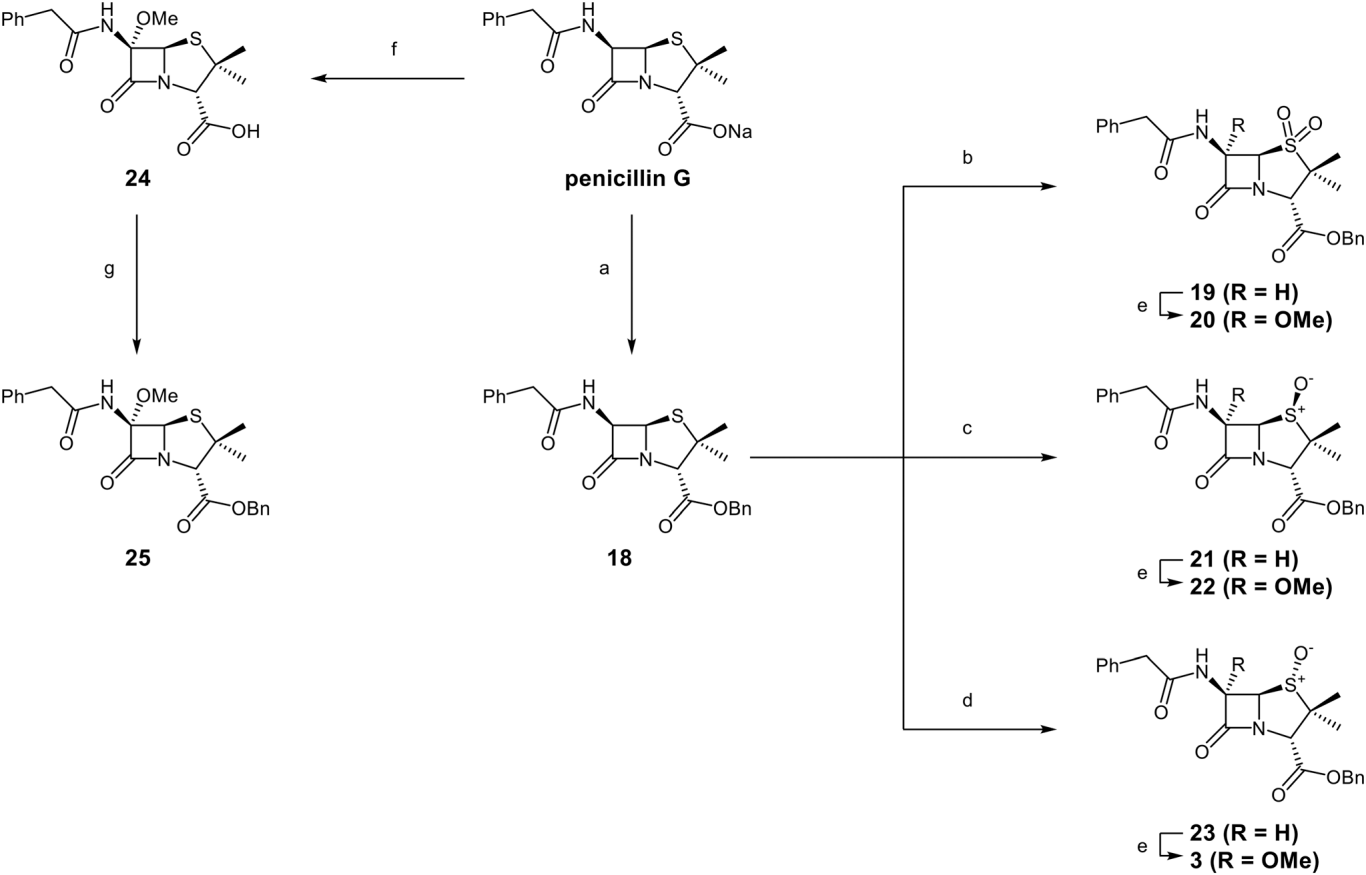
Synthesis of C6-methoxy penicillin G derivatives. Reagents and conditions: a) BnBr (1.2 equiv.), *N*,*N*-dimethylformamide, rt, 16 h, 84%; b) *m*CPBA (2.5 equiv.), CH_2_Cl_2_, 0 °C to rt, 16 h, 55%; c) *m*CPBA (1.1 equiv.), CH_2_Cl_2_, 0 °C, 1 h, 83%; d) 2,6-lutidine (2.4 equiv.), ^*t*^BuOCl (1.1 equiv.), THF : H_2_O (10 : 1), −5 °C, 5 min, 22%; e) ^*t*^BuOCl (1.5 equiv.), LiOMe (1 M in MeOH; 1.1 equiv.), CH_2_Cl_2_, −30 °C, 1 h, 36–54%; f) ^*t*^BuOCl (1.5 equiv.), LiOMe (1 M in MeOH; 1.1 equiv.), *N*,*N*-dimethylformamide : MeOH (7 : 1), −78 °C, 15 min, 18%; g) BnBr (1.4 equiv.), NaHCO_3_ (1.5 equiv.), *N*,*N*-dimethylformamide, rt, 16 h, 49%.

A set of C6-methoxy penicillin G derivatives was then synthesised to investigate the effects of the C6-phenylacetamido ether oxygen on M^pro^ inhibition, because previous studies have shown that both the position and the presence/absence of the C6 side chain oxygen can influence potency.^41^ Modelling studies and reported work on penicillin V derivatives imply that the ester group does not substantially affect inhibition potency (Fig. 2A and D);^40,41^ therefore, penicillin G benzyl ester derivatives were synthesized for comparison with the penicillin V benzyl esters, following the synthesis of the corresponding C6-methoxy penicillin V derivatives (Fig. 7). Penicillin G was alkylated to give the reported benzyl ester 18,^53^ which was oxidized using *m*CPBA to give sulfone 19 or (*S*)-sulfoxide 21. The isomeric (*R*)-sulfoxide 23 was obtained from 18 in 22% yield by oxidation using ^*t*^BuOCl ^54^ in the presence of 2,6-lutidine. C6-Methoxylation of penicillin G derivatives 19, 21, and 23 yielded penams 20, 22, and 3, respectively, using ^*t*^BuOCl and LiOMe in methanol (1 M) at −30 °C (36–54%). A related protocol enabled the direct conversion of penicillin G to the C6-methoxypenicillin G acid 24, though in lower yield (18%). Subsequent alkylation of 24 gave ester 25 (49%).

The results of SPE-MS assays reveal that the penicillin G derivatives without a C6-alkoxy group do not inhibit M^pro^ (Table 2, entries i–v), as reported,^40,41^ likely reflecting knowledge that the phenoxy oxygen atom is important in M^pro^ inhibition (Fig. 2). By contrast, the corresponding penicillin G derivatives bearing a methoxy group at the C6 position inhibited M^pro^ (Table 2, entries vii–xi).

**Table 2:** Effects of penicillin G derivatives on SARS-CoV-2 M^pro^ catalysis

	Penicillin G derivative	IC_50_^a^ [μM]		C6-Methoxy penicillin G derivative	IC_50_^a^ [μM]
i	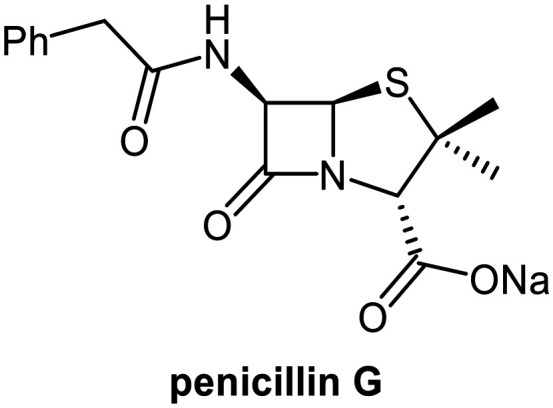	>50 (reported:^40^ >50)	vii	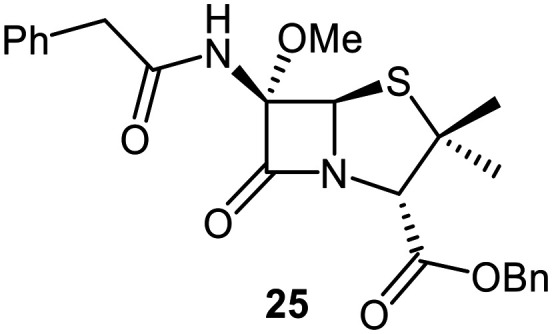	11 ± 6
ii	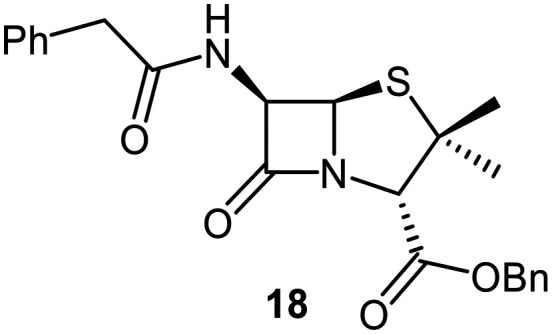	>50	viii	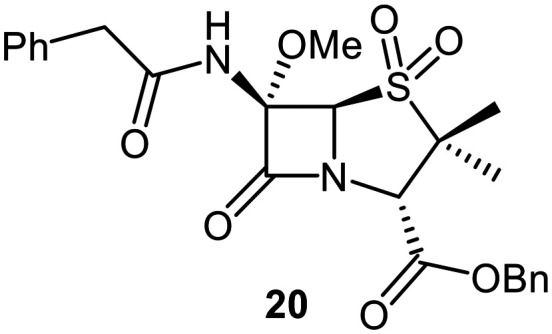	11 ± 4
iii	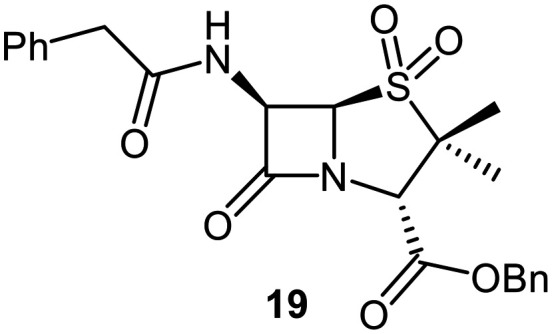	>50	ix	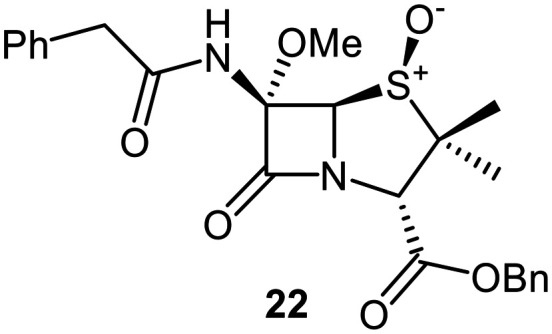	7.3 ± 6.5
iv	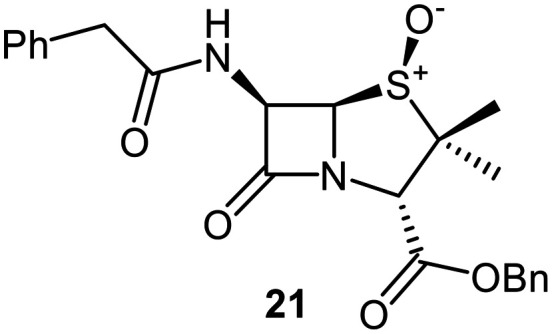	>50	x	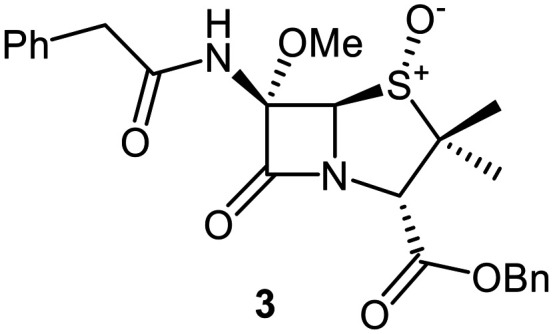	0.8 ± 0.1
v	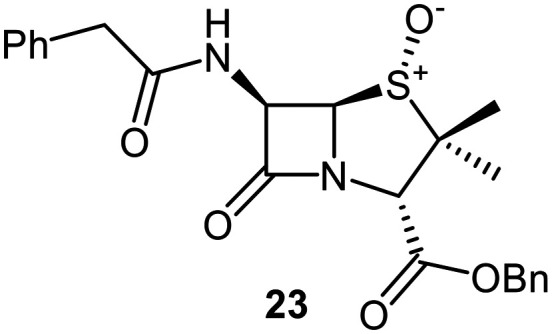	>50	xi	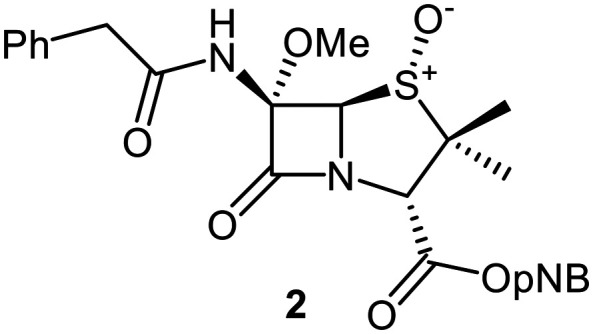	0.5 ± 0.1 (reported:^40^ ∼3.5)
vi	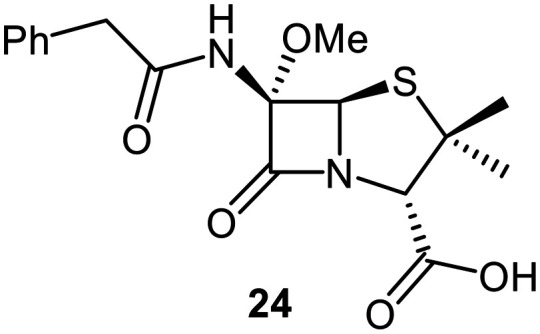	>50^b^			

a M^pro^ inhibition assays were performed using SPE-MS as reported,^12,40^ utilizing SARS-CoV-2 M^pro^ (0.05 μM) and substrate (2.0 μM). Results are means of three independent experiments (*n* = 3; mean ± SD), each in technical duplicates.

b Representative dose–response curves are shown in Fig. S7. Bn: –CH_2_Ph; *p*NB: –CH_2_C_6_H_4_(4-NO_2_).

The penam sulfide 25, (*S*)-sulfoxide 22, and sulfone 20 inhibited M^pro^ with similar potencies (Table 2, entries vii–ix), whereas the C6-methoxy (*R*)-sulfoxide penicillin G benzyl ester 3 inhibited M^pro^ ∼9-fold more efficiently than its (*S*)-sulfoxide isomer 22 (IC_50_ ∼ 0.8 and 7.3 μM, respectively; Table 2, entries x and ix). Note that variations in the IC_50_ values for the C6-methoxy penicillin G derivative 24 were observed (Table 2; entry vi), with moderate inhibition potency being observed in some cases (IC_50_ = 15 ± 8 μM), possibly due to instability of 24 in DMSO, as also noted with 8 (see above).

Protein-observed MS studies^40^ showed that the tested penicillin G derivatives 2, 3, and 18–25 did not covalently modify M^pro^, even after relatively long incubation times of >15 h (Fig. S9E-G), consistent with the MD simulations indicating that penicillin C6-methoxy substituents increased the average distances between the β-lactam carbonyl and the Cys145 thiol (Fig. 2B and S5B).

Interestingly, the penicillin G (*R*)-sulfoxides 2 and 3 were both observed to covalently react with M^pro^ by SPE-MS;^40^ however, +515 Da and +470 Da mass shifts corresponding to acyl–enzyme complex formation were not observed with high intensity, even after >15 h incubation (Fig. 4C and D). By contrast, concentration-dependent formation of a +32 Da M^pro^ adduct was observed with both 2 and 3 (Fig. S10). The combined results indicate that the formation of the +32 Da M^pro^ adduct depended on the sulfoxide configuration, but apparently not (or at least to a lesser extent) on the nature of the penicillin ester. Note that re-analysis of the reported MS spectrum of the M^pro^ reaction with 2 also evidences the formation of a +32 Da adduct after >60 min, albeit at relatively low levels.^40^

**Fig. 8:**
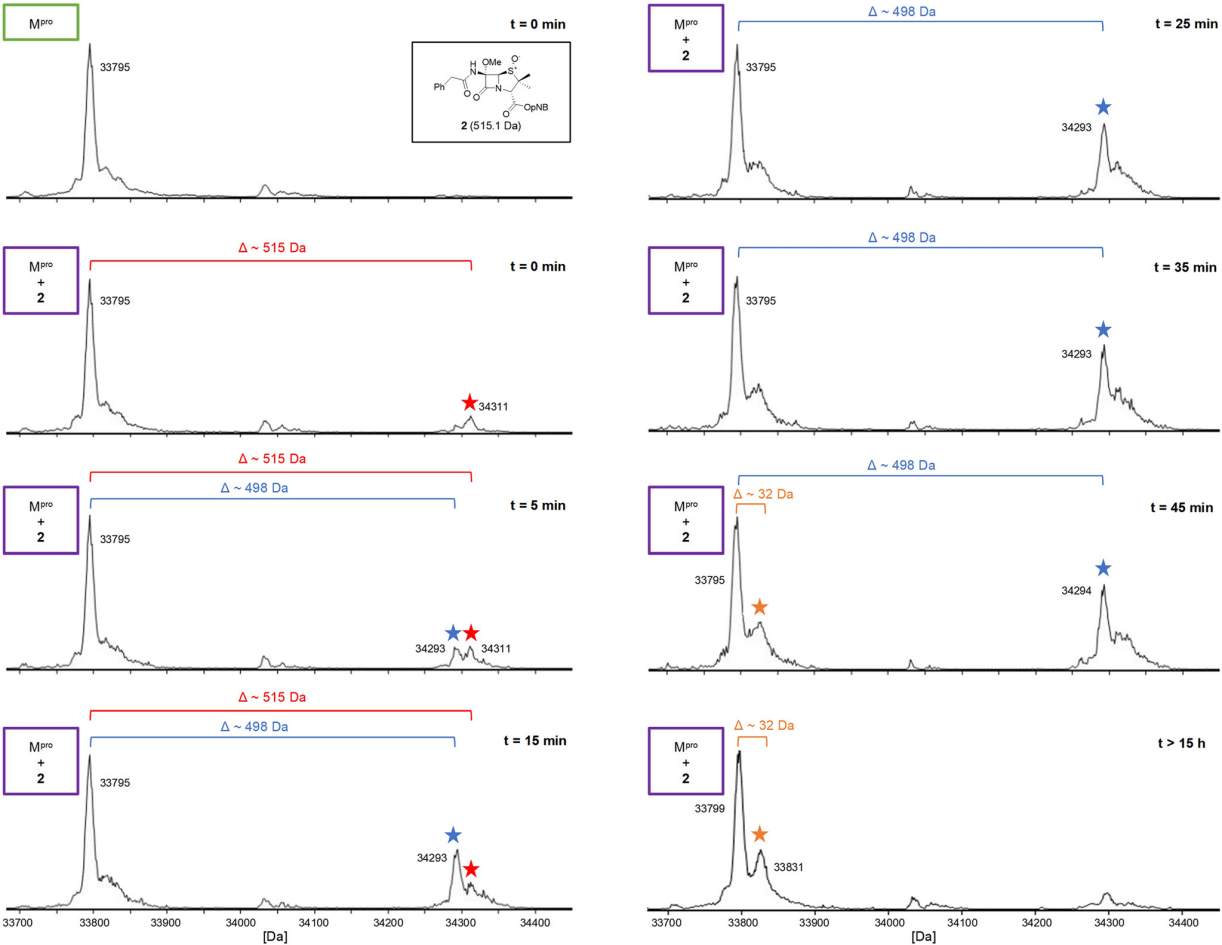
Evidence for covalent reaction of M^pro^ with the C6-methoxy penicillin G (*R*)-sulfoxide derivative 2. SPE-MS analysis showed initial formation of a +515 Da adduct (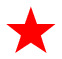) at low levels, followed by the time-dependant formation of a +498 Da adduct (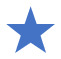) (5 : 1 inhibitor : M^pro^ ratio; data obtained using a 10 : 1 inhibitor : M^pro^ ratio conditions is available in the SI (Fig. S11). A possible low intensity +32 Da adduct (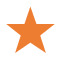) was also detected after 45 min incubation. *p*NB: –CH_2_C_6_H_4_(4-NO_2_).

To investigate the relevance of the +32 Da M^pro^ adduct to inhibition, we monitored the covalent reaction of the penicillin derivative 2 with M^pro^ over ∼45 min using SPE-MS (Fig. 8), because the IC_50_ values were determined following 45 min incubations of M^pro^ with penicillin derivatives (Tables 1 and 2). A +515 Da mass shift of consistently weak intensity was observed (*t* = 0 min), corresponding to the mass of 2. A +498 Da adduct of increasing intensity was formed within 15 min, likely corresponding to dehydrated 2 covalently attached to M^pro^. After 45 min, low levels of a +32 Da adduct were visible. If the enhanced inhibition with the (*R*)-sulfoxides corresponds to covalent reaction, it is likely not a result of the +32 Da species, but of the dehydrated +498 Da species which may, over time, convert to the +32 Da species (Fig. 8 and S10). Note that the ∼9-fold increases in M^pro^ inhibition potency for the (*R*)-sulfoxides 2 and 3 compared to the (*S*)-sulfoxide 22 apparently manifested in no evidence for covalent M^pro^ modification with the latter (Fig. S9F).

### Effects of C6-alkoxy penam and sulbactam derivatives on M^pro^ inhibition

**Fig. 9:**
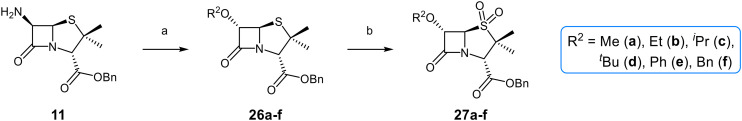
Synthesis of monosubstituted C6-alkoxy penam derivatives. Reagents and conditions: a) i) NaNO_2_ (2.6 equiv.), *para*-toluenesulfonic acid monohydrate (0.6 equiv.), CH_2_Cl_2_ : H_2_O (4 : 1), 0 °C, 20 min; ii) ROH (1.6–110 equiv.), BF_3_·O(C_2_H_5_)_2_ (0.1 equiv.), CH_2_Cl_2_, 20 °C, 15 min, 15–37% (two steps); b) *m*CPBA (2.2 equiv.), CH_2_Cl_2_, 0 °C to rt, 16 h, 28–77%.

The effects of removing the C6-acetamido substituent of the C6-alkoxy penicillin sulfone derivatives on M^pro^ inhibition potency and reactivity were investigated. The mono-substituted C6-alkoxy penam sulfides 26a–f were synthesized in two steps from penam 11 (15–37%), *via* stereospecific reactions of a C6-diazo penicillin intermediate with different alcohols in the presence of BF_3_ etherate complex.^55,56^ Sulfides 26a–f were oxidized to sulfones 27a–f using *m*CPBA (Fig. 9). The (*S*)-sulfoxides were not synthesized due to a lack of reported stereoselective methods to oxidize penams in the absence of a directing C6-amido substituent.

The results of SPE-MS assays indicate that C6-alkoxy substituted penams in the sulfide oxidation state (*i.e.*, 26a–f) typically inhibited M^pro^ with higher potency than those in the sulfone oxidation state (27a–f) (Table 3). Penicillins bearing sterically less bulky and/or conformationally relatively flexible C6-alkoxy substituents, such as the C6-methoxy (26a), -ethoxy (26b), and -benzyloxy (26f) derivatives, showed higher inhibition (Table 3, entries i, ii, and vi, respectively) than those bearing bulky and rigid substituents (*i.e.*, derivatives 26c–e; Table 3, entries iii–v).

**Table 3:** Effects of penicillin V derivatives without a C6 amido group on SARS-CoV-2 M^pro^ catalysis

	C6-Alkoxy penam derivative	IC_50_^a^ [μM]		C6-Alkoxy sulbactam derivative	IC_50_^a^ [μM]
i	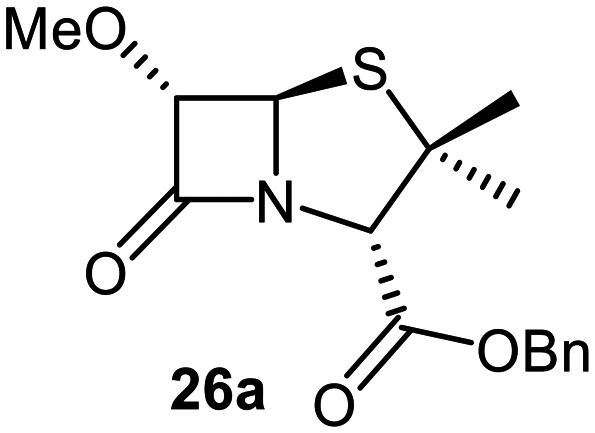	1.2 ± 0.1	vii	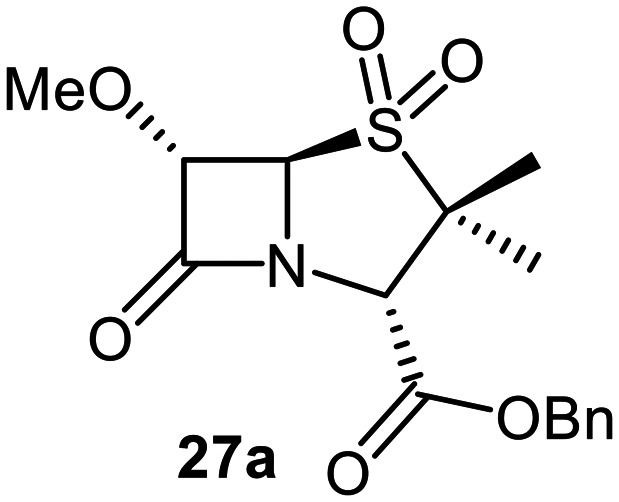	7.7 ± 4.9
ii	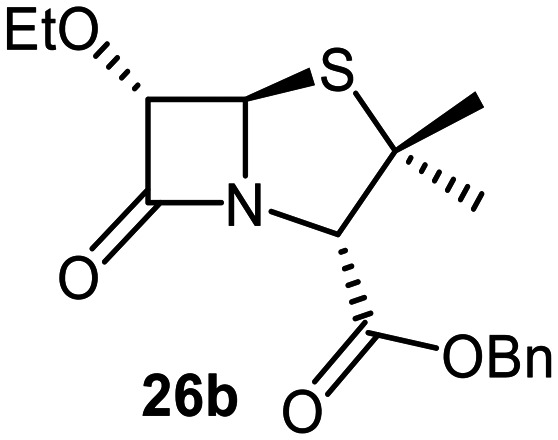	0.8 ± 0.1	viii	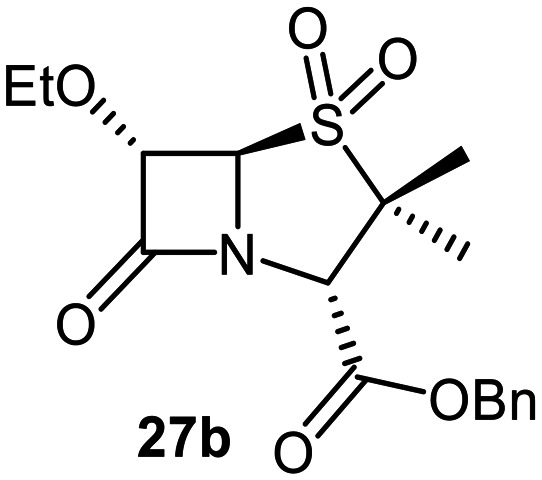	44 ± 4
iii	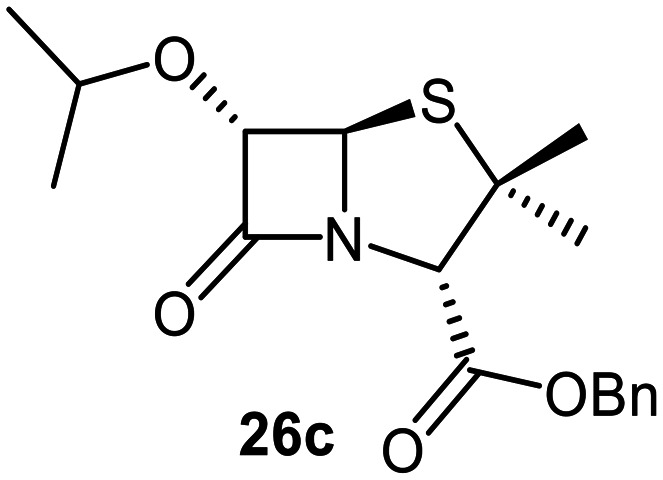	47 ± 13	ix	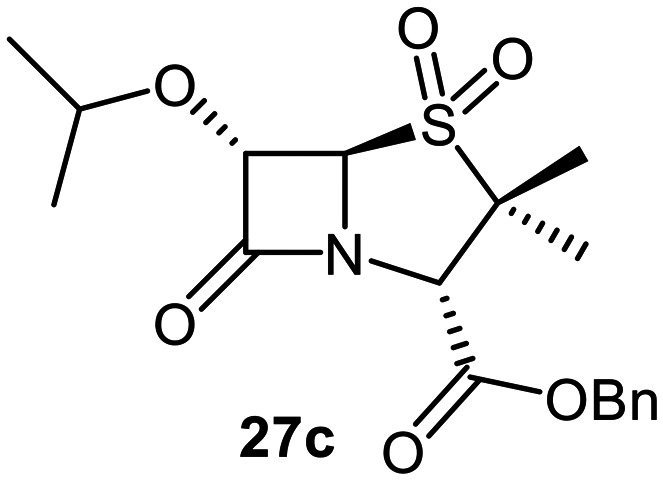	19 ± 14
iv	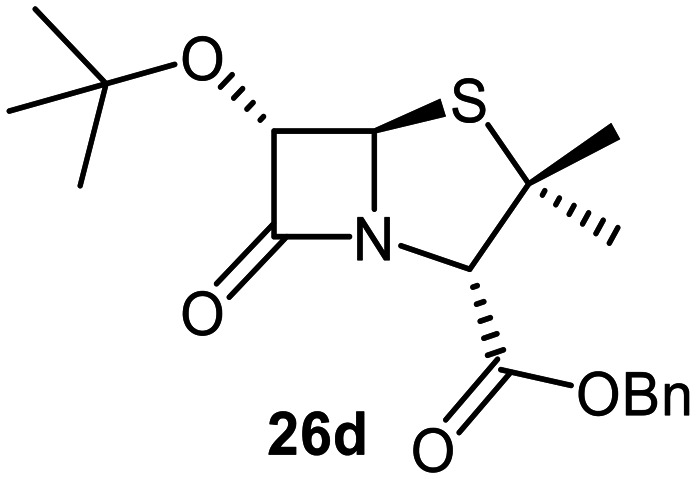	39 ± 9	x	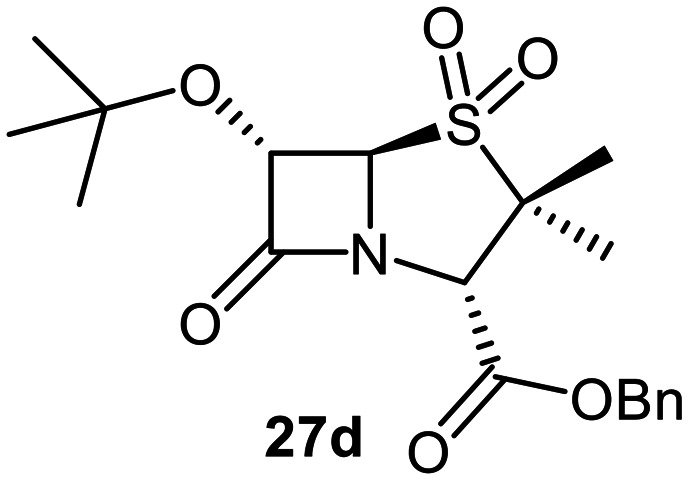	21 ± 3
v	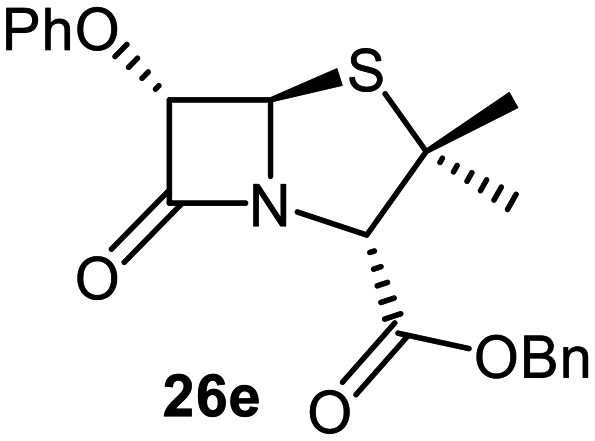	>50	xi	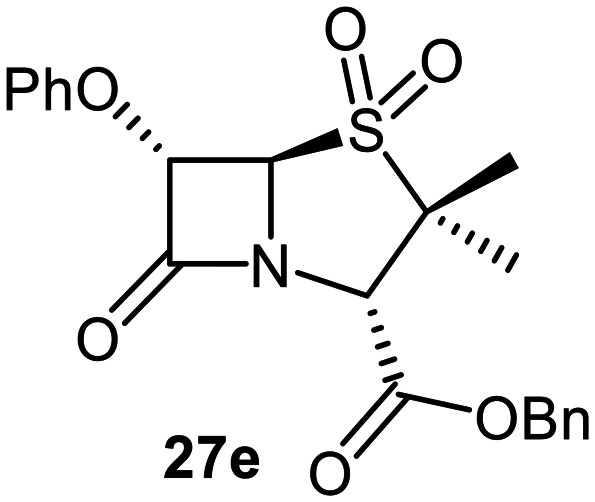	>50
vi	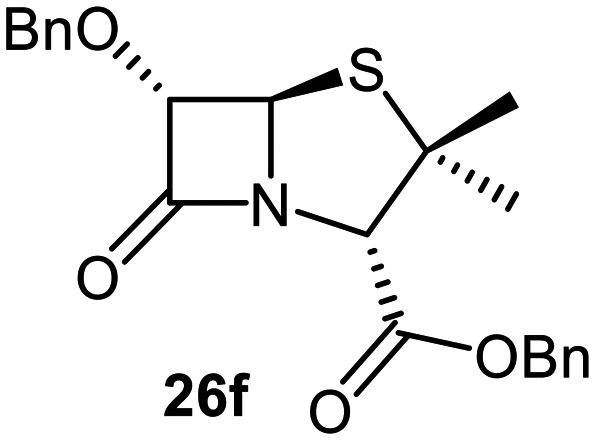	7.3 ± 1.4	xii	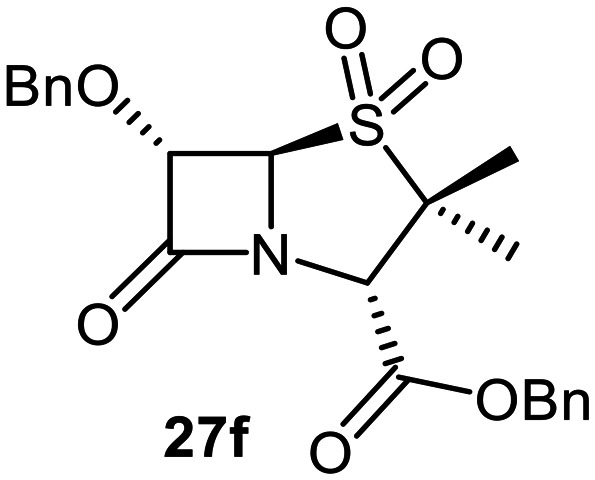	35 ± 2

a M^pro^ inhibition assays were performed using SPE-MS as reported,^12,40^ utilizing SARS-CoV-2 M^pro^ (0.05 μM) and substrate (2.0 μM). Results are means of three independent experiments (*n* = 3; mean ± SD), each in technical duplicates. Representative dose–response curves are shown in Fig. S7. Bn: –CH_2_Ph.

**Fig. 10:**
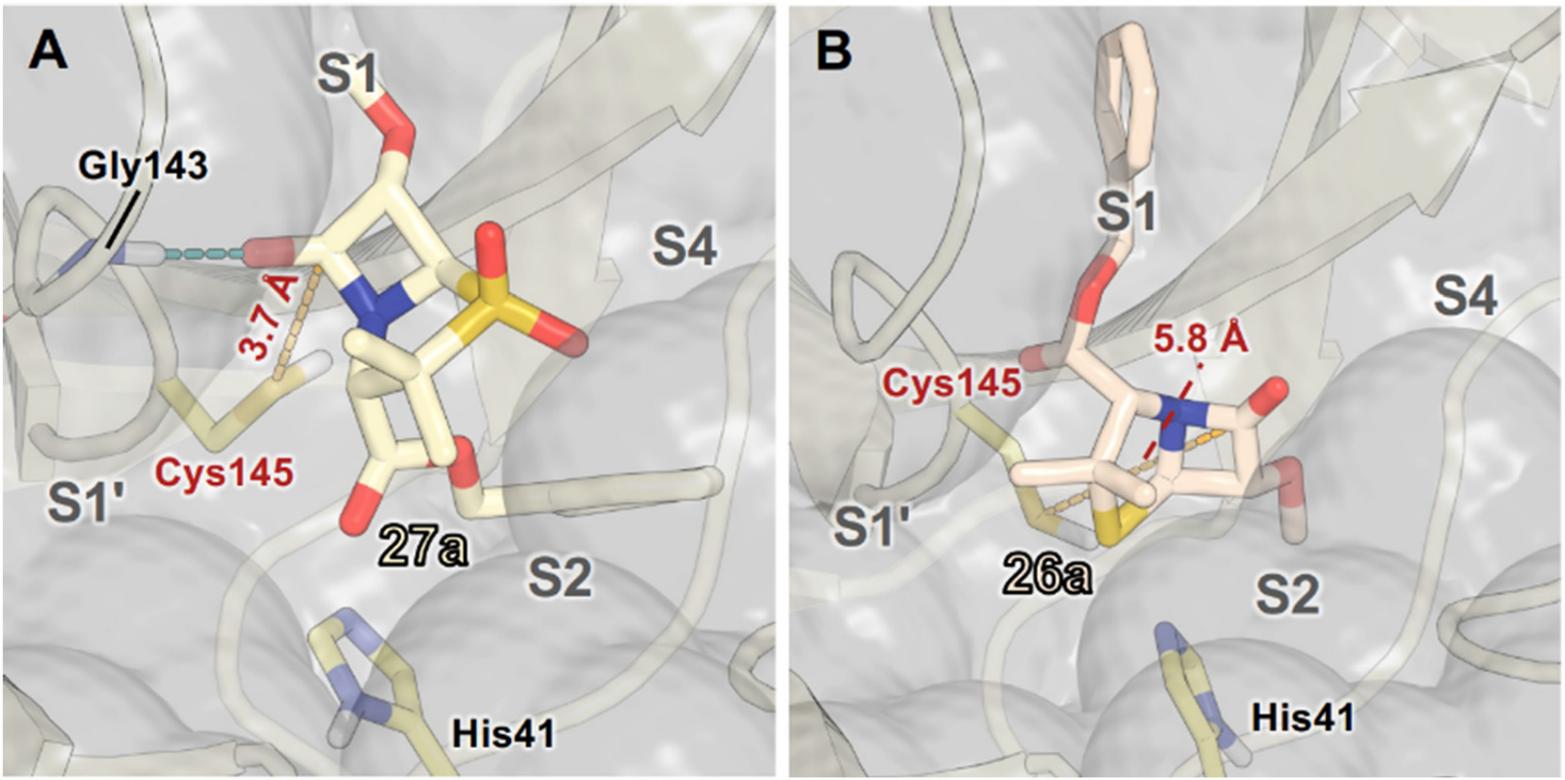
Representative poses of C6-methoxy penam sulfones with M^pro^ from cluster analysis of MD simulations. (A) The C6-methoxy and the C3-ester groups of sulfone 27a are predicted to occupy the M^pro^ S1 and S2 pockets, respectively; (B) while they are predicted to occupy the M^pro^ S2 and S1 pockets for sulfide 26a. 27a is predicted to interact with Gly143, whereas 26a did not appear to form a stable hydrogen bond with Gly143. The S(Cys145)–C(O)(β-lactam) distances for these representative snapshots are in red (dashed yellow lines), with average values from MD simulations provided in Fig. S7. Atoms are coloured by element (N: blue, O: red, S: yellow, F: cyan, H: white), with C-atoms coloured differently for each penicillin derivative. Active site His41 and Cys145 side chain C-atoms are in yellow; C-atoms of other residues are in pale yellow. MD simulations were performed for 3 × 100 ns.

Modelling of 27a at the M^pro^ active site predicted stable binding poses (Fig. 10A), but with an average S(Cys145)–C(O)(β-lactam) distance of 9.0 Å (Fig. S7), likely hindering covalent reaction with M^pro^. In accord with the modelling predictions, SPE-MS analyses did not provide evidence for covalent reaction of the penam sulfides 26a–f and sulfones 27a–f with isolated M^pro^ (Fig. 4E and F and S9H–O). While the C6-methoxy and the C3-ester groups of 27a are predicted to bind in the S1 and S2 pockets, respectively, the sulfone oxygens are predicted not to directly interact with M^pro^, in agreement with the higher potency of the sulfide oxidation state. Notably, the predicted pose of the C6-methoxy penam sulfide 26a (Fig. 10B) substantially differed from that of 27a, suggesting that the observed lower potency of the sulfones 27a–f relative to sulfides 26a–f may arise from the adoption of distinct active site binding modes.

## Discussion

β-Lactam antibiotics are widely used for treatment of bacterial infections.^39^ There is extensive evidence with isolated enzymes and with cells that their antibacterial activity is a result of covalent reaction with a nucleophilic serine residue in bacterial transpeptidases, leading to formation of stable acyl–enzyme complexes *via* β-lactam ring opening with consequent inhibition of bacterial cell wall biosynthesis.^39,57^ The continued widespread use of β-lactam antibiotics is *inter alia* threatened by resistance mediated by serine β-lactamases (SBLs), which are nucleophilic serine enzymes related to transpeptidases, but which catalyse β-lactam degradation *via* hydrolytically labile acyl–enzyme complexes.^37,58^

Penicillins bearing a C6-methoxy substituent have been developed with the aim of countering antibiotic resistance by SBLs:^59–61^ temocillin,^44^ the clinically used C6-methoxy derivative of ticarcillin,^62^ shows increased resistance towards SBL-catalysed β-lactam ring opening.^45,63^ The same strategy to provide β-lactam stabilization is also used with cephalosporin antibiotics (*i.e.*, cephamycins; Fig. S1), which bear a C7-methoxy group (the penicillin C6-equivalent position).^39,46,64,65^ The precise reasons for the decreased SBL reactivity with penicillins/cephalosporins bearing a C6/C7-methoxy group are unclear, but may reflect that the increased steric hindrance adjacent to the β-lactam carbonyl group slows the rate of hydrolysis of the acyl–enzyme complex, possibly by blocking the access of the hydrolytic water to the acyl–enzyme carbonyl group.^45^

Although β-lactam antibiotics are best known for their covalent inhibition of bacterial transpeptidases, their inhibitory activity is not solely dictated by ring strain and electrophilicity. Their ability to mimic transition-state conformations during amide hydrolysis may also contribute to target binding and specificity.^57,66,67^ In this light, appropriately substituted β-lactams have potential to act as competitive, non-covalent inhibitors of serine and cysteine proteases. Indeed, it cannot be entirely ruled out that some of the antibiotic activity of penicillins and other β-lactams results from non-covalent inhibition. Intact penicillins have been observed in complex with bacterial transpeptidases when the enzymes were inactivated at the active site either by cross-linking or mutation.^68,69^ The proposal is precedented by the development of substituted monocyclic β-lactams which engage with protein targets through non-covalent interactions, including *e.g.*, ezetimibe (Fig. S12A).^70,71^ Furthermore, penicillins and cephalosporins with appropriately-substituted C6-amido sidechains (Fig. S12B) are reported to inhibit the Speckle-type POZ protein (SPOP)^72^ potentially *via* non-covalent binding that disrupts SPOP–substrate interactions.^73,74^

As part of a wider search for potent SARS-CoV-2 antiviral agents, we and others have reported on the utility of β-lactams and related electrophiles for SARS-CoV-2 M^pro^ inhibition.^40,41,43,75–77^ Although our previous work mainly focused on development of covalently reacting β-lactam containing M^pro^ inhibitors, including penicillin derivatives,^40,41^ we obtained evidence showing the potential for penicillin derivatives to inhibit M^pro^*via* non-covalent binding at the active site.^40^ The combined results described here indicate that apparently minor structural modification to the penicillin substitution pattern and sulfur oxidation state can affect not only M^pro^ inhibition potency, but also the mechanism by which penicillins inhibit M^pro^ (Fig. 2, 4, 6, 8 and 10).

Modelling and MS analyses reveal that, at least in most cases, C6-alkoxy substituted penicillin V and G derivatives do not directly react with isolated M^pro^ to give covalent adducts *via* β-lactam ring opening, but efficiently bind to the M^pro^ active site to inhibit catalysis *via* competing with the substrate for binding (Fig. 4 and S9). The modelling studies predicted that the penicillin C6-alkoxy substitution increases the distance between the electrophilic β-lactam carbonyl C-atom and the nucleophilic thiolate S-atom of Cys145, thus impairing efficient covalent reaction (Fig. 2, 6 and 10).

Interestingly, our results reveal that penicillin C6-alkoxy derivatives have capacity to covalently react with M^pro^, if apparently stringent requirements on penicillin substitution pattern, penicillin sulfur oxidation state, and configuration of penicillin stereocentres are matched (Fig. 8 and 9). Monitoring the reaction of isolated M^pro^ with penicillin derivative 2 using SPE-MS revealed formation of a covalent M^pro^ adduct corresponding to penicillin dehydration within 45 min in a concentration dependent manner (Fig. 8), which apparently slowly reacted (within >15) h to give a +32 Da M^pro^ species (Fig. S10). Furthermore, covalent adduct formation apparently correlated with ∼9-fold increase in inhibition potency compared to isomeric penicillin derivatives which did not promote covalent adduct formation (Table 2, entries x and xi).

The observed +32 Da mass shift could be the result of cysteine thiol oxidation to give either a sulfinic acid (+2 O) or a persulfide (+ S); these modifications cannot be distinguished using SPE-MS due to insufficient resolution. Nonetheless, cysteinyl thiol oxidation to sulfinic acids and persulfides are known post-translational modifications,^78,79^ and the formation of persulfidated M^pro^ is precedented by crystallographically observed M^pro^ disulfide adducts derived from thiocyanate-based inhibitors,^80^ and further supported by reactions of Cys145 (and other cysteine residues^42^) with ebselen derivatives to give sulfur–selenium (S–Se) adducts.^81,82^

A possible mechanism for the reaction of M^pro^ with C6-methoxy penicillin G (*R*)-sulfoxide esters 2 and 3 involves C3 deprotonation of 2 at the M^pro^ active site, followed by thiazolidine ring opening to form a sulfenic acid^83^ (Fig. S13, I); the base-catalysed thiazolidine ring opening of related penicillin sulfone ester derivatives *via* C3 deprotonation is known.^84^ The reaction of penicillin sulfenic acid intermediates with thiols to give mixed disulfides is also documented;^85–87^ the sulfenic acid intermediate I may further react with a cysteine thiol to generate a mixed disulfide (+498 Da; Fig. S13, II), which may slowly eliminate to the persulfidated M^pro^ adduct (+32 Da; Fig. S13, III). Note that we did not accrue evidence for selective reaction of Cys145, and that the nature of this +32 Da adduct is subject to ongoing investigations.

Beyond their applications as antibiotics, C6-alkoxy substituted penicillin derivatives have been reported to target non-bacterial enzymes: the C6-alkoxy penam sulfones 27a, 27b, and 27f are known covalent inhibitors of human leukocyte elastase (HLE),^88^ a serine protease implicated in inflammatory conditions.^89^ Interestingly, in contrast to their reported reactivity with HLE, C6-alkoxy penams (*e.g.*, 26a and 27a) did not evidence covalent modification of M^pro^, exhibiting a similar pattern to the C6-alkoxy penicillin V and G derivatives investigated in this study.

## Conclusion

Almost a century following Fleming's observation of the activity of penicillins,^90^ β-lactam containing antibacterials remain amongst the most important medicines.^39^ Our combined results further demonstrate the largely untapped potential of penicillin and related scaffolds for targeting enzymes other than those involved in bacterial cell wall biosynthesis, as first evidenced by work on elastase inhibition^88,91,92^ and more recently in work on M^pro^ inhibition.^40,41^ There is strong evidence that clinically used β-lactam antibiotics inhibit transpeptidases *via* mechanisms involving covalent reactions to form stable acyl–enzyme complexes (though it cannot be ruled out that some of the antibiotic activity of β-lactams results from non-covalent inhibition). In some cases, the initially formed complexes can undergo further reaction that increase stability of the acyl–enzyme complexes, as reported with SBL inhibitors, including clavulanic acid and related compounds.^93,94^ The work presented here highlights the potential of non-covalent, non-acylating enzyme inhibition mechanisms for penicillins and other bicyclic β-lactams. One potential application of these mechanisms is to help enable inhibition of bacterial cell wall biosynthesis in a manner that is not prone to current mechanisms of resistance, in particular β-lactamase production or transpeptidase mutation.

## Author contributions

D.-G. M. and L. K. synthesized penicillin derivatives; L. B. performed assays; W. T. performed modelling under supervision of F. D.; E. S. and H. C. provided resources; L. B. and C. J. S. conceptualised and supervised the research; D.-G. M., L. B., and C. J. S. wrote the manuscript with help from all authors.

## Conflicts of interest

The authors declare no competing interests.

## Supplementary Material

MD-016-D5MD00789E-s001

## Data Availability

Experimental data (procedures, compound characterisations, and ^1^H and ^13^C NMR spectra of novel penicillin derivatives) supporting the results of this study are included in the article and supplementary information (SI). Molecular modelling details are described in the SI. Relevant simulation input files, scripts, and final poses are available in a GitHub repository (https://github.com/duartegroup/C6-alkoxy-penicillins). Supplementary information is available. See DOI: https://doi.org/10.1039/d5md00789e.
