# Supplementary material for: C6-Alkoxy substituted penicillins are potent non-covalently binding inhibitors of the SARS-CoV-2 main protease
Source: RSC Med Chem. 2025 Oct 27;16(12):6351–67. doi: 10.1039/d5md00789e (PMC12588090; doi:10.1039/d5md00789e)
Supplement: MD-016-D5MD00789E-s001 [file MD-016-D5MD00789E-s001.pdf]

Supplementary Information (SI) for RSC Medicinal Chemistry.
This journal is © The Royal Society of Chemistry 2025

Supporting Information

C6-Alkoxy substituted penicillins are potent non-covalently binding inhibitors of the SARS-CoV-2 main protease

Dorian-Gabriel Muntean[1], Wojtek Treyde[1], Linda Kinena[1,2], Eidarus Salah[1], Hani Choudhry[3], Fernanda Duarte[1], and Lennart Brewitz[1,*], and Christopher J. Schofield[1,*]

*Email: lennart.brewitz@chem.ox.ac.uk or christopher.schofield@chem.ox.ac.uk.

[1]Chemistry Research Laboratory and the Ineos Oxford Institute for Antimicrobial Research, University of Oxford, 12 Mansfield Road, OX1 3TA, Oxford, United Kingdom.

[2]Latvian Institute of Organic Synthesis, Aizkraukles 21, LV-1006 Riga, Latvia.

[3]Department of Biochemistry, Center for Artificial Intelligence in Precision Medicines, King Abdulaziz University, Jeddah, Saudi Arabia.

Table of contents

1. Supporting figures S2-S15
2. Computational methods S16-S19
3. General synthesis information S20-S21
4. General synthetic procedures S22-S23
5. Experimental procedures and compound characterisations S24-S43
6. References S44-S45
7. ^{1}H and ^{13}C NMR spectra of novel C6-alkoxy penicillin derivatives prepared for this study S46-S70

1. Supporting figures

Supporting Figure S1. Representative β-lactam antibiotics bearing a methoxy (MeO) group α to the β-lactam carbonyl group. A. C6-Methoxy penicillin: temocillin.[1] **B.** C7-methoxy cephalosporins: cephamycin C,[2] cefoxitin,[3] cefmetazole,[4] cefbuperazone,[5] cefotetan.[6] **C.** C7-methoxy oxacephalosporin: moxalactam.[7] **D.** C3-methoxy monobactam: sulfazecin.[8]

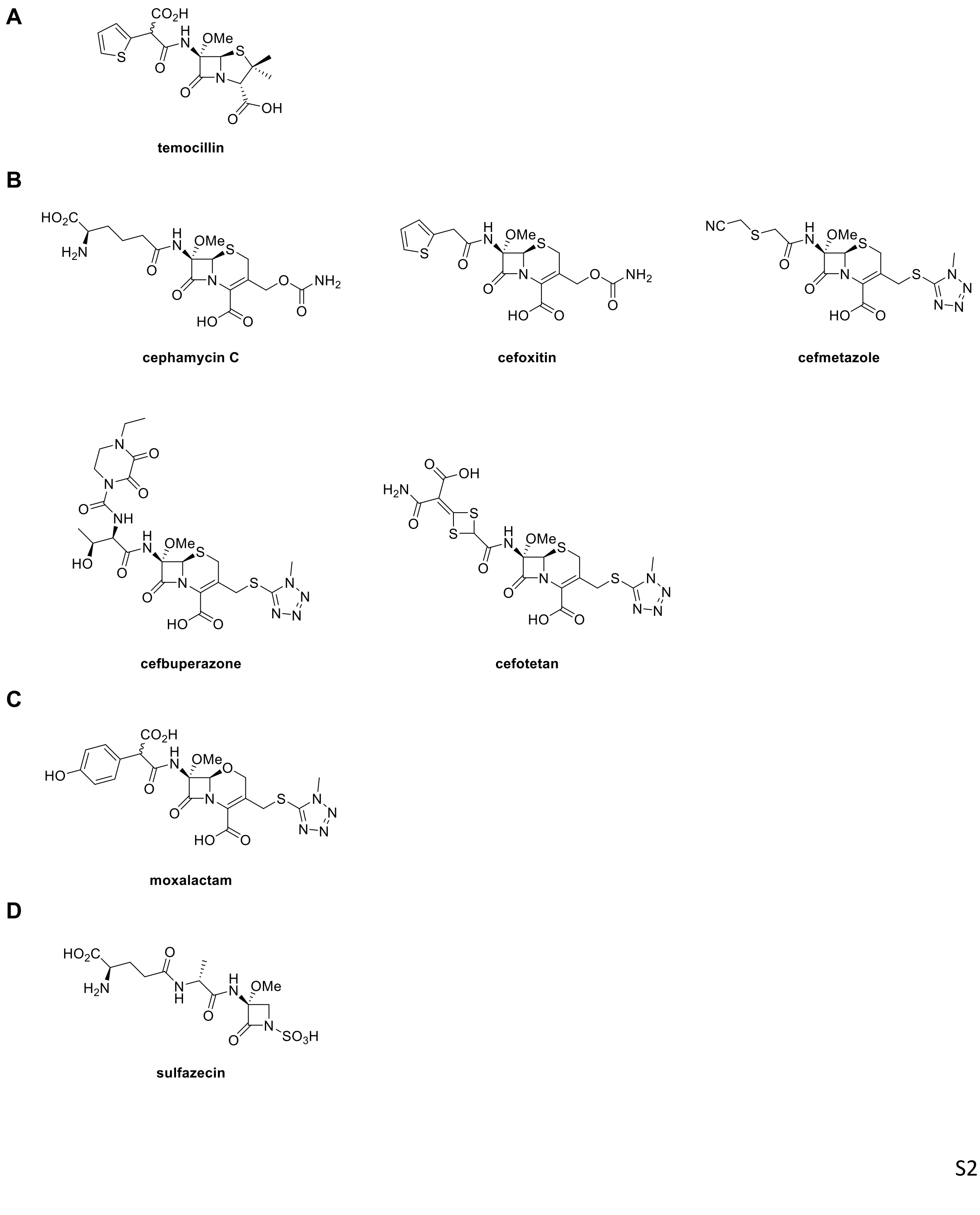

Supporting Figure S2. Comparison of predicted M^{pro}:penicillin derivatives complex structures with reported M^{pro} complex structures. A. Superimposition of the MD-predicted M^{pro}:penicillin V derivative **1** (pink) complex and the crystallographically observed covalent M^{pro}:nirmatrelvir (cyan blue) adduct (PDB ID: **7VH8**[9]). **B.** Superimposition of the MD-predicted M^{pro}:penicillin V derivative **1** (pink) complex structure and the crystallographically observed covalent M^{pro}:penicillin V derivative **4** (blue) adduct structure (PDB ID: **7Z59**[10]). S1', S1, S2, and S4 indicate the respective M^{pro} substrate residue binding pockets.

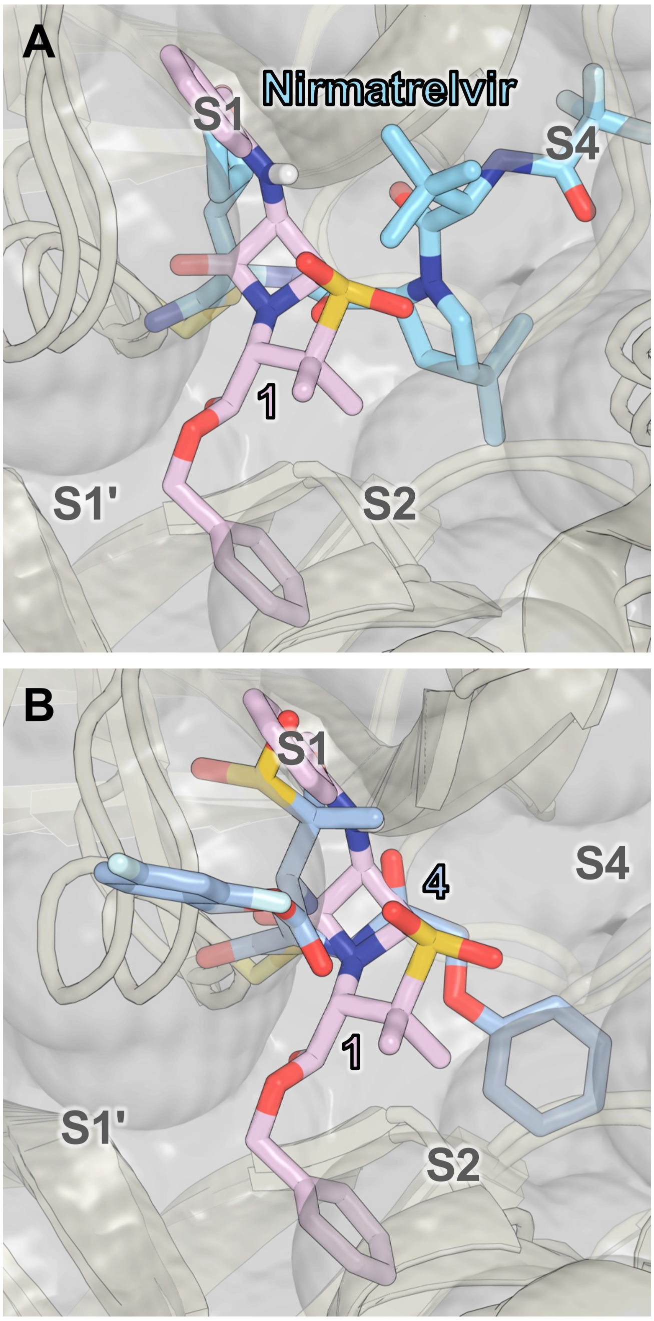

Supporting Figure S3. Comparison of predicted M^{pro}:penicillin derivative complex structures. Superimposition of the MD-predicted M^{pro}:penicillin V derivative **1** and M^{pro}:C6-methoxypenicillin G derivative **3** (purple) complex structures. S1', S1, S2, and S4 indicate the respective M^{pro} substrate residue binding pockets.

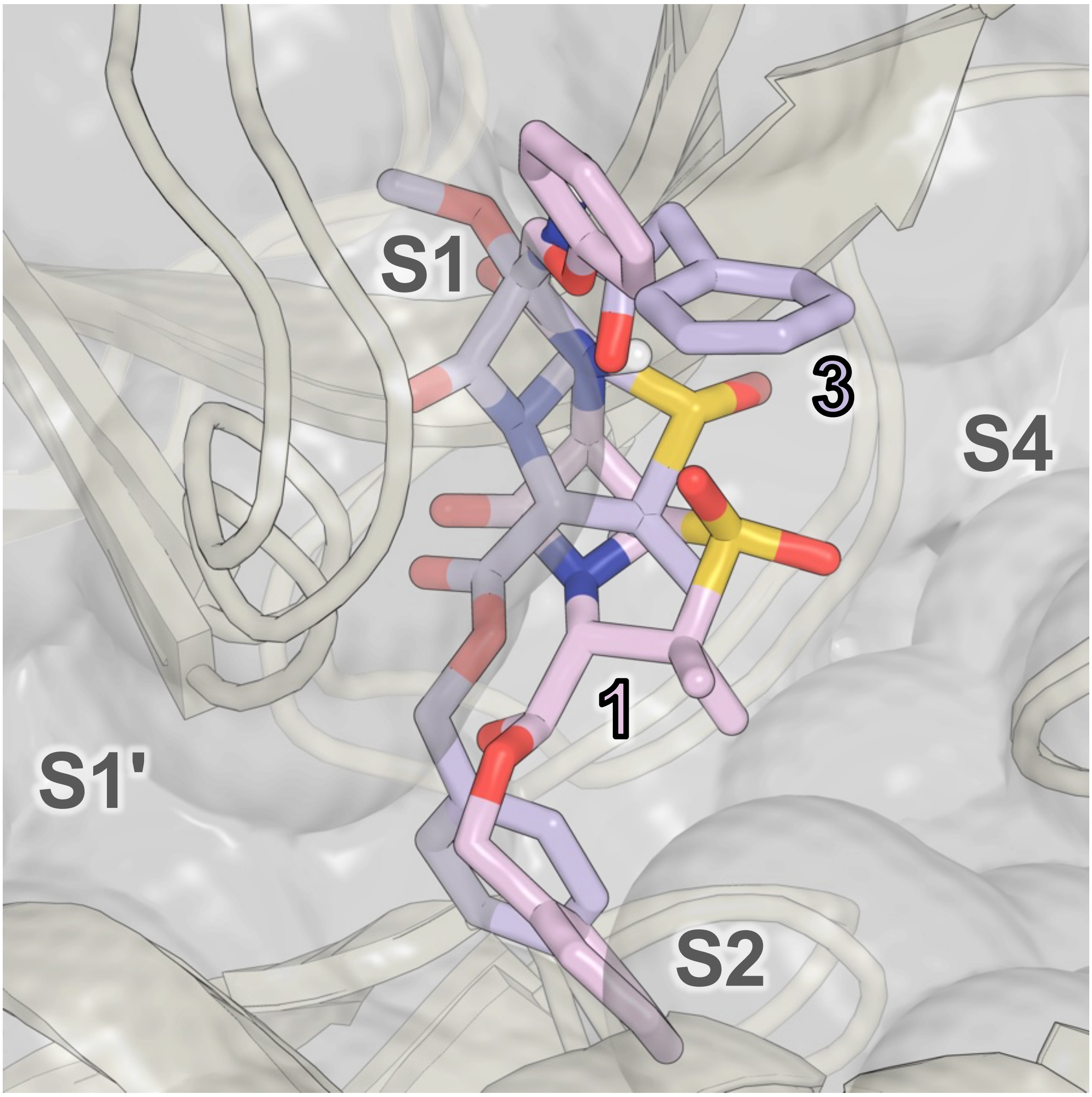

Supporting Figure S4. Comparison of predicted M^{pro}:penicillin derivative complex structures with reported M^{pro} complex structures. Superimposition of the MD-predicted M^{pro}:penicillin V derivative **10b** complex structure (green) and the crystallographically-observed covalent adduct of nirmatrelvir (cyan blue) with M^{pro} (PDB ID: **7VH8**[10]). S1', S1, S2, and S4 indicate the respective M^{pro} substrate residue binding pockets.

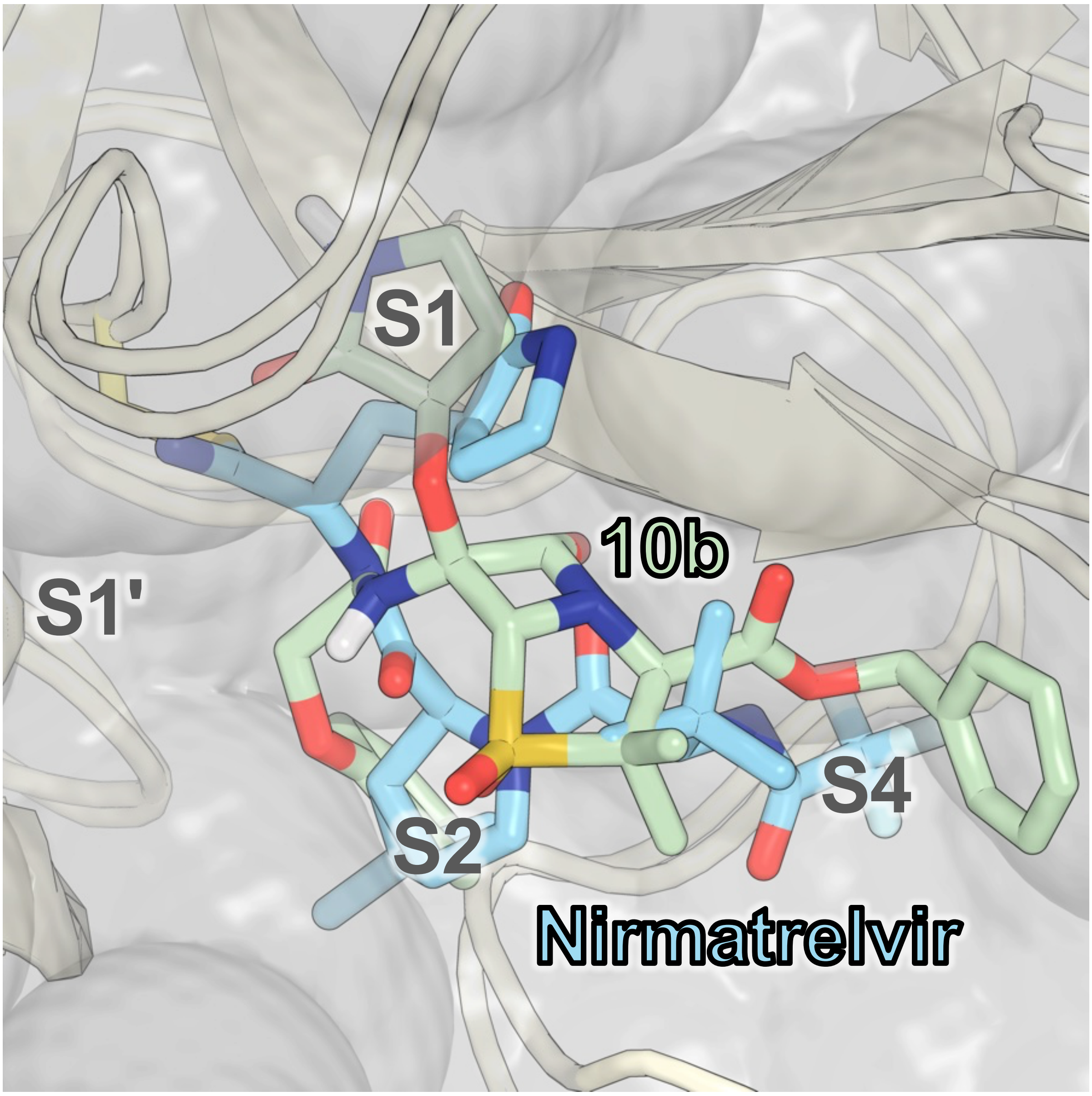

Supporting Figure S5. Molecular dynamics analysis predict that β-lactam 1 may be better poised than β-lactam 3 for covalent reaction with M^{pro} via β-lactam ring opening. Ligand root-mean-square deviation (RMSD) values relative to the representative structure from the largest observed cluster, and distances between the sulfur of Cys145 and the carbonyl carbon of the β-lactam ring (S(Cys145)-C(O)(β-lactam)) and Bürgi-Dunitz angles for S(Cys145) and the C(O)(β-lactam) for: (**A**) β-lactam **1** and (**B**) β-lactam **3**, over three independent replicates of 100 ns MD simulations. Only frames in which the S–C distance was less than 5 Å were included in the angle analyses (59% and 19% of frames for **1** and **3**, respectively). The RMSD was used to assess the stability of the ligand pose.

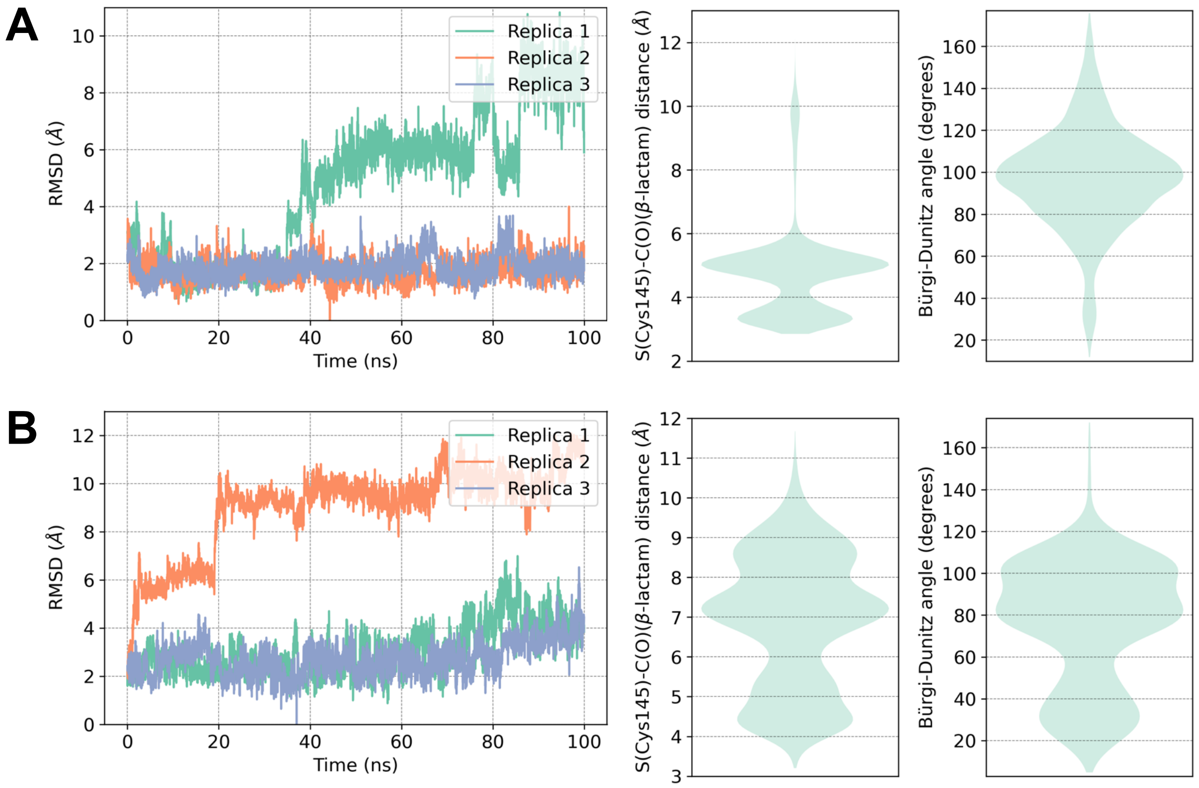

Supporting Figure S6. Modelled C6-alkoxy penicillin V derivative structures show a lack of evidence for sustained proximity or geometry favouring covalent reaction with M^{pro} Cys145. Ligand RMSD values relative to the representative structure from the largest observed cluster, as well as S(Cys145)-C(O)(β-lactam) distances and Bürgi-Dunitz angles for S(Cys145) and the C(O)(β-lactam) for: **7** (**A**), **10a** (**B**), **17** (**C**), and **10b** (**D**), over three independent replicates of 100 ns MD simulations. Only frames in which the S–C distance was less than 5 Å were included in the angle analyses (2%, 49%, < 1%, and 3% of frames for **7**, **10a**, **17**, and **10b**, respectively). The RMSD was used to assess the stability of the ligand pose.

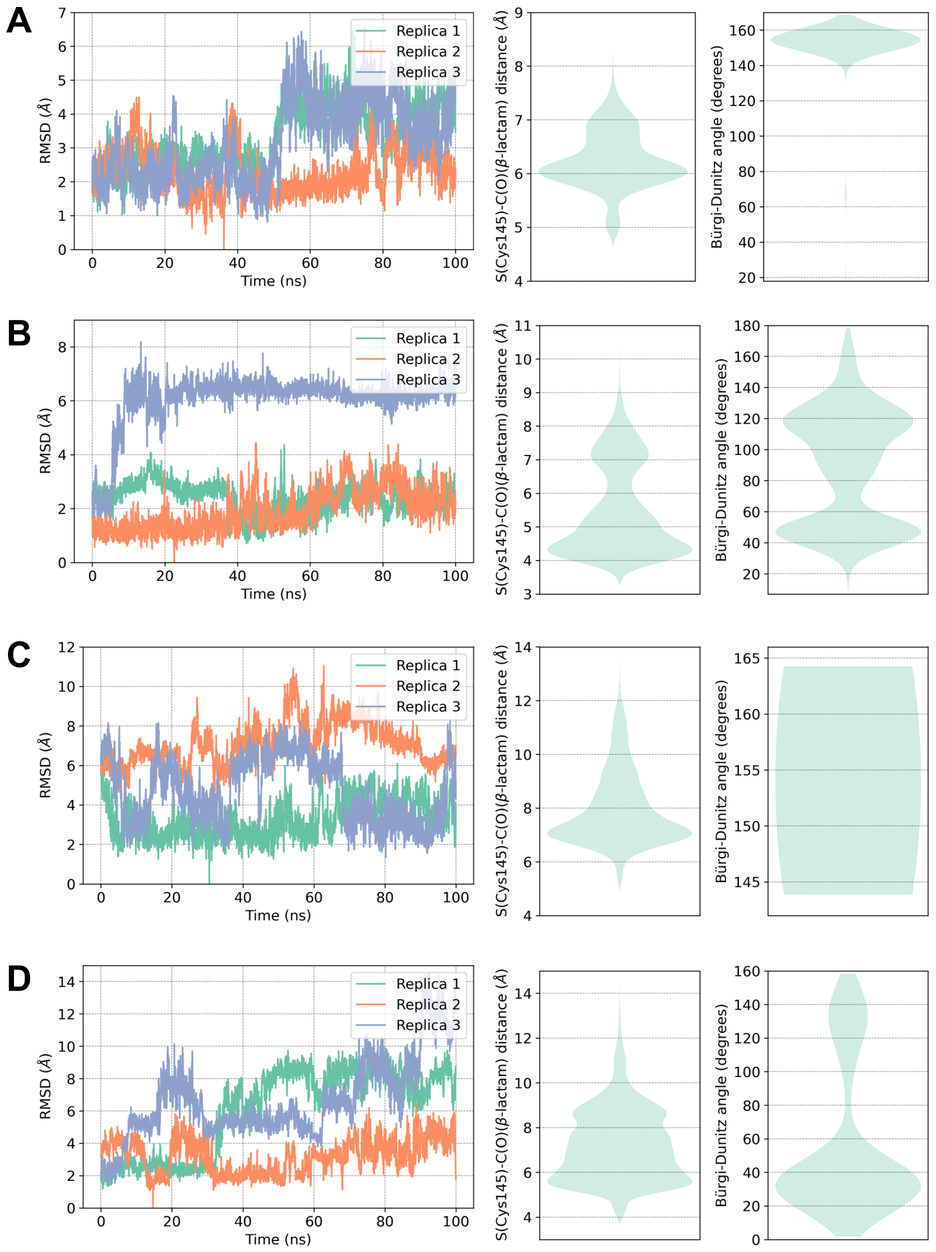

Supporting Figure S7. Modelled C6-methoxy penam derivative structures show a lack of evidence for sustained proximity or geometry favouring covalent reaction with M^{pro} Cys145. Ligand RMSD values relative to the representative structure from the largest observed cluster, as well as S(Cys145)-C(O)(β-lactam) distances and Bürgi-Dunitz angles for S(Cys145) and the C(O)(β-lactam) for **27a** (**A**) and **26a** (**B**) over three independent replicates of 100 ns MD simulations. Only frames in which the S–C distance was less than 5 Å were included in the angle analyses (53% and 2% of frames for **27a** and **26a**, respectively). The RMSD was used to assess the stability of the ligand pose. Note that **27a** was observed to dissociate from M^{pro} in replicate 2.

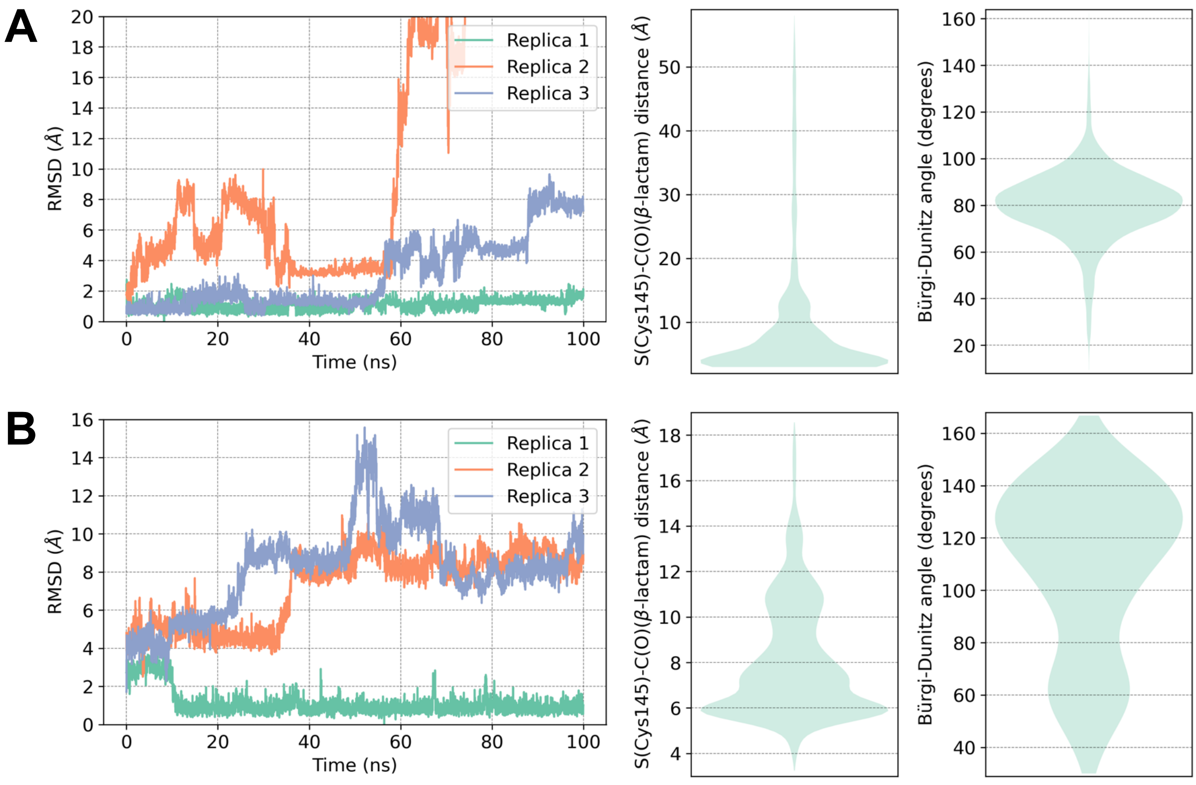

Supporting Figure S8. Representative dose response curves. Representative dose response curves for M^pro^ inhibition by C6-alkoxy penicillin: (**a**) **2** (black triangles), **3** (orange circles), **7** (blue squares), **10a** (green diamonds), **10b** (red inverse triangles), **17** (mustard green hexagons), and (**b**) **20** (black triangles), **22** (orange circles), **25** (blue squares), **26a** (green diamonds), **26b** (red inverse triangles), **26f** (mustard green hexagons), **27** (purple dots). Dose response curves are means of technical duplicates. IC_{50} values in **Tables 1-3** are means of three independent repeats (n = 3, mean ± SD), each composed of technical duplicates. Conditions: SPE-MS M^{pro} inhibition assays were performed using SPE-MS as reported,[11] employing SARS-CoV-2 M^{pro} (0.05 µM) and substrate (2.0 µM).

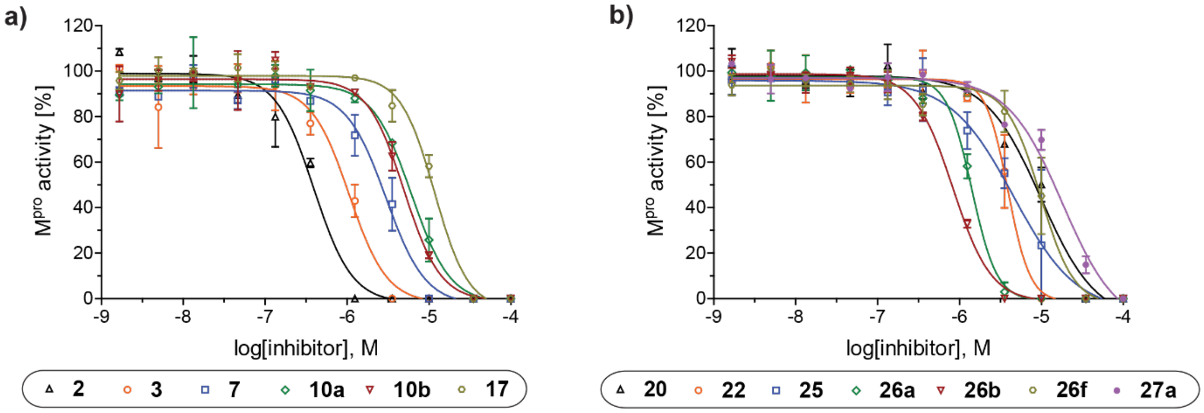

Supporting Figure S9. Most C6-alkoxy penam derivatives do not efficiently covalently react with isolated recombinant M^{pro} (continues on the following page). Analysis of reaction mixtures of M^{pro} and β-lactams: **(A) 8**, **(B) 10a**, **(C) 10b**, **(D) 16c**, **(E) 20**, **(F) 22**, **(G) 24**, **(H) 26b**, **(I) 26d**, **(J) 26e**, **(K) 26f**, **(L) 27c**, **(M) 27d**, **(N) 27e**, and **(O) 27f**, prior (bottom) and >15 h post incubation (top) with the respective β-lactam. Assays were performed using SPE-MS, employing SARS-CoV-2 M^{pro} (3.0 μM) and, if appropriate, a β-lactam (15 μM) in buffer (20 mM HEPES, pH 7.5). Representative spectra of technical duplicates are shown.

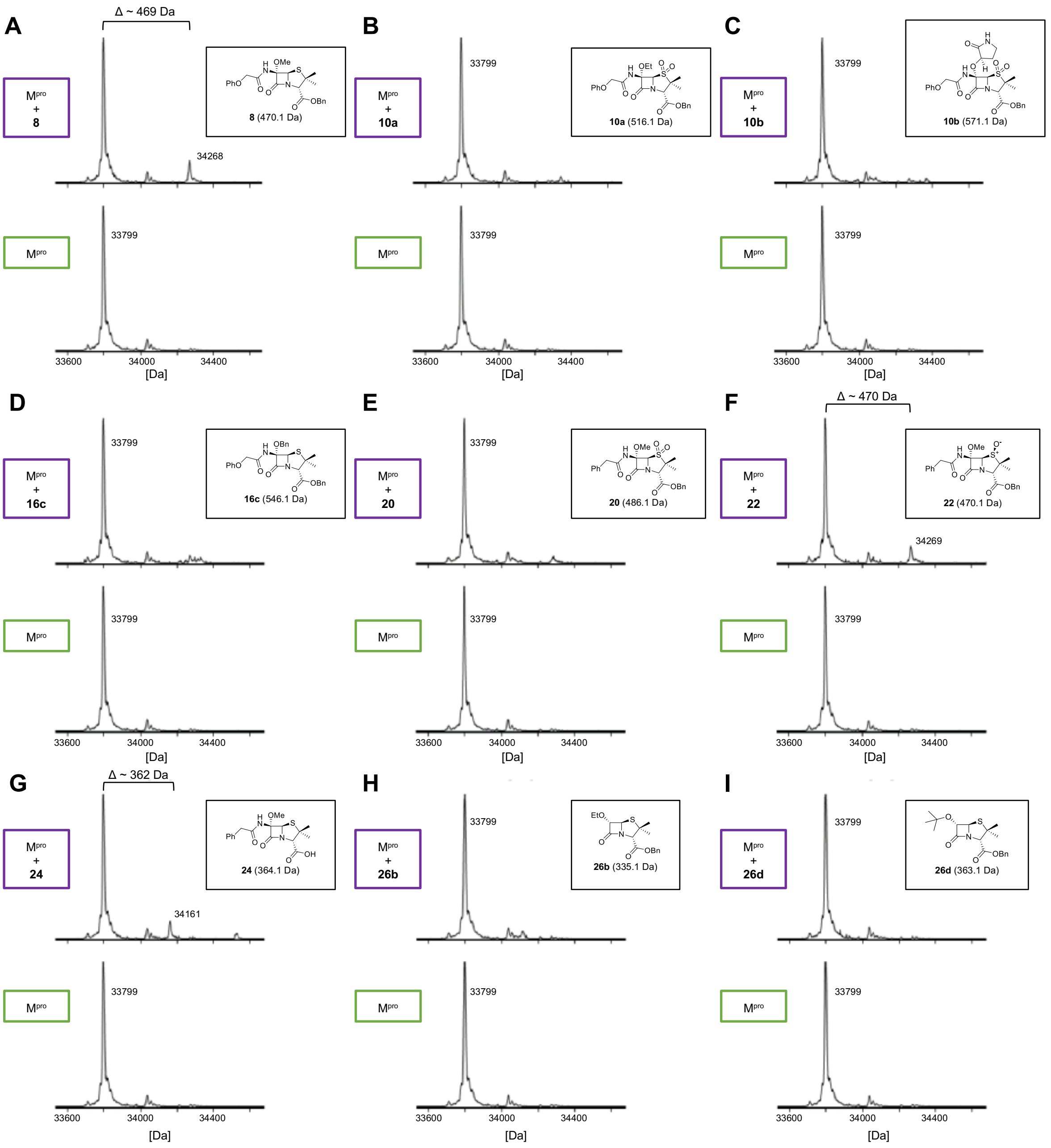

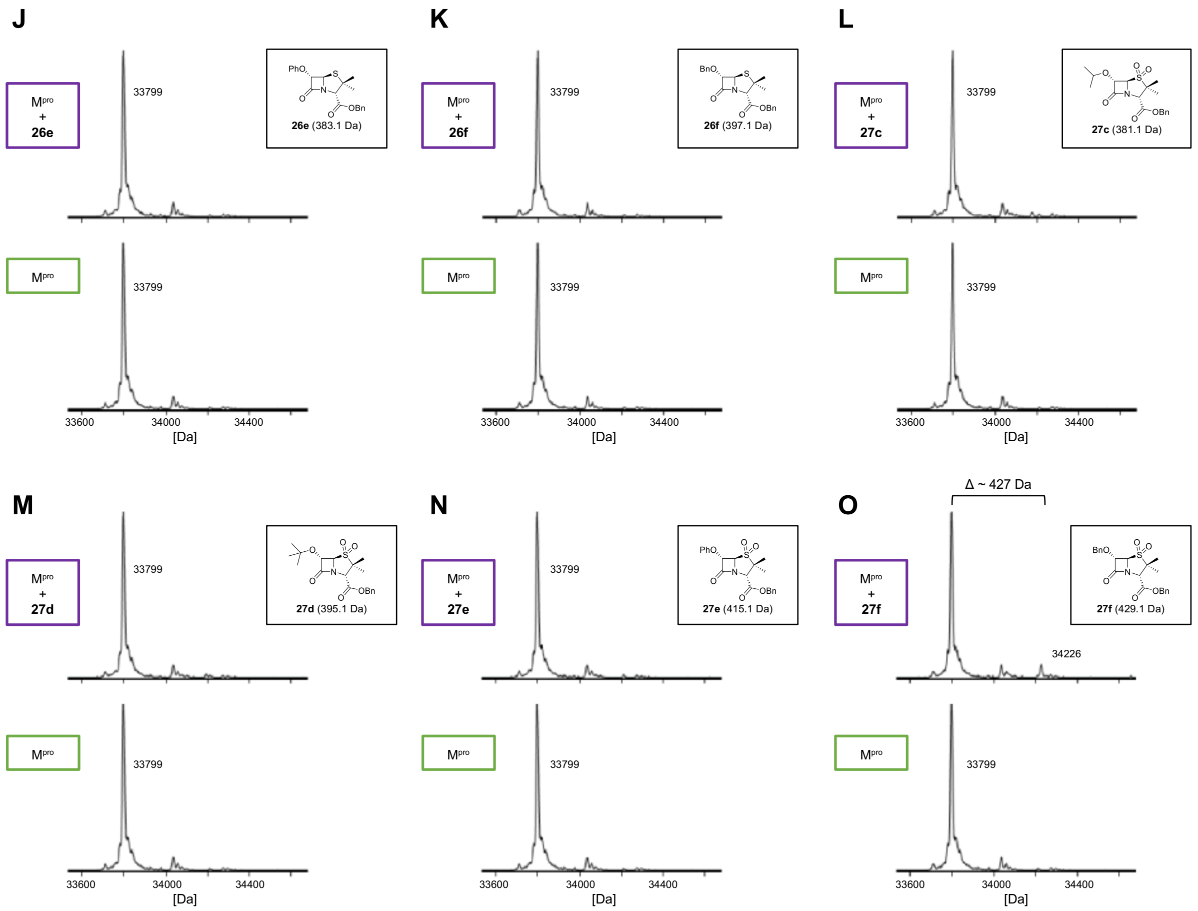
J
M^{pro} + 26e
33799
26e (383.1 Da)
M^{pro}
33799
33600
34000
34400
[Da]
K
M^{pro} + 26f
33799
26f (397.1 Da)
M^{pro}
33799
33600
34000
34400
[Da]
L
M^{pro} + 27c
33799
27c (381.1 Da)
M^{pro}
33799
33600
34000
34400
[Da]
M
M^{pro} + 27d
33799
27d (395.1 Da)
M^{pro}
33799
33600
34000
34400
[Da]
N
M^{pro} + 27e
33799
27e (415.1 Da)
M^{pro}
33799
33600
34000
34400
[Da]
O
Δ ~ 427 Da
M^{pro} + 27f
33799
34226
27f (429.1 Da)
M^{pro}
33799
33600
34000
34400
[Da]

Supporting Figure S10. Levels of M^{pro} adduct formation depend on β-lactam concentration. SPE-MS analysis indicates that an increased penicillin derivative:M^{pro} ratio correlates with higher intensity of the +32 Da M^{pro} mass shift (annotated with ★) observed with both **2** (**A**) and **3** (**B**) (denaturing conditions; 20 mM HEPES, pH 7.5). Penicillin derivative:M^{pro} ratios were: 1:5 (top spectra) or 1:10 (bottom spectra). Bn: –CH_2Ph; pNB: –$CH_2C_6H_4$(4-NO_2).

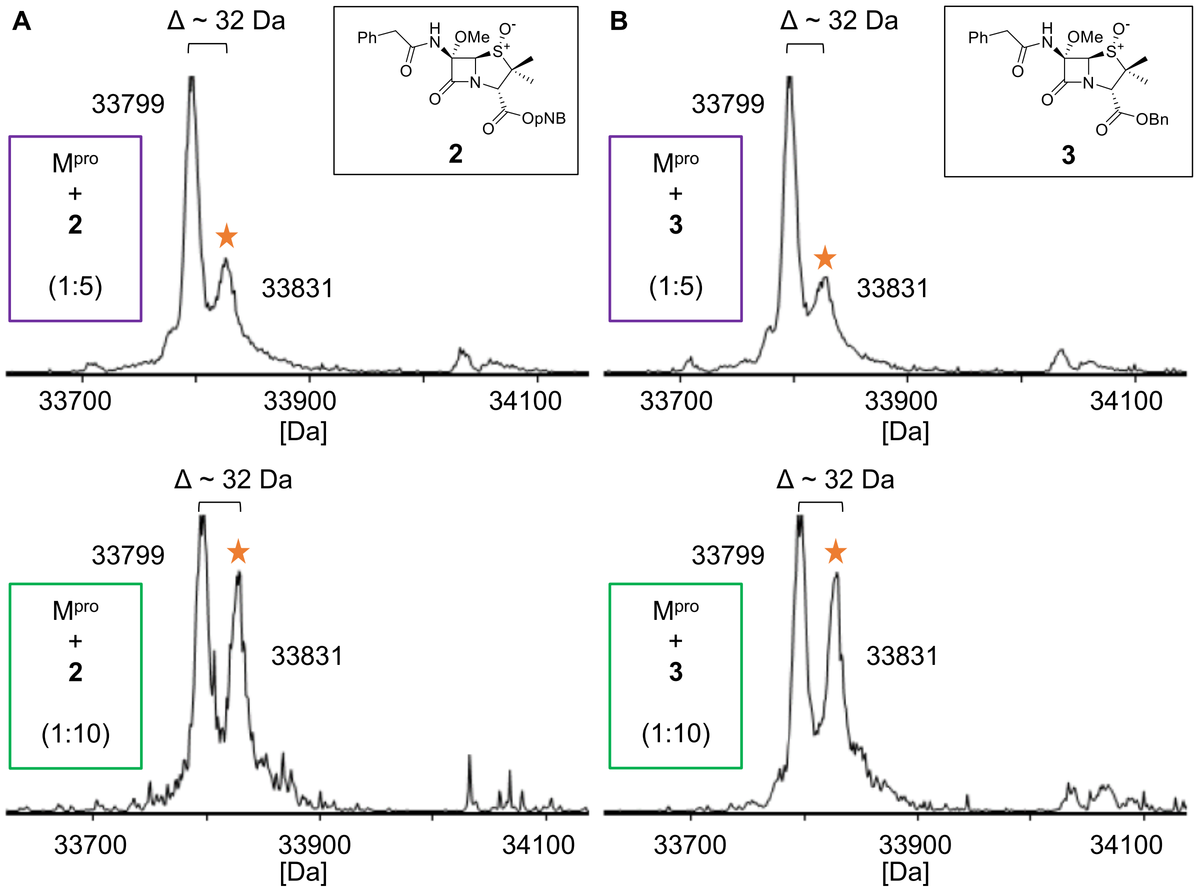

Supporting Figure S11: The outcome of the covalent reaction between M^{pro} and C6-methoxy penicillin derivative 2 is time dependent. SPE-MS analysis showed initial formation of a +515 Da M^{pro} adduct (★), followed by the appearance of a +498 Da M^{pro} adduct (★) with increasing intensity over 45 min (denaturing conditions; **2**:M^{pro} ratio: 10:1; 20 mM HEPES, pH 7.5). A +32 Da M^{pro} adduct (★) of low intensity was also detected after 45 min incubation. pNB: –$CH_2C_6H_4$(4-NO_2).

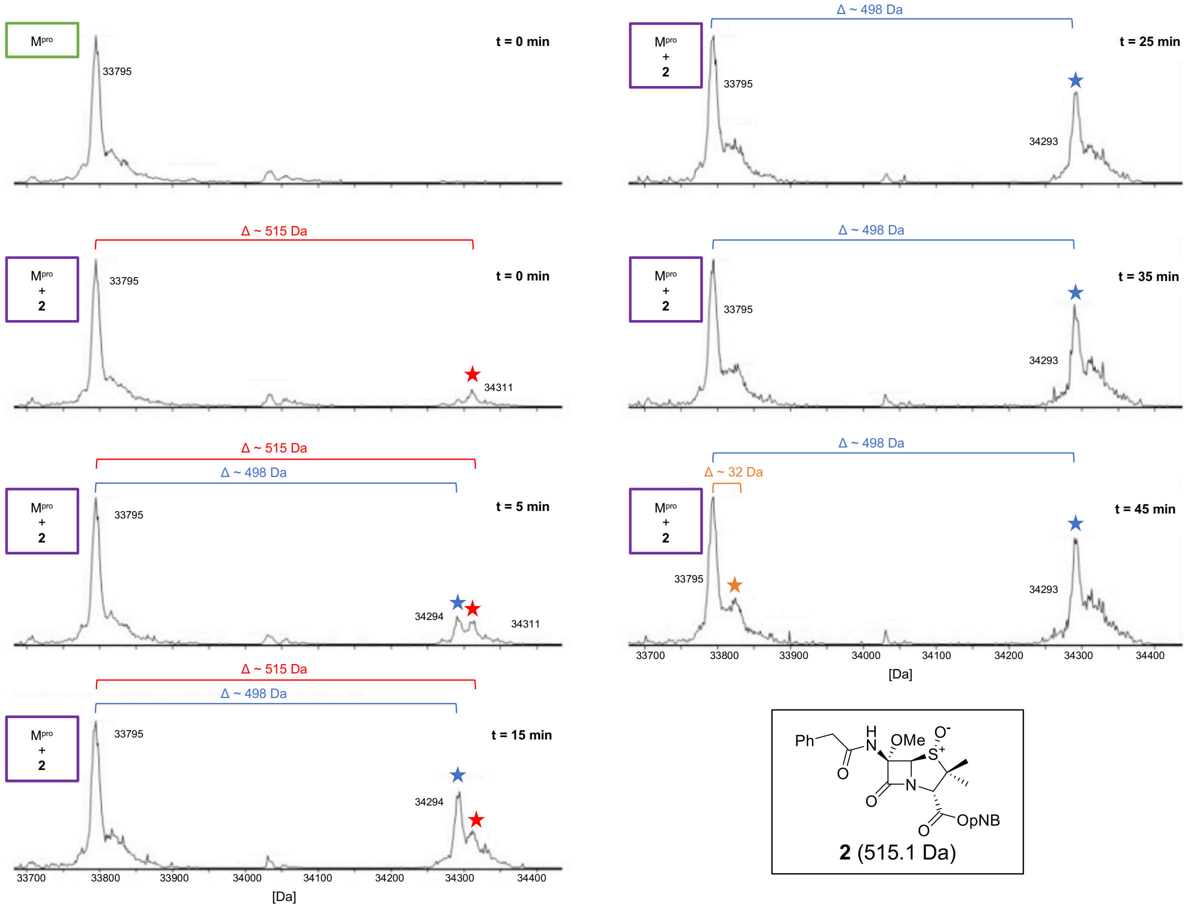

Supporting Figure S12. β-Lactams have potential for protein inhibition via non-covalent mechanisms. Structures of reported β-lactams that inhibit proteins via non-covalent binding: (**A**) Ezetimibe[12] is an anticholesteremic drug that competitively inhibits the NPC1L1 transporter. (**B**) Penicillin A11 and cephalosporin E1 are likely non-covalent inhibitors of SPOP (reported IC_{50} ~ 20 and 0.58 μM, respectively).[13]

A **B**

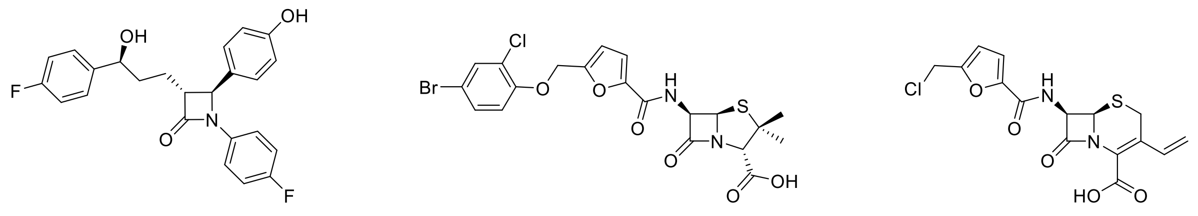

Ezetimibe **A11** **E1**

Supporting Figure S13. Possible outline mechanism for covalent reaction of M^pro^ with C6-methoxy penicillin G (*R*)-sulfoxide esters. Reaction of M^{pro} with **2** may involve: a) active-site mediated deprotonation of the relatively acidic C3 proton of **2**, giving sulfenic acid (**I**); b) S-S bond formation via reaction with a M^{pro} cysteine thiol (+498 Da; **II**); c) elimination of the M^{pro} persulfide **II** (+32 Da; see **Figure 8** and **Supporting Figure S11** for relevant MS details), likely followed by: d) hydration of the intermediate β-lactam **IV**. pNB: $–CH_2C_6H_4(4\text{-}NO_2)$.

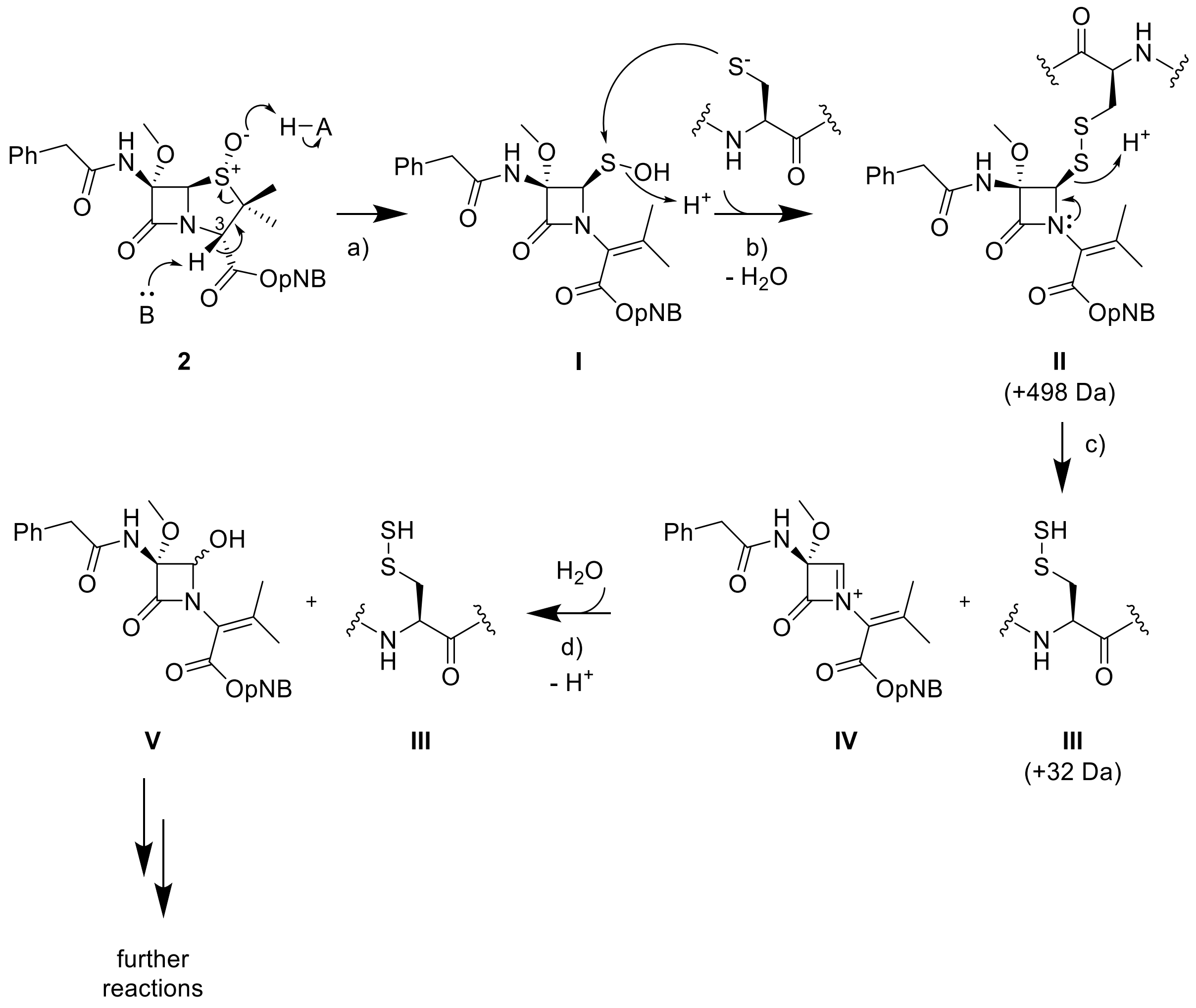

2. Computational methods

2.1 Docking

Docking was performed using an *apo* SARS-CoV-2 M^{pro} crystal structure (PDB ID 6YB7, 1.25 Å resolution). Small-molecule input conformations were generated using Schrödinger's LigPrep tool, the protein was prepared using Schrödinger's Protein Preparation Workflow,[14, 15] both with default settings. Solvent molecules were removed. δ-Nitrogens of His41 and His80 were protonated. ε-Nitrogens of His64, His163, His164, His172, and His246 were protonated.

Docking calculations were performed using Glide XP[16] with a bounding box (inner and outer edge length: 10 Å and 30 Å, respectively) centred on the Cys145 sulphur atom and a flexible Cys145 thiol group, keeping at most 30 poses per ligand. Output poses were clustered based on interaction fingerprints using default settings. Structures closest to the centroid of each cluster were rescored using molecular mechanics with generalised Born and surface area continuum solvation (MM-GBSA) calculations, minimising all protein atoms within 5 Å of the ligand, and the top three structures were kept for further analysis. For penams **1**, **7**, and **10b** cluster representatives were highly similar (RMSD < 3 Å), and thus only cluster the representative with the lowest MM-GBSA score was kept. MM-GBSA scores substantially altered the ranking of cluster representatives; in some cases, the lowest-ranking pose according to docking scores was ranked the highest according to MM-GBSA scores.

2.2 Parametrisation

Parameters for the inhibitors were generated using Antechamber[17, 18]. Partial charges were computed using the optimised geometry of the highest-ranking conformer using the Restrained Electrostatic Potential (RESP) procedure.[19] QM calculations used to derive ESP were performed at the HF/6-31G(d) level of theory using the Gaussian16 software package[20].

Docked protein-ligand complexes were prepared using LEaP[21, 22]. The ff14SB[24] force field was used for the protein and GAFF[17, 18] for the small molecules.

2.3 Molecular dynamics simulations

The protein-ligand complexes obtained from docking were used as starting points for MD simulations. The system was placed in a truncated octahedral box with a 12 Å buffer, solvated with the TIP3P water model[23], and neutralised with 4 sodium ions. To ensure the planarity of the β-lactam carbonyl, the barrier height for the corresponding improper torsion was set to 10.5 kcal/mol.

Molecular dynamics (MD) simulations were performed using Amber24[21]. The system was subjected to energy minimisation in two stages – first with positional restraints on protein and ligand atoms at 500 kcal/mol $Å^{-2}$, followed by unrestrained minimisation. The system was then heated from 0 to 300 K in 50 K increments under constant volume using Langevin dynamics with a time step of 2 fs, gradually releasing positional restraints on the protein and ligand (starting at 210 kcal/mol $Å^{-2}$ and decreasing to 10 kcal/mol $Å^{-2}$). Equilibration was performed for 2 ns using a 2 fs time step in the NPT ensemble at 1 bar and 300 K. Temperature and pressure were maintained using a Langevin thermostat (collision frequency: 1.0 ps^{-1}) and a Berendsen barostat (relaxation time: 2.0 ps)[24], respectively.

Production MD simulations were conducted for 100 ns using the NPT ensemble at 1 bar and 300 K. A 10 Å cut-off was applied for non-bonded interactions. Covalent bonds involving hydrogen atoms were constrained using the SHAKE algorithm[25], and periodic boundary conditions were employed with particle mesh Ewald (PME)[26] for long-range electrostatics. Three independent replicates of minimisation, heating, and equilibration, and production simulations were performed.

CPPTRAJ[21, 27] was used to analyse trajectories. The pose stability was determined by ligand root-mean-square deviation (RMSD) compared to the initial, docked pose after aligning the protein backbone, and validated by visual inspection. To identify representative ligand binding modes, the trajectories from the independent replicates were concatenated and aligned to the protein backbone atoms. Water molecules and ions were removed, and hierarchical agglomerative clustering based on ligand heavy atom RMSD was performed using the average linkage method with an epsilon cut-off of 1.0 Å. Cluster populations and representative structures were extracted, with the largest cluster considered to represent the most probable binding mode. H bonds were required to have a donor-acceptor distance < 3.0 Å and an angle between the H bond donor, the H atom, and the H bond acceptor > 135°. The fraction of simulation time that each hydrogen bond was observed is given in **Supporting Table S1**. A H bond was considered relevant if it was present for at least 10% of the simulation time. Visualisations were generated using PyMOL. Asn142 was excluded from the protein surface for visual clarity. Simulation input files, scripts, and final poses are available in a GitHub repository (https://github.com/duartegroup/C6-alkoxy-penicillins).

Supporting Table S1: Fractions of simulation time across the concatenated three independent replicates that the indicated hydrogen bonds were present.

Hydrogen bond	Fraction of simulation time
**1 : His163 sidechain**	22%
**1 sulfone O : 1 amide NH**	10%
**3 : Ser144 backbone**	11%
**3 : Glu166 backbone**	33%
**3, Glu166 sidechain**	20%/15% (for each sidechain O)
**7 : Asn142 sidechain**	40%
**7 : Gly143 backbone**	35%
**7 : Gln189 sidechain**	12%
**10a : His41 sidechain**	22%
**10a : sulfone O : 10a amide NH**	11%
**10b : Leu141 backbone**	13%
**10b : Gly143 backbone**	18%
**10b : Ser144 sidechain**	15%
**10b : Glu166 backbone**	13%
**27a : Gly143 backbone**	28%

3. General synthesis information

All reagents were obtained from commercial sources (Sigma-Aldrich, Inc.; Flurochem Ltd.; Manchester Organics; Tokyo Chemical Industry Ltd.) and were used without further purification. Benzyl (2*S*,5*R*,6*R*)-3,3-dimethyl-7-oxo-6-(2-phenoxyacetamido)-4-thia-1-azabicyclo[3.2.0]heptane-2-carboxylate 4,4-dioxide (**1**)[10], 4-nitrobenzyl (2*S*,4*R*,5*R*,6*S*)-6-methoxy-3,3-dimethyl-7-oxo-6-(2-phenylacetamido)-4-thia-1-azabicyclo[3.2.0]heptane-2-carboxylate 4-oxide (**2**)[28], benzyl (2*S*,5*R*,6*R*)-3,3-dimethyl-7-oxo-6-(2-phenoxyacetamido)-4-thia-1-azabicyclo[3.2.0]heptane-2-carboxylate (**5**)[10], benzyl (2*S*,4*S*,5*R*,6*R*)-3,3-dimethyl-7-oxo-6-(2-phenoxyacetamido)-4-thia-1-azabicyclo[3.2.0]heptane-2-carboxylate 4-oxide (**6**)[10], benzyl (2*S*,5*R*,6*R*)-6-amino-3,3-dimethyl-7-oxo-4-thia-1-azabicyclo[3.2.0]heptane-2-carboxylate toluene-*p*-sulfonic acid salt (**11**)[10], benzyl (2*S*,5*R*,6*R*)-6-(((*E*)-2,6-di-tert-butyl-4-hydroxybenzylidene)amino)-3,3-dimethyl-7-oxo-4-thia-1-azabicyclo[3.2.0]heptane-2-carboxylate (**12**)[29], benzyl (2*S*,5*R*,6*R*)-3,3-dimethyl-7-oxo-6-(2-phenylacetamido)-4-thia-1-azabicyclo[3.2.0]heptane-2-carboxylate (**18**)[30], benzyl (2*S*,5*R*,6*R*)-3,3-dimethyl-7-oxo-6-(2-phenylacetamido)-4-thia-1-azabicyclo[3.2.0]heptane-2-carboxylate 4,4-dioxide (**19**)[10], and benzyl (2*S*,4*S*,5*R*,6*R*)-3,3-dimethyl-7-oxo-6-(2-phenylacetamido)-4-thia-1-azabicyclo[3.2.0]heptane-2-carboxylate 4-oxide (**21**)[30] were synthesized as reported.

Anhydrous solvents (Sigma-Aldrich, Inc.) were kept under an atmosphere of argon. HPLC grade solvents (Sigma-Aldrich Inc.) were used for reaction work-ups, extractions, and purifications. Flash column chromatography was performed using an automated Biotage Isolera One purification machine equipped with Biotage®Sfär flash chromatography cartridges. ^{1}H and ^{13}C NMR spectra were recorded using Bruker AVANCE AVIIIHD 400, 500 and 600 MHz machines. Chemical shifts for protons are reported in parts per million (ppm) downfield from tetramethylsilane and are referenced to residual protium in the NMR solvent (*i.e.*, $CDCl_3$:

δ = 7.26 ppm; DMSO-d_6: δ = 2.50 ppm). For ^{13}C NMR, chemical shifts are reported in the scale relative to the NMR solvent (*i.e.*, $CDCl_3$: δ = 77.2 ppm; DMSO-d_6: δ = 39.5 ppm). NMR data are reported in the following manner: chemical shift, multiplicity, coupling constant (J, Hz; to nearest 0.1 Hz), and integration.

Infrared (IR) spectroscopy was performed using a Bruker Tensor-27 Fourier transform infrared (FT-IR) spectrometer. High-resolution mass spectrometry (HRMS) was performed using either electro-spray ionization (ESI) mass spectrometry (MS) in the positive or negative ionization modes employing a Thermo Scientific Exactive mass spectrometer (ThermoFisher Scientific), or atmospheric pressure chemical ionization (APCI) in the positive mode using an Agilent 7200 Accurate mass spectrometer; data are presented as a mass-to-charge ratio (m/z). Optical rotation ($[\alpha]_D^{25}$) measurements were performed using either a Unipol (Schmidt Haensch) or Bellingham + Stanley (ADP450) polarimeter.

4. General synthetic procedures

General procedure A:

A variation of a reported procedure was followed.[31] To a stirred solution of a penam in anhydrous CH_2Cl_2 (1 equiv.; 0.1-0.2 M) was added freshly prepared ^{t}BuOCl[32] (1.5 equiv.), followed by a solution of LiOMe (1.1 equiv.) in MeOH (1 M) at -30 °C. The reaction mixture was stirred under nitrogen atmosphere for 1 h at -30 °C, before the addition of AcOH (1 equiv.). The mixture was diluted with CH_2Cl_2 and was sequentially washed with saturated aqueous $NaHCO_3$ solution, aqueous $Na_2S_2O_3$ (5% (w/v)) solution, and brine. The organic layer was dried over Na_2SO_4, filtered, concentrated *in vacuo*, and purified by flash column chromatography.

General procedure B:

A variation of a reported procedure was followed.[33] To a stirred solution of a penam in CH_2Cl_2 (1 equiv.; 0.16 M) was added an ice-cold aqueous solution of $NaNO_2$ (2.6 equiv.; 0.9 M), followed by addition of *p*-toluenesulfonic acid monohydrate (0.3 equiv.). After stirring the reaction mixture for 20 min at 0 °C, the organic layer was separated, and the aqueous phase was then extracted with CH_2Cl_2. The combined extracts were sequentially washed with saturated aqueous $NaHCO_3$, H_2O, then dried over Na_2SO_4, filtered, and concentrated *in vacuo* at 30 °C to give crude benzyl (2*S*,5*R*)-6-diazo-3,3-dimethyl-7-oxo-4-thia-1-azabicyclo[3.2.0]heptane-2-carboxylate[34]. The crude residue was dissolved in anhydrous CH_2Cl_2 (0.2 M), to which an anhydrous alcohol (1-110 equiv.) and boron trifluoride diethyl etherate (0.1 equiv.) were added sequentially. The mixture was stirred at 20 °C for 15 min, before being diluted with CH_2Cl_2. The mixture was washed with saturated aqueous $NaHCO_3$ solution and H_2O. The organic layer was dried over Na_2SO_4, filtered, concentrated *in vacuo*, and purified by flash column chromatography.

General procedure C:

A variation of a reported procedure was followed.[10] To a stirred solution of a penam in anhydrous CH_2Cl_2 (1 equiv.; 0.1 M) was added *m*-chloroperbenzoic acid (<77% purity; 2.3 equiv.) in portions over 5 min at 0 °C. The reaction mixture was stirred for 1 h at 10 °C, then at room temperature overnight (16-20 h), before being diluted with CH_2Cl_2. The mixture was sequentially washed with aqueous $Na_2S_2O_5$ (10% (w/v)) solution, saturated aqueous $NaHCO_3$ solution, water, and brine. The organic layer was dried over Na_2SO_4, filtered, concentrated *in vacuo*, and purified by flash column chromatography.

5. Experimental procedures and compound characterisations

Benzyl (2*S*,5*R*,6*S*)-6-methoxy-3,3-dimethyl-7-oxo-6-(2-phenoxyacetamido)-4-thia-1-azabicyclo[3.2.0]heptane-2-carboxylate 4-oxide (7)

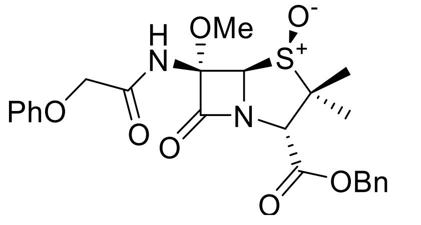

General procedure A[31] was used with penam **6** (1.17 g, 2.56 mmol). Purification by flash column chromatography (25 g Sfär: 100%$_{v/v}$ cyclohexane (2 CV), then a linear gradient (9 CV): 0%$_{v/v}$ →11%$_{v/v}$ ethyl acetate in cyclohexane, then 11%$_{v/v}$ ethyl acetate in cyclohexane (2 CV), then a linear gradient (14 CV): 11%$_{v/v}$ →28%$_{v/v}$ ethyl acetate in cyclohexane, then 28%$_{v/v}$ ethyl acetate in cyclohexane (2 CV), then a linear gradient (18 CV): 28%$_{v/v}$ →60%$_{v/v}$ ethyl acetate in cyclohexane) yielded **7** (570 mg, 45%).

Amorphous foam; ^{1}H NMR (500 MHz, 298 K, $CDCl_3$): δ = 7.53 (s, 1H), 7.40 – 7.36 (m, 5H), 7.32 – 7.28 (m, 2H), 7.03 – 6.99 (m, 1H), 6.96 – 6.92 (m, 2H), 5.32 and 5.19 (ABq, J = 12.0 Hz, 2H), 4.99 (s, 1H), 4.62 – 4.50 (m, 3H), 3.49 (s, 3H), 1.58 (s, 3H), 1.08 ppm (s, 3H); ^{13}C NMR (126 MHz, 298 K, $CDCl_3$): δ = 170.3, 167.6, 166.8, 157.1, 134.8, 129.9, 129.05, 128.98, 128.9, 122.5, 115.0, 90.8, 79.4, 77.4, 71.7, 68.1, 67.2, 64.2, 53.5, 19.4, 18.5 ppm; IR (film): ṽ = 1792, 1753, 1696, 1599, 1496, 1438, 1215, 1172, 1060 cm^{-1}; HRMS (ESI): m/z calculated for $C_{24}H_{26}N_2O_7S$ $[M+Na]^+$: 509.1353, found: 509.1350; $[\alpha]_D^{25}$ = +224 (c =0.006 g/mL, $CHCl_3$).

Benzyl (2*S*,5*R*,6*S*)-6-methoxy-3,3-dimethyl-7-oxo-6-(2-phenoxyacetamido)-4-thia-1-azabicyclo[3.2.0]heptane-2-carboxylate (8)[35]

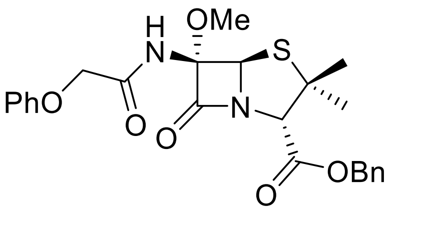

To a solution of **7** (300 mg, 0.615 mmol, 1 equiv.) in *N*,*N*-dimethylformamide (6.1 mL) cooled to 0 °C were sequentially added KI (1.85 g, 11.1 mmol, 18 equiv.) then CH_3COCl (0.31 mL, 4.34 mmol, 7 equiv.). The reaction

mixture was stirred for 1 h at 0 °C, then it was diluted with EtOAc (50 mL). The organic layer was sequentially washed with an aqueous solution of $Na_2S_2O_3$ (5% (w/v)), saturated aqueous $NaHCO_3$ solution, and brine, before being dried over Na_2SO_4, filtered, concentrated *in vacuo*. Purification by flash column chromatography (25 g Sfär: 100%$_{v/v}$ cyclohexane (4 CV), then a linear gradient (14 CV): 0%$_{v/v}$ →21%$_{v/v}$ ethyl acetate in cyclohexane, then 21%$_{v/v}$ ethyl acetate in cyclohexane (8 CV)) yielded **8** (290 mg, 99%). Synthesis of **8** was achieved following an alternative experimental procedure to that reported.[35] The analytical data are consistent with those reported.[35]

Amorphous foam; ^{1}H NMR (400 MHz, 298 K, DMSO-*d6*): δ = 9.64 (s, 1H), 7.45 – 7.35 (m, 5H), 7.31 – 7.26 (m, 2H), 6.98 – 6.90 (m, 3H), 5.42 (s, 1H), 5.21 (d, *J* = 1.2 Hz, 2H), 4.66 and 4.57 (ABq, *J* = 15.4 Hz, 2H), 4.58 (s, 1H), 3.38 (s, 3H), 1.45 (s, 3H), 1.31 ppm (s, 3H); ^{13}C NMR (101 MHz, 298 K, DMSO-*d6*): δ = 168.5, 167.5, 166.9, 157.7, 135.2, 129.3, 128.4 (2C), 128.3, 121.0, 114.5, 94.8, 73.7, 68.0, 66.8, 66.1, 61.7, 52.9, 31.8, 25.4 ppm; IR (film): ṽ = 1774, 1746, 1703, 1598, 1505, 1437, 1316, 1237, 1205, 1186, 1111 cm^{-1}; HRMS (ESI): m/z calculated for $C_{24}H_{26}N_2O_6S$ [M+Na]$^+$: 493.1404, found: 493.1409; $[\alpha]_D^{25}$ = +163 (c = 0.003 g/mL, $CHCl_3$).

Benzyl (2*S*,5*R*,6*S*)-6-methoxy-3,3-dimethyl-7-oxo-6-(2-phenoxyacetamido)-4-thia-1-azabicyclo[3.2.0]heptane-2-carboxylate 4,4-dioxide (9)

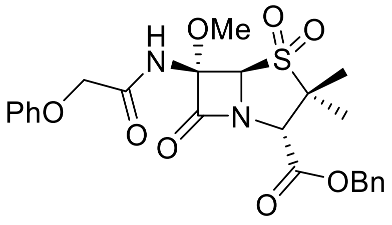

mCPBA (<77% purity; 1.5 equiv., 70 mg) was added at 0 °C to a stirred solution of **7** (100 mg, 0.205 mmol, 1 equiv.) in anhydrous $CHCl_3$ (4 mL). The reaction mixture was further stirred for another 4 h at room temperature, at which point an additional portion of mCPBA (<77% purity; 23 mg) was added. The reaction mixture was stirred at room temperature for 16 h, before it was diluted with CH_2Cl_2, then

sequentially washed with an aqueous solution of $Na_2S_2O_3$ (5% (w/v)), saturated aqueous $NaHCO_3$ solution, water, and brine. The organic layer was dried over Na_2SO_4, filtered, then concentrated *in vacuo*. Purification by flash column chromatography (10 g Sfär: 100%$_{v/v}$ cyclohexane (2 CV), then a linear gradient (36 CV): 0%$_{v/v}$ →60%$_{v/v}$ ethyl acetate in cyclohexane) yielded **9** (67 mg, 65%).

Amorphous solid; ^{1}H NMR (600 MHz, 298 K, DMSO-d_6): δ = 7.40 (m, 5H), 7.33 – 7.29 (m, 2H), 7.02 (s, 1H), 7.01 – 6.97 (m, 3H), 5.25 (s, 2H), 5.02 (s, 1H), 4.32 and 4.24 (ABq, J = 9.7 Hz, 2H), 3.71 (s, 3H), 1.52 (s, 3H), 1.31 ppm (s, 3H); ^{13}C NMR (151 MHz, 298 K, DMSO-d_6): δ = 166.1, 157.9, 155.9, 151.9, 135.0, 129.5, 128.51, 128.48, 128.4, 121.3, 115.0, 82.0, 71.6, 70.5, 67.4, 62.3, 60.3, 54.2, 20.6, 17.6, ppm; IR (film): ṽ = 1801, 1758, 1701, 1600, 1496, 1464, 1378, 1333, 1217, 1174, 1120, 1070 cm^{-1}; HRMS (ESI): m/z calculated for $C_{24}H_{26}N_2O_8S$ $[M+Na]^+$: 525.1302, found: 525.1297; $[\alpha]_D^{25}$ = +91 (c = 0.012 g/mL, $CHCl_3$).

Benzyl (2*S*,5*R*,6*S*)-6-ethoxy-3,3-dimethyl-7-oxo-6-(2-phenoxyacetamido)-4-thia-1-azabicyclo[3.2.0]heptane-2-carboxylate 4,4-dioxide (10a)

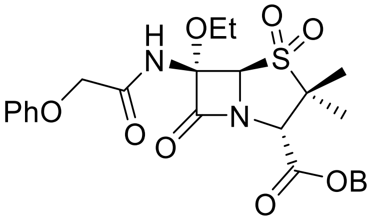

To a solution of **1** (249 mg, 0.53 mmol, 1 equiv.) in anhydrous CH_2Cl_2 (4 mL) cooled to -30 °C was added freshly prepared ^{t}BuOCl[32] (0.095 mL, 0.84 mmol, 1.6 equiv.), followed by a solution of NaOEt solution in EtOH (3M, 0.19 mL). The solution was stirred for 1 h at -30 °C, before the addition of AcOH (0.16 mL). The mixture was diluted with CH_2Cl_2, and sequentially washed with saturated aqueous $NaHCO_3$ solution, aqueous $Na_2S_2O_3$ (5% (w/v)) solution, and brine. The organic layer was dried over Na_2SO_4, filtered, then concentrated *in vacuo*. Purification by flash column chromatography (10 g Sfär: 100%$_{v/v}$ cyclohexane (2 CV), then a linear gradient (30 CV): 0%$_{v/v}$ →30%$_{v/v}$ ethyl acetate in cyclohexane) yielded **10a** (97 mg, 35%).

Amorphous foam; ^{1}H NMR (500 MHz, 298 K, DMSO-d_6): δ = 7.43 – 7.36 (m, 5H), 7.33 – 7.29 (m, 2H), 7.01 – 6.96 (m, 4H), 5.25 (s, 2H), 5.23 (s, 1H), 5.02 (s, 1H), 4.31 and 4.21 (ABq, J = 9.7 Hz, 2H), 4.14 – 4.10 (m, 2H), 1.52 (s, 3H), 1.31 (s, 3H), 1.25 ppm (t, J = 7.0 Hz, 3H); ^{13}C NMR (126 MHz, 298 K, DMSO-d_6): δ = 166.1, 158.0, 155.3, 152.0, 135.0, 129.5, 128.5, 128.5, 128.4, 121.3, 114.9, 81.9, 71.6, 70.5, 67.4, 63.0, 62.3, 60.3, 20.6, 17.6, 13.6 ppm; IR (film): ṽ = 1797, 1757, 1708, 1693, 1600, 1496, 1454, 1331, 1237, 1215, 1119, 1057, 1010 cm^{-1}; HRMS (ESI): m/z calculated for $C_{25}H_{29}N_2O_8S$ $[M+H]^+$: 517.1639; found: 517.1649; $[\alpha]_D^{25}$ = +159 (c = 0.008 g/mL, $CHCl_3$).

Benzyl (2*S*,5*R*,6*S*)-3,3-dimethyl-7-oxo-6-(((*R*)-2-oxopyrrolidin-3-yl)oxy)-6-(2-phenoxyacetamido)-4-thia-1-azabicyclo[3.2.0]heptane-2-carboxylate 4,4-dioxide (10b)

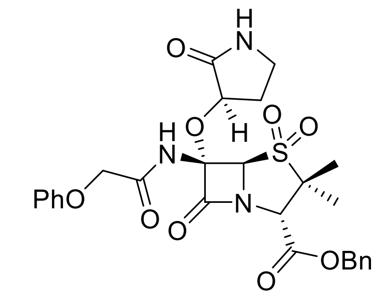

To a solution of **1** (466 mg, 0.99 mmol) in anhydrous tetrahydrofuran (10 mL was added freshly prepared ^{t}BuOCl[32] (0.17 mL, 1.47 mmol) at -30 °C, followed by Et_3N (0.15 mL, 1.08 mmol, 1.1 equiv.) and a solution of (*R*)-3-hydroxypyrrolidin-2-one (1 g, 9.9 mmol, 10 equiv.) in *N*,*N*-dimethylformamide (6 mL). The reaction mixture was stirred for 1 h at -30 °C, before AcOH (0.1 mL) was added. The mixture was diluted with CH_2Cl_2, before it was sequentially washed with saturated aqueous $NaHCO_3$ solution, aqueous $Na_2S_2O_3$ (5% (w/v)) solution, and brine. The organic layer was dried over Na_2SO_4, filtered, then concentrated *in vacuo*. Purification by flash column chromatography (25 g Sfär: 100%$_{v/v}$ cyclohexane (3 CV), then a linear gradient (9 CV): 0%$_{v/v}$ →45%$_{v/v}$ ethyl acetate in cyclohexane, then 45%$_{v/v}$ ethyl acetate in cyclohexane (4 CV)) yielded **10b** (38 mg, 7%). Note that there was evidence for an uncharacterized impurity in the ^{1}H NMR spectrum, as evidenced by peaks at δ = 2.02 – 1.95 ppm; removal of the said impurity could not be achieved using purification by flash column chromatography.

Amorphous solid; ^{1}H NMR (500 MHz, 298 K, DMSO-d_6): δ = 9.88 (s, 1H), 8.02 (s, 1H), 7.47 – 7.34 (m, 5H), 7.32 – 7.22 (m, 2H), 6.98 – 6.91 (m, 3H), 5.61 (s, 1H), 5.32 and 5.24 (ABq, *J* = 12.1 Hz, 2H), 4.73 (s, 1H), 4.68 and 4.57 (ABq, *J* = 15.8 Hz, 2H), 4.47 (t, *J* = 8.1 Hz, 1H), 3.22 – 3.18 (m, 1H), 3.13 – 3.07 (m, 1H), 2.37 – 2.29 (m, 1H), 1.94 – 1.85 (m, 1H), 1.40 (s, 3H), 1.25 ppm (s, 3H); ^{13}C NMR (126 MHz, 298 K, DMSO-d_6): δ = 172.9, 170.3, 167.2, 166.3, 157.8, 135.0, 129.4, 128.6, 128.5 (2C), 121.1, 114.6, 90.2, 72.9, 71.4, 67.5, 65.9, 63.4, 61.4, 37.8, 28.8, 19.5, 17.4 ppm; IR (film): ṽ = 1802, 1754, 1713, 1599, 1495, 1437, 1332, 1299, 1261, 1216, 1176, 1119, 1086 cm^{-1}; HRMS (ESI): m/z calculated for $C_{27}H_{30}N_3O_9S$ [M+H]$^+$: 572.1697; found: 572.1700; $[\alpha]_D^{25}$ = +79 (c = 0.008 g/mL, $CHCl_3$).

Benzyl (2*S*,5*R*,6*S*)-6-(benzyloxy)-3,3-dimethyl-7-oxo-6-(2-phenoxyacetamido)-4-thia-1-azabicyclo[3.2.0]heptane-2-carboxylate (16c)

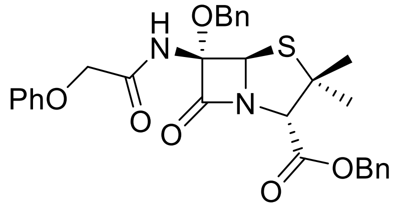

To a solution of **12** (900 mg, 1.72 mmol, 1 equiv.) in anhydrous CH_2Cl_2 (0.1 M) was added 2,3-dichloro-5,6-dicyano-1,4-benzoquinone (352 mg, 1.55 mmol, 0.9 equiv.) at room temperature. After stirring the reaction mixture for 1 h, the resultant precipitate was removed by filtration. Then, benzyl alcohol (1.71 mL, 17.2 mmol, 10 equiv.) was added to the filtrate, and the solution was stirred at room temperature for 21 h, before the reaction mixture was evaporated *in vacuo*. The presence of intermediate **14c** (benzyl (2*S*,5*R*,6*S*)-6-(benzyloxy)-6-(((*E*)-2,6-di-*tert*-butyl-4-hydroxybenzylidene)amino)-3,3-dimethyl-7-oxo-4-thia-1-azabicyclo[3.2.0]heptane-2-carboxylate) was evidenced in the crude mixture by ^{1}H NMR analysis, alongside other components and excess benzyl alcohol. The crude mixture was partially purified by flash column chromatography (25 g Sfär: 100%$_{v/v}$ cyclohexane (3 CV), then a linear gradient (33 CV): 0%$_{v/v}$ →10%$_{v/v}$ ethyl acetate in cyclohexane) to yield 350 mg of a mixture consisting of

14c, benzyl alcohol and non-characterized impurities. This mixture was then dissolved in anhydrous CH_2Cl_2 (10 mL), to which trimethylacetohydrazideammonium chloride (233 mg, 1.39 mmol, 0.8 equiv.) in anhydrous MeOH (4 mL) was added dropwise. The reaction mixture was stirred at room temperature for 3 h, then evaporated *in vacuo*. The residue was redissolved in CH_2Cl_2, and the resulting mixture was washed with H_2O, dried over Na_2SO_4, and concentrated *in vacuo* to yield crude benzyl (2*S*,5*R*,6*S*)-6-amino-6-(benzyloxy)-3,3-dimethyl-7-oxo-4-thia-1-azabicyclo[3.2.0]heptane-2-carboxylate **15c** (312 mg), which was used directly in the next step without any further purification (attempted purification by either column chromatography or recrystallisation resulted in degradation).

The crude mixture of **15c** (312 mg) was dissolved in anhydrous CH_2Cl_2 (5 mL), before dropwise addition of Et_3N (0.74 mL, 5.32 mmol) and phenoxyacetyl chloride (0.52 mL, 3.8 mmol) at 0 °C. The reaction mixture was stirred room temperature for 16 h, after which it was diluted with CH_2Cl_2. The mixture was washed with saturated aqueous $NaHCO_3$ solution and H_2O. The organic layer was dried over Na_2SO_4, filtered, and concentrated *in vacuo*. Purification by flash column chromatography (25 g Sfär: $100\%_{v/v}$ cyclohexane (2 CV), then a linear gradient (22 CV): $0\%_{v/v} \rightarrow 22\%_{v/v}$ ethyl acetate in cyclohexane) yielded **16c** (120 mg, 12%).

Amorphous foam; ^1H NMR (600 MHz, 298 K, DMSO-d_6): δ = 9.83 (s, 1H), 7.44 – 7.41 (m, 2H), 7.40 – 7.37 (m, 2H), 7.37 – 7.33 (m, 3H), 7.32 – 7.25 (m, 5H), 6.96 – 6.93 (m, 1H), 6.93 – 6.89 (m, 2H), 5.50 (s, 1H), 5.21 (d, *J* = 1.8 Hz, 2H), 4.73 – 4.65 (m, 2H), 4.65 – 4.57 (m, 3H), 1.47 (s, 3H), 1.32 ppm (s, 3H); ^{13}C NMR (151 MHz, 298 K, DMSO-d_6): δ = 168.6, 167.6, 167.0, 157.8, 137.1, 135.2, 129.4, 128.54, 128.51, 128.4, 128.3, 127.8, 127.77, 121.1, 114.6, 94.5, 74.0, 68.0, 67.4, 66.8, 66.1, 61.8, 31.9, 25.4 ppm; IR (film): ṽ = 1778, 1746, 1707, 1599, 1495, 1456, 1372, 1310, 1239, 1207, 1186, 1131, 1084, 1067, 1026 cm^{-1}; HRMS (ESI): m/z

calculated for $C_{30}H_{31}N_2O_6S$ $[M+H]^+$: 547.1897; found: 547.1897; $[\alpha]_D^{25}$ = +164 (c = 0.007 g/mL, $CHCl_3$).

Benzyl (2*S*,5*R*,6*S*)-6-(benzyloxy)-3,3-dimethyl-7-oxo-6-(2-phenoxyacetamido)-4-thia-1-azabicyclo[3.2.0]heptane-2-carboxylate 4,4-dioxide (17)

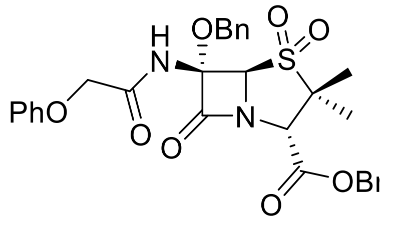

To a stirred solution of **16c** (101 mg, 0.36 mmol, 1 equiv.) in anhydrous CH_2Cl_2 (5 mL), mCPBA (<77% purity; 116 mg, 0.78 mmol, 2.2 equiv.) was added at 0 °C in portions over 5 min. The reaction mixture was stirred for 1 h at 10 °C, before being stirred at room temperature overnight for 16 h. The reaction mixture was diluted with CH_2Cl_2, washed with aqueous $Na_2S_2O_5$ (10% (w/v)) solution, saturated aqueous $NaHCO_3$ solution, water, and brine. The organic layer was dried over Na_2SO_4, filtered, then concentrated *in vacuo*. Purification by flash column chromatography (10 g Sfär: 100%$_{v/v}$ cyclohexane (2 CV), then a linear gradient (25 CV): 0%$_{v/v}$ →30%$_{v/v}$ ethyl acetate in cyclohexane) yielded **27** (45 mg, 21%).

Amorphous solid; ^{1}H NMR (500 MHz, 298 K, DMSO-d_6): δ = 7.42 – 7.35 (m, 10H), 7.34 – 7.30 (m, 2H), 7.09 (s, 1H), 7.03 – 6.97 (m, 3H), 5.29 (s, 1H), 5.24 (d, *J* = 1.8 Hz, 2H), 5.16 – 5.10 (m, 2H), 5.03 (s, 1H), 4.35 and 4.27 (ABq, *J* = 9.7 Hz, 1H), 1.53 (s, 3H), 1.32 ppm (s, 3H); ^{13}C NMR (151 MHz, 298 K, DMSO-d_6): δ = 166.1, 158.0, 155.2, 151.9, 135.3, 135.0, 129.5, 128.6, 128.50, 128.45, 128.43, 128.4, 121.3, 115.0, 82.1, 71.5, 70.5, 68.4, 67.4, 62.4, 60.3, 20.6, 17.6 ppm; IR (film): ṽ = 1797, 1757, 1708, 1693, 1600, 1496, 1454, 1331, 1237, 1215, 1177, 1119, 1057, 1010 cm^{-1}; HRMS (ESI): m/z calculated for $C_{30}H_{31}N_2O_8S$ $[M+H]^+$: 579.1796, found: 579.1802; $[\alpha]_D^{25}$ = +66 (c = 0.01 g/mL, $CHCl_3$).

Benzyl (2*S*,5*R*,6*S*)-6-methoxy-3,3-dimethyl-7-oxo-6-(2-phenylacetamido)-4-thia-1-azabicyclo[3.2.0]heptane-2-carboxylate 4,4-dioxide (20)

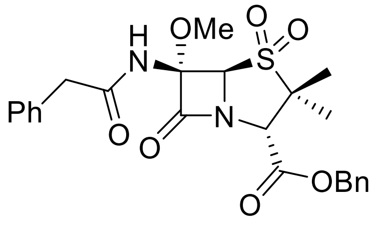

General procedure A[31] was used with penam **19** (1.17 g, 2.56 mmol, 1 equiv.). Purification by flash column chromatography (25 g Sfär: 100%$_{v/v}$ cyclohexane (2 CV), then a linear gradient (30 CV): 0%$_{v/v}$ →50%$_{v/v}$ ethyl acetate in cyclohexane) afforded **20** (453 mg, 36%).

Amorphous solid; ^{1}H NMR (600 MHz, 298 K, DMSO-d_6): δ = 9.73 (s, 1H), 7.46 – 7.22 (m, 11H), 5.51 (s, 1H), 5.31 and 5.24 (ABq, J = 12.2 Hz, 2H), 4.68 (s, 1H), 3.67 (d, J = 14.7 Hz, 1H), 3.49 (d, J = 14.7 Hz, 1H), 3.31 (s, 3H), 1.40 (s, 3H), 1.26 ppm (s, 3H); ^{13}C NMR (151 MHz, 298 K, DMSO-d_6): δ = 172.5, 167.8, 166.4, 135.2, 135.0, 129.3, 128.6, 128.5, 128.2, 126.5, 90.9, 70.3, 67.5, 63.3, 61.4, 52.5, 41.3, 40.1, 19.4, 17.4 ppm; IR (film): ṽ = 3353, 3029, 2938, 1798, 1757, 1698, 1498, 1456, 1334, 1261, 1217, 1174, 1121, 1001 cm^{-1}; HRMS (ESI): m/z calculated for $C_{24}H_{26}N_2O_7SK$ [M+K]$^+$: 525.1092, found: 525.1093; $[\alpha]_D^{25}$ = +107 (c = 0.01 g/mL, $CHCl_3$).

Benzyl (2*S*,5*R*,6*S*)-6-methoxy-3,3-dimethyl-7-oxo-6-(2-phenylacetamido)-4-thia-1-azabicyclo[3.2.0]heptane-2-carboxylate 4-oxide (22)

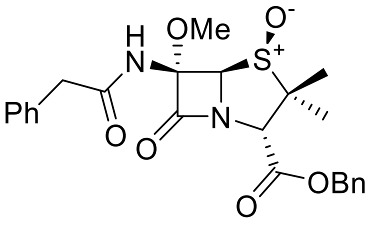

General procedure A[31] was used with penam **21** (750 mg, 1.7 mmol, 1 equiv.). Purification by flash column chromatography (25 g Sfär: 100%$_{v/v}$ cyclohexane (2 CV), then a linear gradient (30 CV): 0%$_{v/v}$ →30%$_{v/v}$ ethyl acetate in cyclohexane) afforded **22** (257 mg, 54%).

Amorphous foam; ^{1}H NMR (500 MHz, 298 K, DMSO-d_6): δ = 9.56 (s, 1H), 7.46 – 7.35 (m, 5H), 7.31 – 7.22 (m, 5H), 5.36 (s, 1H), 5.30 and 5.22 (ABq, J = 12.2 Hz, 2H), 4.48 (s, 1H), 3.62 and 3.45 (ABq, J = 14.9 Hz, 2H), 3.29 (s, 3H), 1.46 (s, 3H), 1.08 ppm (s, 3H); ^{13}C NMR (126 MHz, 298 K, DMSO-d_6): δ = 171.8, 167.7, 167.2, 135.5, 135.2, 129.4, 128.6, 128.6, 128.5, 128.1, 126.4, 90.6, 78.3, 70.4, 67.3, 63.4, 52.2, 41.3, 19.2, 17.7 ppm; IR (film): ṽ = 1789, 1751, 1684, 1522, 1497, 1457, 1377, 1318, 1261, 1215, 1165, 1077, 1057, 1003 cm^{-1}; HRMS (ESI): m/z calculated for $C_{24}H_{26}N_2O_6SNa$ $[M+Na]^+$: 493.1404, found: 493.1402; $[\alpha]_D^{25}$ = +163 (c = 0.02 g/mL, $CHCl_3$).

Benzyl (2*S*,4*R*,5*R*,6*R*)-3,3-dimethyl-7-oxo-6-(2-phenylacetamido)-4-thia-1-azabicyclo[3.2.0]heptane-2-carboxylate 4-oxide (23)

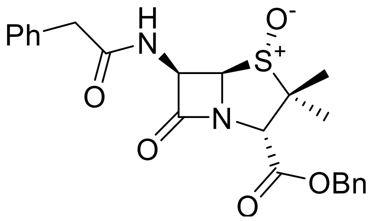

To a solution of penam **18** (1.55 g, 3.65 mmol, 1 equiv.) in tetrahydrofuran (10 mL) were sequentially added 2,6-lutidine (1 mL, 8.6 mmol, 2.4 equiv.), H_2O (1 mL), and freshly prepared ^{t}BuOCl[32] (4 mmol, 0.46 mL, 1.1 equiv.) at -5 °C. The reaction mixture was stirred at -5 °C for 5 min, before addition of an aqueous H_2SO_4 solution (4 mL; 2.5 M). The mixture was then poured into an aqueous HCl solution (100 mL; 1 M), and was extracted with EtOAc (100 mL). The organic layer was separated and washed with saturated aqueous $NaHCO_3$ solution, and brine. The organic layer was then dried over Na_2SO_4, filtered, and concentrated *in vacuo*. Purification by flash column chromatography (25 g Sfär: 100%$_{v/v}$ cyclohexane (2 CV), then a linear gradient (23 CV): 0%$_{v/v}$→43%$_{v/v}$ ethyl acetate in cyclohexane, then 43%$_{v/v}$ ethyl acetate in cyclohexane (7 CV), then a linear gradient (8 CV): 43%$_{v/v}$ →58%$_{v/v}$ ethyl acetate in cyclohexane, then 58% ethyl acetate in cyclohexane (10 CV), then a linear gradient (3 CV): 58%$_{v/v}$ →90%$_{v/v}$ ethyl acetate in cyclohexane; followed by 90%$_{v/v}$ ethyl acetate in cyclohexane (5 CV)) afforded penam **23** (360 mg, 22%).

Amorphous foam; ^{1}H NMR (500 MHz, 298 K, DMSO-d_6): δ = 9.26 (d, J = 7.4 Hz, 1H), 7.46 – 7.35 (m, 5H), 7.32 – 7.20 (m, 5H), 5.44 (dd, J = 7.3, 4.2 Hz, 1H), 5.28 – 5.18 (m, 2H), 4.77 (d, J = 4.3 Hz, 1H), 4.53 (s, 1H), 3.56 (s, 3H), 1.52 (s, 3H), 1.11 ppm (s, 3H); ^{13}C NMR (126 MHz, 298 K, DMSO-d_6): δ = 170.9, 170.8, 167.3, 135.4, 135.2, 129.0, 128.59, 128.55, 128.5, 128.3, 126.6, 78.8, 68.4, 67.2, 64.1, 57.3, 41.4, 23.7, 15.4 ppm; IR (film): ṽ = 1793, 1748, 1664, 1526, 1498, 1456, 1347, 1300, 1253, 1199, 1161, 1057 cm^{-1}; HRMS (ESI): m/z calculated for $C_{23}H_{24}N_2O_5SNa$ [M+Na]$^+$: 463.1298, found: 493.1402; $[\alpha]_D^{25}$ = +91 (c = 0.002 g/mL, $CHCl_3$).

Benzyl (2*S*,4*R*,5*R*,6*S*)-6-methoxy-3,3-dimethyl-7-oxo-6-(2-phenylacetamido)-4-thia-1-azabicyclo[3.2.0]heptane-2-carboxylate 4-oxide (3)

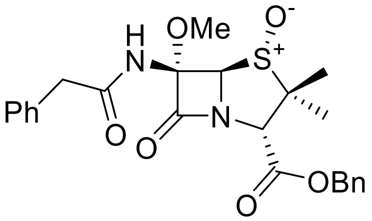

General procedure A[31] was used with penam **23** (190 mg, 0.43 mmol, 1 equiv.). Purification by flash column chromatography (10 g Sfär: 100% cyclohexane (2 CV), then a linear gradient (16 CV): $0\%_{v/v} \rightarrow 32\%_{v/v}$ ethyl acetate in cyclohexane, then $32\%_{v/v}$ ethyl acetate in cyclohexane (5 CV), then a linear gradient (10 CV): $32\%_{v/v} \rightarrow 52\%_{v/v}$ ethyl acetate in cyclohexane, then $52\%_{v/v}$ ethyl acetate in cyclohexane (3 CV)) afforded **3** (75 mg, 37%).

Amorphous foam; ^{1}H NMR (500 MHz, 298 K, DMSO-d_6): δ = 9.92 (s, 1H), 7.45 – 7.23 (m, 10H), 5.23 – 5.17 (m, 2H), 4.85 (s, 1H), 4.83 (s, 1H), 3.61 – 3.53 (m, 2H), 3.35 (s, 3H), 1.21 (s, 3H), 1.15 ppm (s, 3H); ^{13}C NMR (126 MHz, 298 K, DMSO-d_6): δ = 171.4, 166.9, 166.1, 135.1, 135.0, 129.2, 129.1, 128.5, 128.4, 128.2, 126.6, 93.2, 89.0, 69.1, 67.1, 65.1, 52.9, 41.7, 23.3, 16.4 ppm; IR (film): ṽ = 1787, 1737, 1687, 1550, 1523, 1455, 1327, 1159, 1044 cm^{-1}; HRMS (ESI): m/z calculated for $C_{24}H_{26}N_2O_6SK$ [M+K]$^+$: 509.1143, found: 509.1150; $[\alpha]_D^{25}$ = +75 (c = 0.003 g/mL, $CHCl_3$).

(2*S*,5*R*,6*S*)-6-Methoxy-3,3-dimethyl-7-oxo-6-(2-phenylacetamido)-4-thia-1-azabicyclo[3.2.0]heptane-2-carboxylic acid (24)[36]

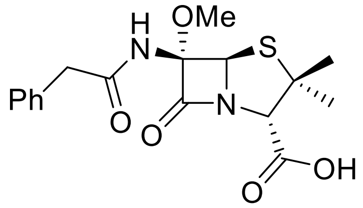

To a solution of commercially-sourced penicillin G sodium (1 g, 2.8 mmol, 1 equiv.) in *N*,*N*-dimethylformamide (14.4 mL) and methanol (2.2 mL) was added dropwise a solution of 1 M LiOMe in MeOH (4.8 mL) at -78 °C, followed by freshly prepared ^{t}BuOCl[32] (0.54 mL, 4.7 mmol, 1.7 equiv.). The reaction mixture was stirred for 15 min at -78 °C, before the addition of concentrated H_2SO_4 (0.07 mL). The reaction mixture was warmed to room temperature, before it was diluted with water and acidified to pH 2 by addition of an aqueous H_2SO_4 solution (1 M). The mixture was extracted with $CHCl_3$, and the combined organic extracts were sequentially washed with saturated aqueous LiCl solution, H_2O, and brine. The organic extracts were dried over Na_2SO_4, concentrated *in vacuo*, and purified by reverse-phase flash column chromatography (30 g Sfär C18 D: 100%$_{v/v}$ aqueous formic acid solution (0.1%) in H_2O (10 CV), then a linear gradient (20 CV): 0%$_{v/v}$ → 100%$_{v/v}$ aqueous formic acid solution (0.1%) in CH_3CN). The fractions containing the purified **24** were collected and lyophilised to afford the desired product (247 mg, 18%). The analytical data of **24** are consistent with those reported.[36]

Amorphous solid; ^{1}H NMR (600 MHz, 298 K, DMSO-*d$_6$*): δ = 9.62 (s, 1H), 7.30 – 7.23 (m, 5H), 5.33 (s, 1H), 4.32 (s, 1H), 3.58 and 3.50 (ABq, *J* = 14.1 Hz, 2H), 3.31 (s, 3H), 1.42 (s, 3H), 1.38 ppm (s, 3H); ^{13}C NMR (126 MHz, 298 K, DMSO-*d$_6$*): δ = 170.8, 168.7, 167.9, 135.5, 129.2, 128.1, 126.4, 94.7, 73.5, 68.3, 61.0, 52.7, 41.9, 40.4, 39.7, 31.7, 25.8 ppm; IR (film): ṽ = 1780, 1749, 1672, 1661, 1516, 1497, 1457, 1392, 1371, 1300, 1260, 1180, 1100, 1000 cm^{-1}; HRMS (ESI): m/z calculated for $C_{17}H_{20}N_2O_5SNa$ $[M+Na]^+$: 387.0985; found: 387.0982; $[\alpha]_D^{25}$ = +435 (c = 0.001 g/mL, $CHCl_3$).

Benzyl (2*S*,5*R*,6*S*)-6-methoxy-3,3-dimethyl-7-oxo-6-(2-phenylacetamido)-4-thia-1-azabicyclo[3.2.0]heptane-2-carboxylate (25)[36]

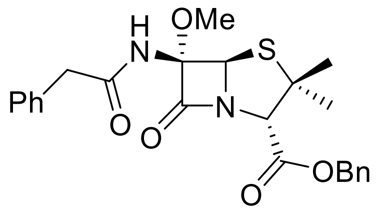

To a solution of **24** (172 mg, 0.47 mmol) in anhydrous *N,N*-dimethylformamide (5 mL) at room temperature were sequentially added $NaHCO_3$ (59 mg, 0.7 mmol, 1.5 equiv.), and BnBr (0.08 mL, 0.67 mmol, 1.42 equiv.), and the reaction mixture was stirred for 16 h, and after thin-layer chromatography analysis indicated complete conversion of the starting material, it was poured into ice water and extracted with EtOAc. The organic layer washed with saturated aqueous LiCl solution, saturated aqueous $NaHCO_3$ solution, and brine, then it was dried over Na_2SO_4, filtered, and concentrated *in vacuo*. Purification by flash column chromatography (10 g Sfär: $100\%_{v/v}$ cyclohexane (7 CV), then a linear gradient (20 CV): $0\%_{v/v} \rightarrow 30\%_{v/v}$ ethyl acetate in cyclohexane) yielded ester **25** (121 mg, 49%). The analytical data of **25** are consistent with those reported.[36]

Amorphous solid; ^{1}H NMR (600 MHz, 298 K, DMSO-d_6): δ = 9.65 (s, 1H), 7.44 – 7.33 (m, 5H), 7.32 – 7.25 (m, 4H), 7.24 – 7.20 (m, 1H), 5.36 (s, 1H), 5.22 – 5.17 (m, 2H), 4.55 (s, 1H), 3.58 and 3.50 (ABq, J = 14.1 Hz, 2H), 3.31 (s, 3H), 1.40 (s, 3H), 1.27 ppm (s, 3H); ^{13}C NMR (151 MHz, 298 K, DMSO-d_6): δ = 170.8, 167.9, 167.0, 135.4, 135.2, 129.2, 128.5, 128.5, 128.4, 128.1, 126.5, 94.9, 73.7, 67.9, 66.8, 61.4, 52.7, 41.9, 31.8, 25.4 ppm; IR (film): ṽ = 1777, 1749, 1686, 1647, 1522, 1498, 1458, 1375, 1261, 1208, 1065 cm^{-1}; HRMS (ESI): m/z calculated for $C_{24}H_{27}N_2O_5S$ $[M+H]^+$: 455.1635, found: 455.1633; $[\alpha]_D^{25}$ = +165 (c = 0.002 g/mL, $CHCl_3$).

Benzyl (2*S*,5*R*,6*S*)-6-methoxy-3,3-dimethyl-7-oxo-4-thia-1-azabicyclo[3.2.0]heptane-2-carboxylate (26a)[37]

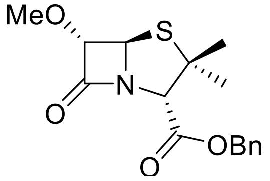

General procedure B[33] was used with penam **11** (1.6 g, 3.34 mmol) and MeOH (14 mL) as the solvent. Purification by flash column chromatography (25 g Sfär: 100%$_{v/v}$ cyclohexane (2 CV), then a linear gradient (20 CV): 0%$_{v/v}$ →20%$_{v/v}$ ethyl acetate in cyclohexane) yielded **26a** (383 mg, 1.19 mmol, 37%). The analytical data of **26a** are consistent with those reported.[37]

Yellow oil; ^{1}H NMR (400 MHz, 298 K, $CDCl_3$): δ = 7.38 – 7.36 (m, 5H), 5.31 (d, *J* = 1.4 Hz, 1H,), 5.22 – 5.15 (m, 2H), 4.58 (d, *J* = 1.4 Hz, 1H), 4.52 (s, 1H), 3.53 (s, 3H), 1.54 (s, 3H), 1.39 ppm (s, 3H); ^{13}C NMR (101 MHz, 298 K, $CDCl_3$): δ = 169.5, 167.4, 134.9, 128.8, 128.8, 92.7, 77.4, 69.1, 68.6, 67.5, 64.5, 57.9, 33.9, 25.8 ppm; IR (film): ṽ = 2931, 2359, 1781, 1746, 1684, 1543, 1499, 1456, 1373, 1286, 1206, 1128, 1082, 1040 cm^{-1}; HRMS (ESI): m/z calculated for $C_{16}H_{19}NO_4S$ [M+Na]$^+$: 344.0927, found: 344.0932; $[\alpha]_D^{25}$ = +96 (0.012 g/mL, $CHCl_3$).

Benzyl (2*S*,5*R*,6*S*)-6-ethoxy-3,3-dimethyl-7-oxo-4-thia-1-azabicyclo[3.2.0]heptane-2-carboxylate (26b)[38]

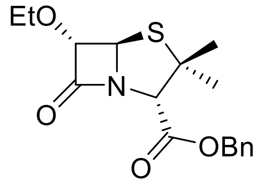

General procedure B[33] was used with penam **11** (1.6 g, 3.34 mmol) and EtOH (15 mL) as the solvent. Purification by flash column chromatography (25 g Sfär: 100%$_{v/v}$ cyclohexane (2 CV), then a linear gradient (20 CV): 0%$_{v/v}$ →20%$_{v/v}$ ethyl acetate in cyclohexane) yielded **26b** (323 mg, 0.96 mmol, 36%). The analytical data of **26b** are consistent with those reported.[38]

Yellow oil; ^{1}H NMR (500 MHz, 298 K, DMSO-d_6): δ = 7.45 – 7.34 (m, 5H), 5.27 (d, *J* = 1.3 Hz, 1H), 5.20 (s, 2H), 4.73 (d, *J* = 1.4 Hz, 1H), 4.62 (s, 1H), 3.64 (q, *J* = 7.0 Hz, 2H), 1.49 (s, 3H), 1.33 (s, 3H), 1.15 ppm (t, *J* = 7.0 Hz, 3H); ^{13}C NMR (126 MHz, 298 K, DMSO-d_6): δ =

169.4, 167.1, 135.3, 128.6, 128.5, 128.4, 89.9, 68.4, 68.3, 66.8, 65.6, 64.1, 32.4, 25.2, 15.0 ppm; IR (film): ṽ = 1779, 1747, 1560, 1578, 1391, 1354, 1299, 1202 cm^{-1}; HRMS (ESI): m/z calculated for $C_{17}H_{22}NO_4S$ $[M+H]^+$: 336.1264, found: 336.1267; $[\alpha]_D^{25}$ = +110 (c = 0.013 g/mL, $CHCl_3$).

Benzyl (2*S*,5*R*,6*S*)-6-(*iso*-propoxy)-3,3-dimethyl-7-oxo-4-thia-1-azabicyclo[3.2.0]heptane-2-carboxylate (26c)

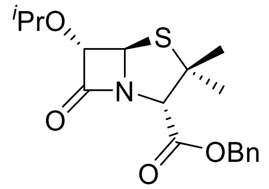

General procedure B[33] was used with penam **11** (1.6 g, 3.34 mmol) and ^{i}PrOH (15 mL) as the solvent. Purification by flash column chromatography (25 g Sfär: 100%$_{v/v}$ cyclohexane (2 CV), then a linear gradient (20 CV): 0%$_{v/v}$ →20%$_{v/v}$ ethyl acetate in cyclohexane) yielded **26c** (288 mg, 0.82 mmol, 36%).

Yellow oil; ^{1}H NMR (600 MHz, 298 K, DMSO-*d₆*): 7.44 – 7.33 (m, 5H), 5.20 (s, 2H), 5.17 (d, *J* = 1.4 Hz, 1H), 4.74 (d, *J* = 1.4 Hz, 1H), 4.62 (s, 1H), 3.84 (hept, *J* = 6.1 Hz, 1H), 1.49 (s, 3H), 1.34 (s, 3H), 1.15 (d, *J* = 6.1 Hz, 3H), 1.12 ppm (d, *J* = 6.1 Hz, 3H); ^{13}C NMR (151 MHz, 298 K, DMSO-d_6): δ = 169.7, 167.1, 135.3, 128.6, 128.5, 128.4, 88.7, 72.9, 69.7, 68.3, 66.8, 64.0, 32.4, 25.2, 22.3, 22.1 ppm; IR (film): ṽ = 1781, 1748, 1464, 1377, 1301, 1260, 1203, 1185, 1129, 1027 cm^{-1}; HRMS (ESI): m/z calculated for $C_{18}H_{23}NO_4SNa$ $[M+Na]^+$: 372.1240; found: 372.1246; $[\alpha]_D^{25}$ = +145 (c = 0.003 g/mL, $CHCl_3$).

Benzyl (2*S*,5*R*,6*S*)-6-(*tert*-butoxy)-3,3-dimethyl-7-oxo-4-thia-1-azabicyclo[3.2.0]heptane-2-carboxylate (26d)

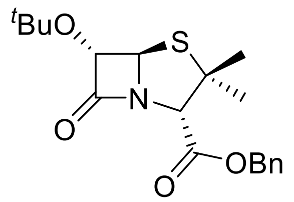

General procedure B[33] was used with penam **11** (1.6 g, 3.34 mmol) and ^{t}BuOH (15 mL) as the solvent. Purification by flash column chromatography (25 g Sfär: 100%$_{v/v}$ cyclohexane (2 CV), then a linear gradient (20 CV): 0%$_{v/v}$ →20%$_{v/v}$ ethyl acetate in cyclohexane) yielded **26d** (285 mg, 0.78 mmol, 35%).

Yellow oil; ^{1}H NMR (500 MHz, 298 K, DMSO-*d₆*): δ = 7.44 – 7.33 (m, 5H), 5.20 (s, 2H), 5.12 (d, *J* = 1.4 Hz, 1H), 4.77 (d, *J* = 1.4 Hz, 1H), 4.62 (s, 1H), 1.51 (s, 3H), 1.34 (s, 3H), 1.18 ppm (s, 9H); ^{13}C NMR (126 MHz, 298 K, DMSO-d_6): δ = 170.2, 167.1, 135.3, 128.6, 128.5, 128.4, 84.5, 76.0, 70.9, 68.4, 66.8, 63.9, 32.5, 27.5, 25.3 ppm; IR (film): ṽ = 1782, 1749, 1458, 1392, 1370, 1301, 1262, 1238, 1181.33, 1119, 1090, 1025 cm^{-1}; HRMS (ESI): m/z calculated for $C_{19}H_{25}NO_4S$ [M+Na]$^+$: 386.1397, found: 386.1402; $[\alpha]_D^{25}$ = +42 (c = 0.008 g/mL, $CHCl_3$).

Benzyl (2*S*,5*R*,6*S*)-3,3-dimethyl-7-oxo-6-phenoxy-4-thia-1-azabicyclo[3.2.0]heptane-2-carboxylate (26e)

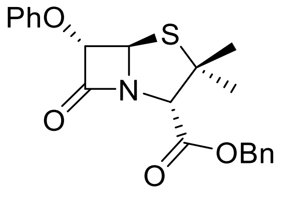

General procedure B[33] was applied using penam **11** (1.6 g, 3.34 mmol) and PhOH (470 mg, 5 mmol). Purification by flash column chromatography (25 g Sfär, 100%$_{v/v}$ cyclohexane (3 CV), followed by a linear gradient (25 CV): 0%$_{v/v}$ →20%$_{v/v}$ ethyl acetate in cyclohexane) resulted in partial purification. The fractions enriched in **26e** were combined and concentrated *in vacuo*, and the obtained oil was then purified a second time by flash column chromatography (25 g Sfär: 100%$_{v/v}$ cyclohexane (3 CV), then a linear gradient (13 CV): 0%$_{v/v}$ →3%$_{v/v}$ acetone in cyclohexane, then a linear gradient (7 CV): 3%$_{v/v}$ →10%$_{v/v}$ acetone in cyclohexane) to give **26e** (178 mg, 15%).

Colourless oil; ^{1}H NMR (400 MHz, 298 K, $CDCl_3$): δ = 7.40 – 7.36 (m, 5H), 7.34 – 7.30 (m, 2H), 7.07 – 7.02 (m, 1H), 6.96 – 6.91 (m, 2H), 5.39 (d, J = 1.4 Hz, 1H), 5.24 – 5.16 (m, 3H), 4.60 (s, 1H), 1.61 (s, 3H), 1.43 ppm (s, 3H); ^{13}C NMR (126 MHz, 298 K, $CDCl_3$): δ = 167.9, 167.2, 157.0, 134.9, 130.0, 128.88, 128.87, 128.82, 122.7, 115.4, 88.6, 69.6, 69.4, 67.6, 64.5, 34.2, 25.7 ppm; IR (film): ṽ = 2360, 2341, 1800, 1757, 1630, 1579, 1501, 1464, 1423, 1383, 1320, 1286, 1194, 1159, 1118, 1089 cm^{-1}; HRMS (ESI): m/z calculated for $C_{21}H_{22}NO_4S$ $[M+H]^+$: 384.1264, found: 384.1259; $[\alpha]_D^{25}$ = +94 (0.004 g/mL, $CHCl_3$).

Benzyl (2*S*,5*R*,6*S*)-6-(benzyloxy)-3,3-dimethyl-7-oxo-4-thia-1-azabicyclo[3.2.0]heptane-2-carboxylate (26f)

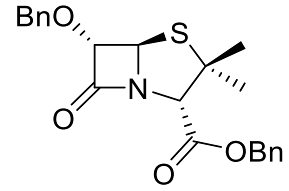

General procedure B[33] was used with penam **11** (1.6 g, 3.34 mmol) and BnOH (15 mL) as the solvent. Purification by flash column chromatography (25 g Sfär: 100%$_{v/v}$ cyclohexane (5 CV), then a linear gradient (40 CV): 0%$_{v/v}$ →10%$_{v/v}$ ethyl acetate in cyclohexane) yielded **26f** (221 mg, 0.55 mmol, 18%).

Colourless oil; ^{1}H NMR (400 MHz, 298 K, $CDCl_3$): δ = 7.39 – 7.33 (m, 10H), 5.21 – 5.13 (m, 2H), 5.15 (d, J = 1.4 Hz, 1H), 4.79 and 4.63 (ABq, J = 11.5 Hz, 2H), 4.67 (d, J = 1.4 Hz, 1H), 4.50 (s, 1H), 1.52 (s, 3H), 1.36 ppm (s, 3H); ^{13}C NMR (126 MHz, 298 K, $CDCl_3$): δ = 169.5, 167.4, 136.4, 134.9, 128.83, 128.82 (2 C), 128.75, 128.6, 128.5, 91.1, 73.2, 69.4, 69.1, 67.5, 64.3, 34.0, 25.7 ppm; IR (film): ṽ = 2963, 1779, 1746, 1498, 1455, 1372, 1352, 1302, 1261, 1203, 1182, 1156, 1124, 1090, 1026 cm^{-1}; HRMS (ESI): m/z calculated for $C_{22}H_{23}NO_4S$ $[M+Na]^+$: 420.1240, found: 420.1244; $[\alpha]_D^{25}$ = +143 (c = 0.008 g/mL, $CHCl_3$);

Benzyl (2*S*,5*R*,6*S*)-6-methoxy-3,3-dimethyl-7-oxo-4-thia-1-azabicyclo[3.2.0]heptane-2-carboxylate 4,4-dioxide (27a)

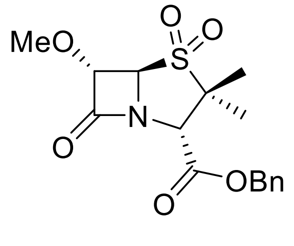

General procedure C[10] was used with penam **26a** (115 mg, 0.358 mmol), yielding after work-up crude **27a** (95 mg). A portion of **27a** (25 mg) was purified by reverse phase flash column chromatography (6 g Sfär C18 D: 100%$_{v/v}$ aqueous formic acid solution (0.1%) in H_2O (3 CV), then a linear gradient (16 CV): 0%$_{v/v}$ → 100%$_{v/v}$ aqueous formic acid solution (0.1%) in CH_3CN). The collected fractions containing product were lyophilised to give **27a** (16 mg).

Amorphous solid; ^{1}H NMR (500 MHz, 298 K, DMSO-*d*$_6$): δ = 7.45 – 7.36 (m, 5H), 5.49 (d, *J* = 1.3 Hz, 1H), 5.32 – 5.21 (m, 2H), 5.05 (d, *J* = 1.3 Hz, 1H), 4.73 (s, 1H), 3.47 (s, 3H), 1.43 (s, 3H), 1.28 ppm (s, 3H); ^{13}C NMR (126 MHz, 298 K, DMSO-*d*$_6$): δ = 169.0, 166.4, 135.0, 128.57, 128.56, 128.54 83.5, 67.5, 65.7, 62.9, 61.7, 57.6, 19.3, 17.4 ppm; IR (film): $\tilde{v}$ = 2358, 2335, 1802, 1753, 1458, 1324, 1284, 1217, 1191, 1118, 1087 cm^{-1}; HRMS (ESI): m/z calculated for $C_{16}H_{19}NO_6SNa$ [M+Na]$^+$: 376.0825, found: 376.0833; $[\alpha]_D^{25}$ = +157 (c = 0.009 g/mL, $CHCl_3$).

Benzyl (2*S*,5*R*,6*S*)-6-ethoxy-3,3-dimethyl-7-oxo-4-thia-1-azabicyclo[3.2.0]heptane-2-carboxylate 4,4-dioxide (27b)[38]

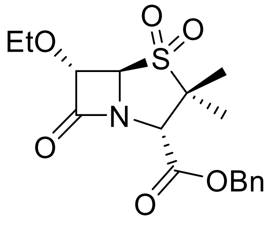

General procedure C[10] was used with penam **26b** (100 mg, 0.298 mmol), yielding after work-up crude **27b** (85 mg). A portion of **27b** (10 mg) was further purified by reverse phase flash column chromatography (6 g Sfär C18 D: 100%$_{v/v}$ aqueous formic acid solution (0.1%) in H_2O (3 CV), then a linear gradient (10 CV): 0%$_{v/v}$ → 100%$_{v/v}$ aqueous

formic acid solution (0.1%) in CH_3CN). The collected fractions containing product were lyophilised to yield **27b** (4 mg). The analytical data of **27b** are consistent with those reported.[38]

Amorphous solid; ^{1}H NMR (600 MHz, 298 K, DMSO-d_6): δ = 7.46 – 7.36 (m, 5H), 5.43 (d, *J* = 1.3 Hz, 1H), 5.29 and 5.23 (ABq, *J* = 12.2 Hz, 2H), 5.05 (d, *J* = 1.3 Hz, 1H), 4.72 (s, 1H), 3.75 (dq, *J* = 9.4, 7.0 Hz, 1H), 3.64 (dq, *J* = 9.4, 7.0 Hz, 1H), 1.43 (s, 3H), 1.27 (s, 3H), 1.18 ppm (t, *J* = 7.0 Hz, 3H); ^{13}C NMR (151 MHz, 298 K, DMSO-d_6): δ = 169.2, 166.4, 135.0, 128.6 (2 C), 128.5, 82.2, 67.5, 66.4, 66.3, 62.9, 61.7, 19.2, 17.4, 14.7 ppm; IR (film): ṽ = 1799, 1757, 1559, 1460, 1376, 1322, 1286, 1195, 1159, 1118, 1089, 1041 cm^{-1}; HRMS (ESI): m/z calculated for $C_{17}H_{21}NO_6SNa$ $[M+Na]^+$: 390.0982, found: 390.0976; $[\alpha]_D^{25}$ = +113 (c = 0.007 g/mL, $CHCl_3$).

Benzyl (2*S*,5*R*,6*S*)-6-(*iso*-propoxy)-3,3-dimethyl-7-oxo-4-thia-1-azabicyclo[3.2.0]heptane-2-carboxylate 4,4-dioxide (27c)

General procedure C[10] was used with penam **26c** (100 mg, 0.286 mmol). After work-up, no further purification was required, yielding the product **27c** (42 mg, 38%).

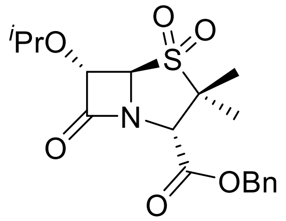

Amorphous solid; ^{1}H NMR (500 MHz, 298 K, $CDCl_3$): δ = 7.41 – 7.35 (m, 5H), 5.28 and 5.19 (ABq, *J* = 12.0 Hz, 2H), 5.02 (d, *J* = 1.5 Hz, 1H), 4.53 (d, *J* = 1.5 Hz, 1H), 4.39 (s, 1H), 3.91 – 3.83 (m 1H), 1.52 (s, 3H), 1.29 – 1.22 ppm (m, 9H); ^{13}C NMR (126 MHz, 298 K, $CDCl_3$): δ = 169.6, 166.5, 134.5, 129.2, 129.02, 128.98, 82.7, 75.4, 69.4, 68.4, 63.5, 62.8, 22.6, 22.1, 20.0, 18.8 ppm; IR (film): ṽ = 1799, 1757, 1458, 1381, 1320, 1285, 1193, 1159, 1117, 1088 cm^{-1}; HRMS (ESI): m/z calculated for $C_{18}H_{23}NO_6SNa$ $[M+Na]^+$: 404.1138, found: 404.1141; $[\alpha]_D^{25}$ = +133 (c = 0.007 g/mL, $CHCl_3$).

Benzyl (2*S*,5*R*,6*S*)-6-(*tert*-butoxy)-3,3-dimethyl-7-oxo-4-thia-1-azabicyclo[3.2.0]heptane-2-carboxylate 4,4-dioxide (27d)

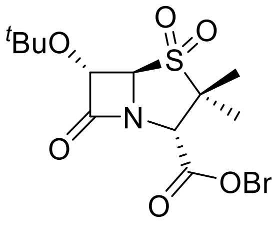

General procedure C[10] was used with penam **26d** (81 mg, 0.223 mmol). After work-up, no further purification was required, yielding the product **27d** (46 mg, 55%).

Amorphous solid; ^{1}H NMR (400 MHz, 298 K, $CDCl_3$): δ = 7.40 – 7.37 (m, 5H), 5.28 and 5.19 (ABq, *J* = 12.0 Hz, 2H), 5.13 (d, *J* = 1.5 Hz, 1H), 4.49 (d, *J* = 1.5 Hz, 1H), 4.38 (s, 1H), 1.52 (s, 3H), 1.29 (s, 9H), 1.26 ppm (s, 3H); ^{13}C NMR (126 MHz, 298 K, $CDCl_3$): δ = 170.2, 166.5, 134.5, 129.2, 129.01, 128.97, 78.0, 77.6, 70.3, 68.3, 63.6, 62.8, 27.7, 20.0, 18.9 ppm; IR (film): ṽ = 1800, 1758, 1463, 1372, 1321, 1285, 1186, 1116, 1090 cm^{-1}; HRMS (ESI): m/z calculated for $C_{19}H_{25}NO_6SK$ $[M+K]^+$: 434.1034, found: 434.1043; $[\alpha]_D^{25}$ = +130 (c = 0.003 g/mL, $CHCl_3$).

Benzyl (2*S*,5*R*,6*S*)-3,3-dimethyl-7-oxo-6-phenoxy-4-thia-1-azabicyclo[3.2.0]heptane-2-carboxylate 4,4-dioxide (27e)

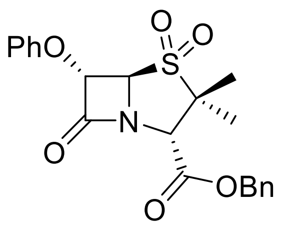

General procedure C[10] was used with penam **26e** (137 mg, 0.357 mmol). Purification by flash column chromatography (10 g Sfär: 100%$_{v/v}$ cyclohexane (0 CV), then a linear gradient (40 CV): 0%$_{v/v}$ →10%$_{v/v}$ ethyl acetate in cyclohexane) yielded **27e** (42 mg, 28%).

Colourless oil; ^{1}H NMR (500 MHz, 298 K, $CDCl_3$): δ = 7.42 – 7.37 (m, 5H), 7.37 – 7.32 (m, 2H), 7.11 – 7.07 (m, 1H), 7.03 – 6.98 (m, 2H), 5.60 (d, *J* = 1.5 Hz, 1H), 5.31 and 5.21 (ABq, *J* = 12.0 Hz, 2H), 4.73 (d, *J* = 1.5 Hz, 1H), 4.49 (s, 1H), 1.57 (s, 3H), 1.29 ppm (s, 3H); ^{13}C NMR (126 MHz, 298 K, $CDCl_3$): δ = 167.5, 166.3, 156.5, 134.4, 130.2, 129.2, 129.04, 129.02, 123.5, 115.4, 81.1, 68.5, 68.3, 63.7, 63.1, 20.0, 18.8 ppm; IR (film): ṽ = 2361, 2340, 1805, 1758,

1599, 1496, 1458, 1324, 1290, 1236, 1192, 1119, 1088 cm^{-1}; HRMS (ESI): m/z calculated for $C_{21}H_{21}NO_6SNa$ $[M+Na]^+$: 438.0982, found: 438.0983; $[\alpha]_D^{25}$ = +106 (c = 0.008 g/mL, $CHCl_3$).

Benzyl (2*S*,5*R*,6*S*)-6-(benzyloxy)-3,3-dimethyl-7-oxo-4-thia-1-azabicyclo[3.2.0]heptane-2-carboxylate 4,4-dioxide (27f)

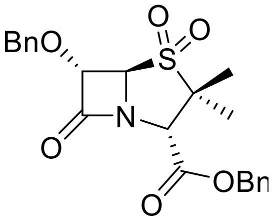

General procedure C[10] was used with penam **26f** (105 mg, 0.264 mmol), yielding after work-up crude **27f** (76 mg, 67%). A portion (20 mg) was purified by reverse phase flash column chromatography (6 g Sfär C18 D: 100%$_{v/v}$ aqueous formic acid solution (0.1%) (3 CV), then a linear gradient (10 CV): 0%$_{v/v}$ → 100%$_{v/v}$ aqueous formic acid solution (0.1%) in CH_3CN). The fractions containing product were lyophilised to yield **27f** (8 mg).

Amorphous solid; ^{1}H NMR (500 MHz, 298 K, DMSO-d_6): δ = 7.46 – 7.33 (m, 10H), 5.42 (d, *J* = 1.3 Hz, 1H), 5.30 and 5.23 (ABq, *J* = 12.1 Hz, 2H), 5.15 (d, *J* = 1.3 Hz, 1H), 4.77 and 4.69 (ABq, *J* = 11.1 Hz, 2H), 4.74 (s, 1H), 1.42 (s, 3H), 1.27 ppm (s, 3H); ^{13}C NMR (126 MHz, 298 K, DMSO-d_6): δ = 169.0, 166.4, 136.1, 135.0, 128.6, 128.55, 128.50, 128.35, 128.3, 82.0, 72.4, 67.5, 66.5, 62.9, 61.7, 19.2, 17.4; IR (film): ṽ = 2925, 1799, 1757, 1498, 1457, 1322, 1287, 1191, 1117, 1088 cm^{-1}; HRMS (ESI): m/z calculated for $C_{22}H_{23}NO_6SNa$ $[M+Na]^+$: 452.1138, found: 452.1143; $[\alpha]_D^{25}$ = +129 (c = 0.008 g/mL, $CHCl_3$).

6. References

1. L. Verbist, *Antimicrob. Agents Chemother.*, 1982, **22**, 157–161.
2. J. Hamilton-Miller and W. Brumfitt, *Infection*, 1975, **3**, 183–188.
3. R. N. Brogden, R. C. Heel, T. M. Speight and G. S. Avery, *Drugs*, 1979, **17**, 1–37.
4. R. N. Jones, *Diagn. Microbiol. Infect. Dis.*, 1989, **12**, 367–379.
5. R. H. Prabhala, H. Thadepalli, B. Rao, M. B. Bansal and R. Marshall, *Antimicrob. Agents Chemother.*, 1985, **27**, 640–642.
6. R. Wise, J. M. Andrews and J. Hancox, *Antimicrob. Agents Chemother.*, 1982, **21**, 486–491.
7. A. A. Carmine, R. N. Brogden, R. C. Heel, J. A. Romankiewicz, T. M. Speight and G. S. Avery, *Drugs*, 1983, **26**, 279–333.
8. A. Imada, K. Kitano, K. Kintaka, M. Muroi and M. Asai, *Nature*, 1981, **289**, 590–591.
9. D. R. Owen, C. M. N. Allerton, A. S. Anderson, L. Aschenbrenner, M. Avery, S. Berritt, B. Boras, R. D. Cardin, A. Carlo, K. J. Coffman, A. Dantonio, L. Di, H. Eng, R. Ferre, K. S. Gajiwala, S. A. Gibson, S. E. Greasley, B. L. Hurst, E. P. Kadar, A. S. Kalgutkar, J. C. Lee, J. Lee, W. Liu, S. W. Mason, S. Noell, J. J. Novak, R. S. Obach, K. Ogilvie, N. C. Patel, M. Pettersson, D. K. Rai, M. R. Reese, M. F. Sammons, J. G. Sathish, R. S. P. Singh, C. M. Steppan, A. E. Stewart, J. B. Tuttle, L. Updyke, P. R. Verhoest, L. Wei, Q. Yang and Y. Zhu, *Science*, 2021, **374**, 1586–1593.
10. T. R. Malla, L. Brewitz, D.-G. Muntean, H. Aslam, C. D. Owen, E. Salah, A. Tumber, P. Lukacik, C. Strain-Damerell, H. Mikolajek, M. A. Walsh and C. J. Schofield, *J. Med. Chem.*, 2022, **65**, 7682–7696.
11. L. Brewitz, L. Dumjahn, Y. Zhao, C. D. Owen, S. M. Laidlaw, T. R. Malla, D. Nguyen, P. Lukacik, E. Salah, A. D. Crawshaw, A. J. Warren, J. Trincao, C. Strain-Damerell, M. W. Carroll, M. A. Walsh and C. J. Schofield, *J. Med. Chem.*, 2023, **66**, 2663–2680.
12. S. B. Rosenblum, T. Huynh, A. Afonso, H. R. Davis, N. Yumibe, J. W. Clader and D. A. Burnett, *J. Med. Chem.*, 1998, **41**, 973–980.
13. C. Zhou, X. Hao, Z. Chen, R. Zhang, Q. Zhou, Z. Fan, M. Zheng, H. Hou, S. Zhang and H. Guo, *ACS Med. Chem. Lett.*, 2024, **15**, 270–279.
14. G. M. Sastry, M. Adzhigirey, T. Day, R. Annabhimoju and W. Sherman, *J. Comput. Aided Mol. Des.*, 2013, **27**, 221–234.
15. L. Schrödinger, Maestro, 2023.
16. R. A. Friesner, R. B. Murphy, M. P. Repasky, L. L. Frye, J. R. Greenwood, T. A. Halgren, P. C. Sanschagrin and D. T. Mainz, *J. Med. Chem.*, 2006, **49**, 6177–6196.
17. J. Wang, R. M. Wolf, J. W. Caldwell, P. A. Kollman and D. A. Case, *J. Comput. Chem.*, 2004, **25**, 1157–1174.
18. J. Wang, W. Wang, P. A. Kollman and D. A. Case, *J. Mol. Graphics Modell.*, 2006, **25**, 247–260.
19. C. I. Bayly, P. Cieplak, W. Cornell and P. A. Kollman, *J. Phys. Chem.*, 1993, **97**, 10269–10280.
20. M. J. Frisch, G. W. Trucks, H. B. Schlegel, G. E. Scuseria, M. A. Robb, J. R. Cheeseman, G. Scalmani, V. Barone, G. A. Petersson, H. Nakatsuji, X. Li, M. Caricato, A. V. Marenich, J. Bloino, B. G. Janesko, R. Gomperts, B. Mennucci, H. P. Hratchian, J. V. Ortiz, A. F. Izmaylov, J. L. Sonnenberg, Williams, F. Ding, F. Lipparini, F. Egidi, J. Goings, B. Peng, A. Petrone, T. Henderson, D. Ranasinghe, V. G. Zakrzewski, J. Gao, N. Rega, G. Zheng, W. Liang, M. Hada, M. Ehara, K. Toyota, R. Fukuda, J. Hasegawa, M. Ishida, T. Nakajima, Y. Honda, O. Kitao, H. Nakai, T. Vreven, K. Throssell, J. A. Montgomery Jr., J. E. Peralta, F. Ogliaro, M. J. Bearpark, J. J. Heyd, E. N. Brothers, K. N. Kudin, V. N. Staroverov, T. A. Keith, R. Kobayashi, J. Normand,

K. Raghavachari, A. P. Rendell, J. C. Burant, S. S. Iyengar, J. Tomasi, M. Cossi, J. M. Millam, M. Klene, C. Adamo, R. Cammi, J. W. Ochterski, R. L. Martin, K. Morokuma, O. Farkas, J. B. Foresman and D. J. Fox, Gaussian 16 Rev. C.01, 2016.

21. H. M. A. D.A. Case, K. Belfon, I.Y. Ben-Shalom, J.T. Berryman, S.R. Brozell, D.S. Cerutti, T.E. Cheatham, III, G.A. Cisneros, V.W.D. Cruzeiro, T.A. Darden, N. Forouzesh, M. Ghazimirsaeed, G. Giambaşu, T. Giese, M.K. Gilson, H. Gohlke, A.W. Goetz, J. Harris, Z. Huang, S. Izadi, S.A. Izmailov, K. Kasavajhala, M.C. Kaymak, A. Kovalenko, T. Kurtzman, T.S. Lee, P. Li, Z. Li, C. Lin, J. Liu, T. Luchko, R. Luo, M. Machado, M. Manathunga, K.M. Merz, Y. Miao, O. Mikhailovskii, G. Monard, H. Nguyen, K.A. O'Hearn, A. Onufriev, F. Pan, S. Pantano, A. Rahnamoun, D.R. Roe, A. Roitberg, C. Sagui, S. Schott-Verdugo, A. Shajan, J. Shen, C.L. Simmerling, N.R. Skrynnikov, J. Smith, J. Swails, R.C. Walker, J. Wang, J. Wang, X. Wu, Y. Wu, Y. Xiong, Y. Xue, D.M. York, C. Zhao, Q. Zhu, and P.A. Kollman, Amber 2024, 2024.
22. D. A. Case, H. M. Aktulga, K. Belfon, D. S. Cerutti, G. A. Cisneros, V. W. D. Cruzeiro, N. Forouzesh, T. J. Giese, A. W. Götz, H. Gohlke, S. Izadi, K. Kasavajhala, M. C. Kaymak, E. King, T. Kurtzman, T.-S. Lee, P. Li, J. Liu, T. Luchko, R. Luo, M. Manathunga, M. R. Machado, H. M. Nguyen, K. A. O'Hearn, A. V. Onufriev, F. Pan, S. Pantano, R. Qi, A. Rahnamoun, A. Risheh, S. Schott-Verdugo, A. Shajan, J. Swails, J. Wang, H. Wei, X. Wu, Y. Wu, S. Zhang, S. Zhao, Q. Zhu, T. E. Cheatham, III, D. R. Roe, A. Roitberg, C. Simmerling, D. M. York, M. C. Nagan and K. M. Merz, Jr., *J. Chem. Inf. Model.*, 2023, **63**, 6183–6191.
23. W. L. Jorgensen, J. Chandrasekhar, J. D. Madura, R. W. Impey and M. L. Klein, *J. Chem. Phys.*, 1983, **79**, 926–935.
24. H. J. C. Berendsen, J. P. M. Postma, W. F. van Gunsteren, A. DiNola and J. R. Haak, *J. Chem. Phys.*, 1984, **81**, 3684–3690.
25. J.-P. Ryckaert, G. Ciccotti and H. J. C. Berendsen, *J. Comput. Phys.*, 1977, **23**, 327–341.
26. J. A. Maier, C. Martinez, K. Kasavajhala, L. Wickstrom, K. E. Hauser and C. Simmerling, *J. Chem. Theory Comput.*, 2015, **11**, 3696–3713.
27. D. R. Roe and T. E. Cheatham, III, *J. Chem. Theory Comput.*, 2013, **9**, 3084–3095.
28. *CA Pat.,* CA 1040620 A, 1978.
29. H. Kamachi, T. Okita, T. Yamasaki and T. Naito, *J. Antibiot.*, 1990, **43**, 820–829.
30. A. W. Chow, N. M. Hall and J. R. E. Hoover, *J. Org. Chem.*, 1962, **27**, 1381–1383.
31. J. E. Baldwin, F. J. Urban, R. D. G. Cooper and F. L. Jose, *J. Am. Chem. Soc.*, 1973, **95**, 2401–2403.
32. M. Benz, T. M. Klapötke, J. Stierstorfer and M. Voggenreiter, *J. Am. Chem. Soc.*, 2022, **144**, 6143–6147.
33. P. J. Giddings, D. I. John, E. J. Thomas and D. J. Williams, *J. Chem. Soc., Perkin Trans. 1*, 1982, 2757–2766.
34. B. M. Sadeghpour, R. Pellicciari, C. Marchioro, T. Rossi, B. Tamburini and G. Tarzia, *Tetrahedron*, 1995, **51**, 10723–10730.
35. Y. S. Lo and J. C. Sheehan, *J. Org. Chem.*, 1975, **40**, 191–192.
36. T. Jen, J. Frazee and J. R. E. Hoover, *J. Org. Chem.*, 1973, **38**, 2857–2859.
37. J. A. Aimetti, E. S. Hamanaka, D. A. Johnson and M. S. Kellogg, *Tetrahedron Lett.*, 1979, **20**, 4631–4634.
38. *US Pat.,* US 4847247 A, 1989.

7. ^{1}H and ^{13}C NMR spectra of novel C6-alkoxy penicillin derivatives prepared for this study

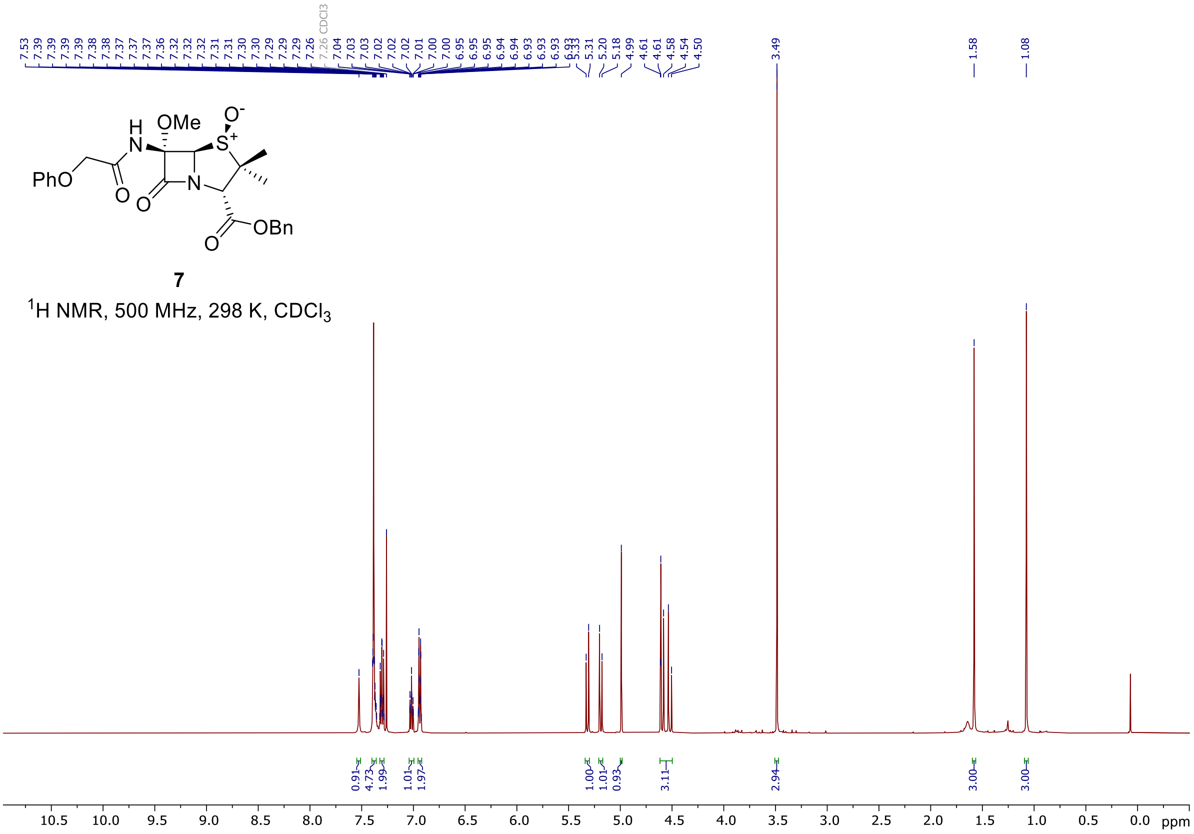

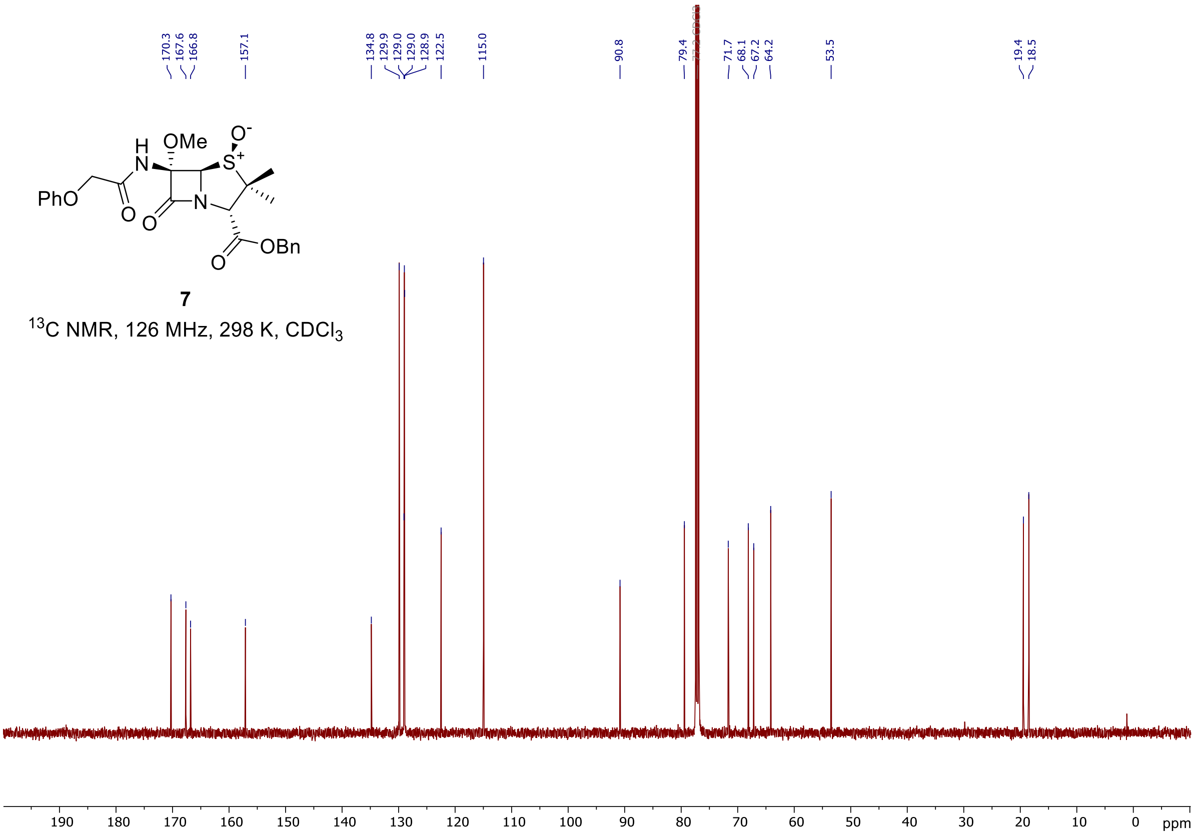

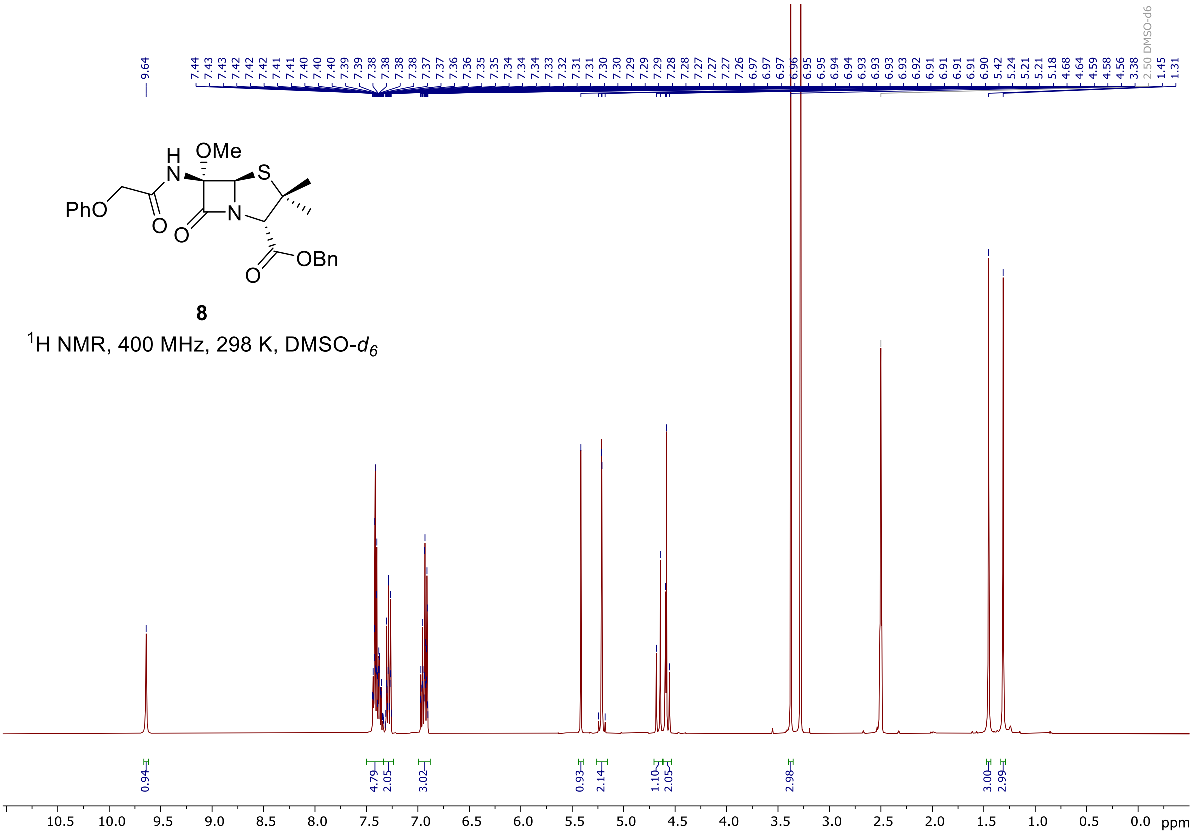

8

^{1}H NMR, 400 MHz, 298 K, DMSO-d_6

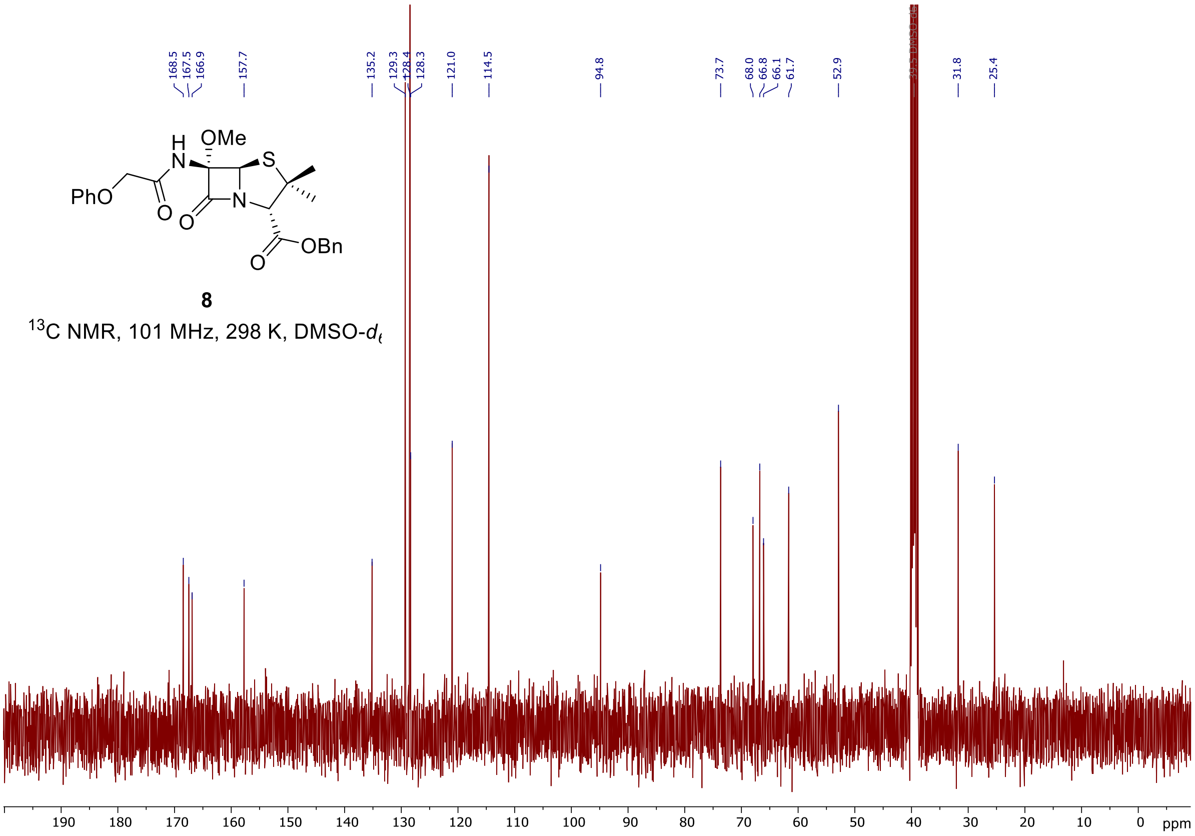

8

^{13}C NMR, 101 MHz, 298 K, DMSO-d_6

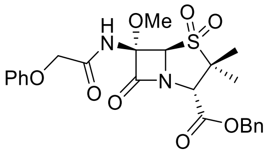

9

^{1}H NMR, 600 MHz, 298 K, DMSO-d_6

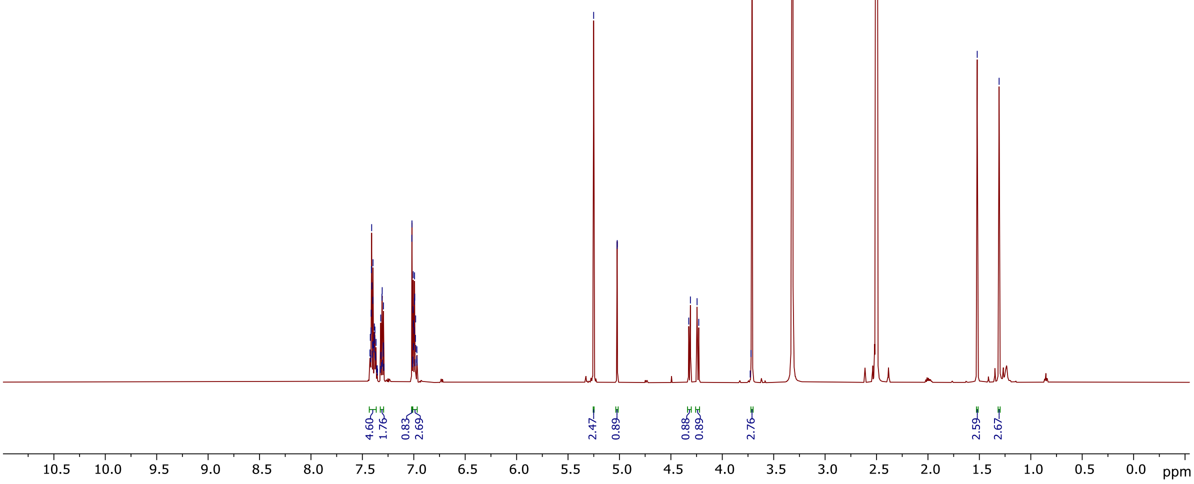

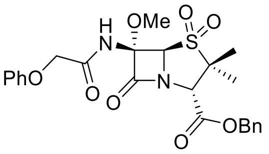

9

^{13}C NMR, 151 MHz, 298 K, DMSO-d_6

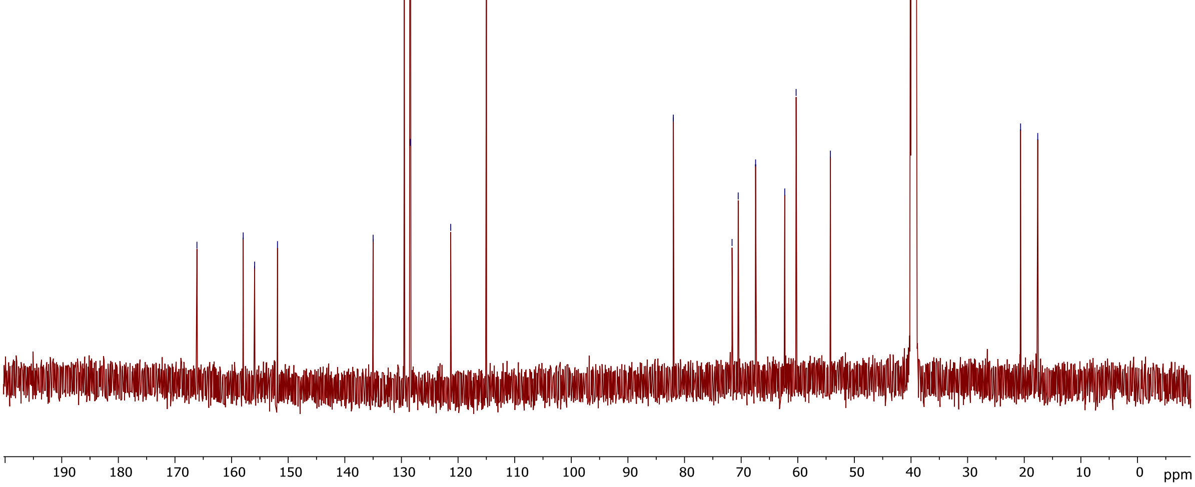

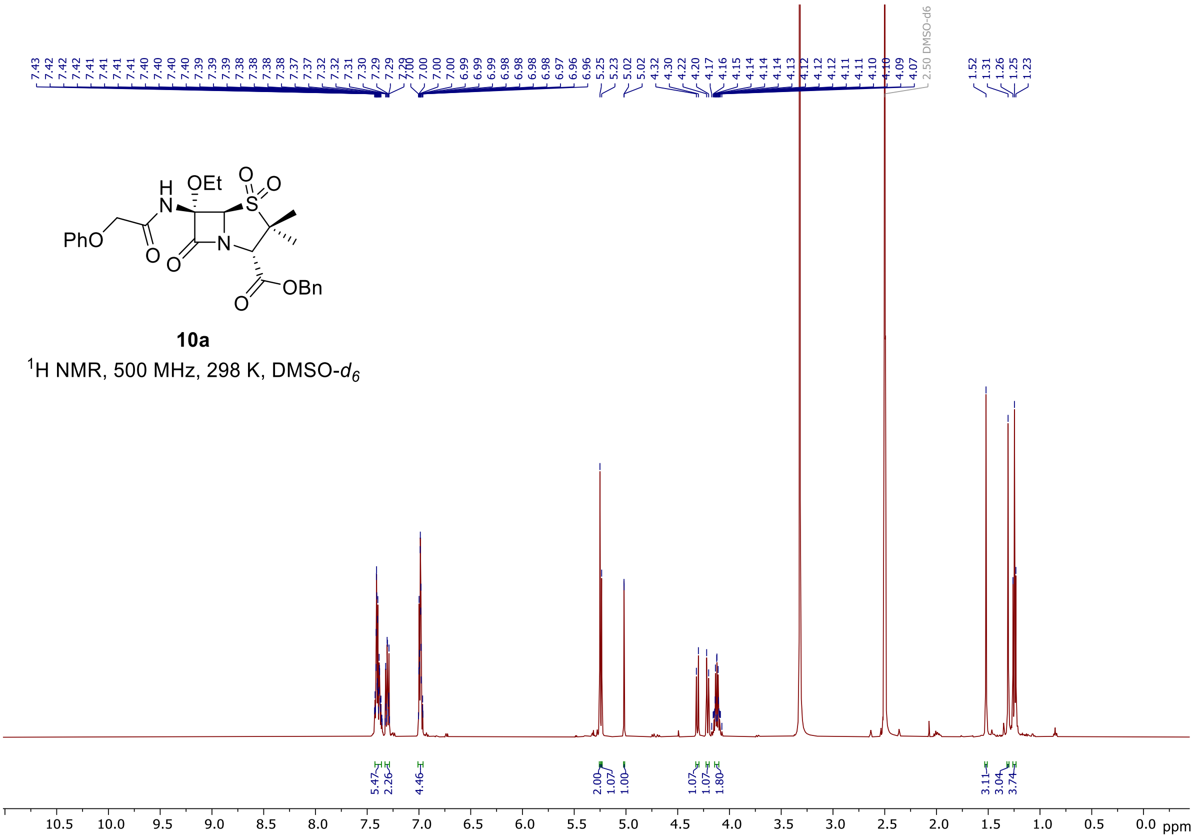
10a
^{1}H NMR, 500 MHz, 298 K, DMSO-d_6
ppm

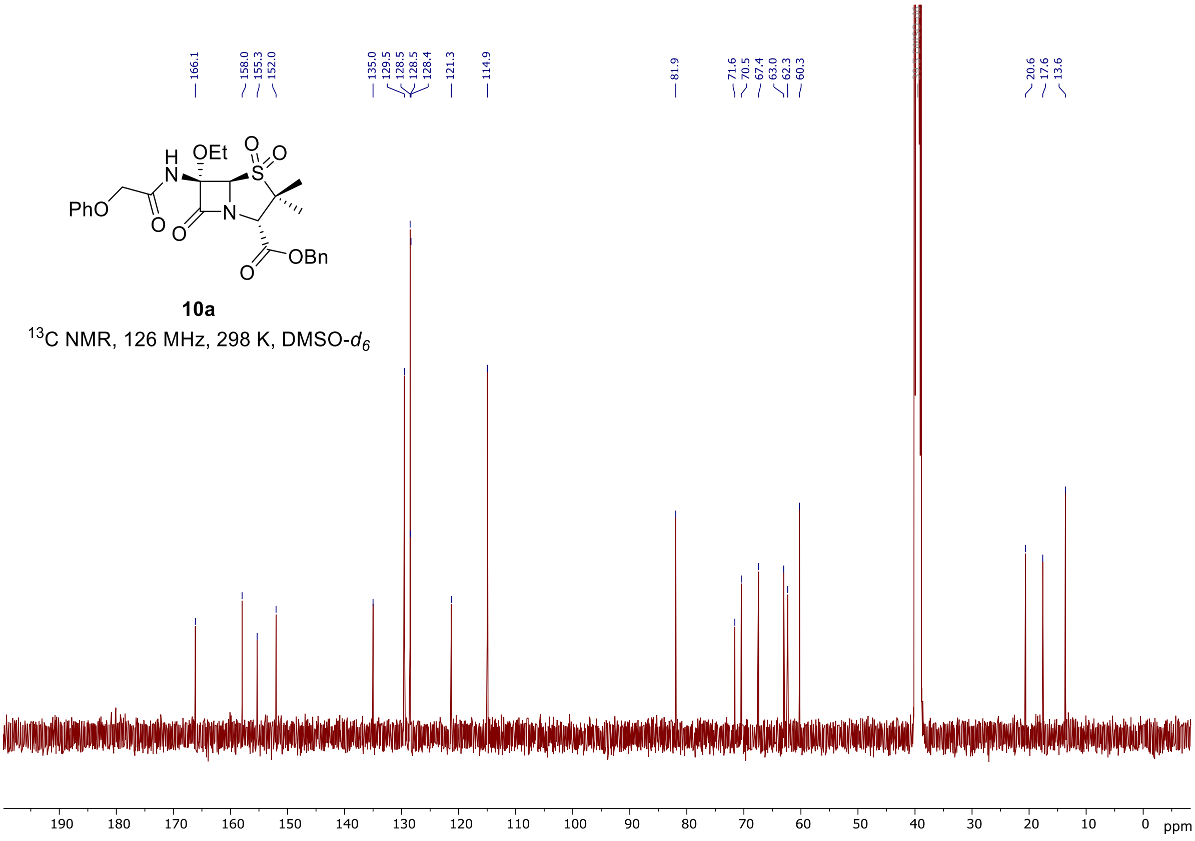
10a
^{13}C NMR, 126 MHz, 298 K, DMSO-d_6
ppm

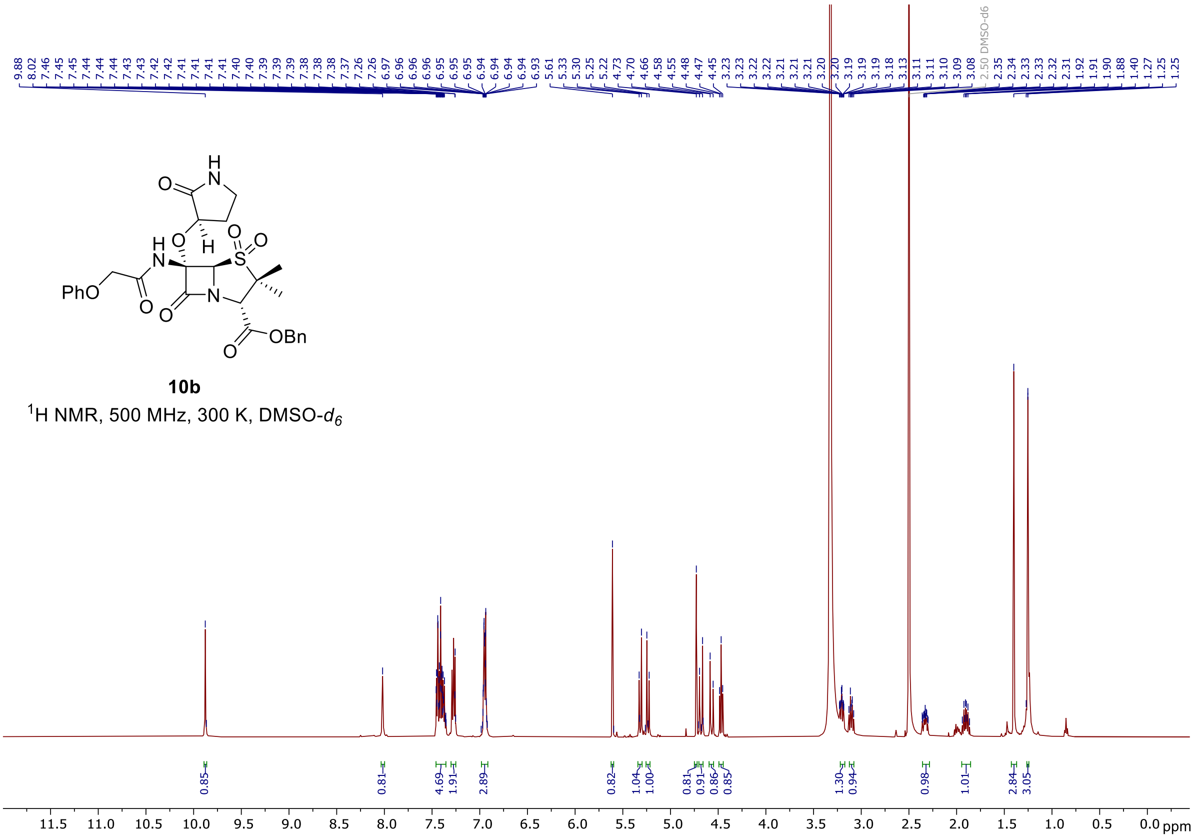

10b

^{1}H NMR, 500 MHz, 300 K, DMSO-d_6

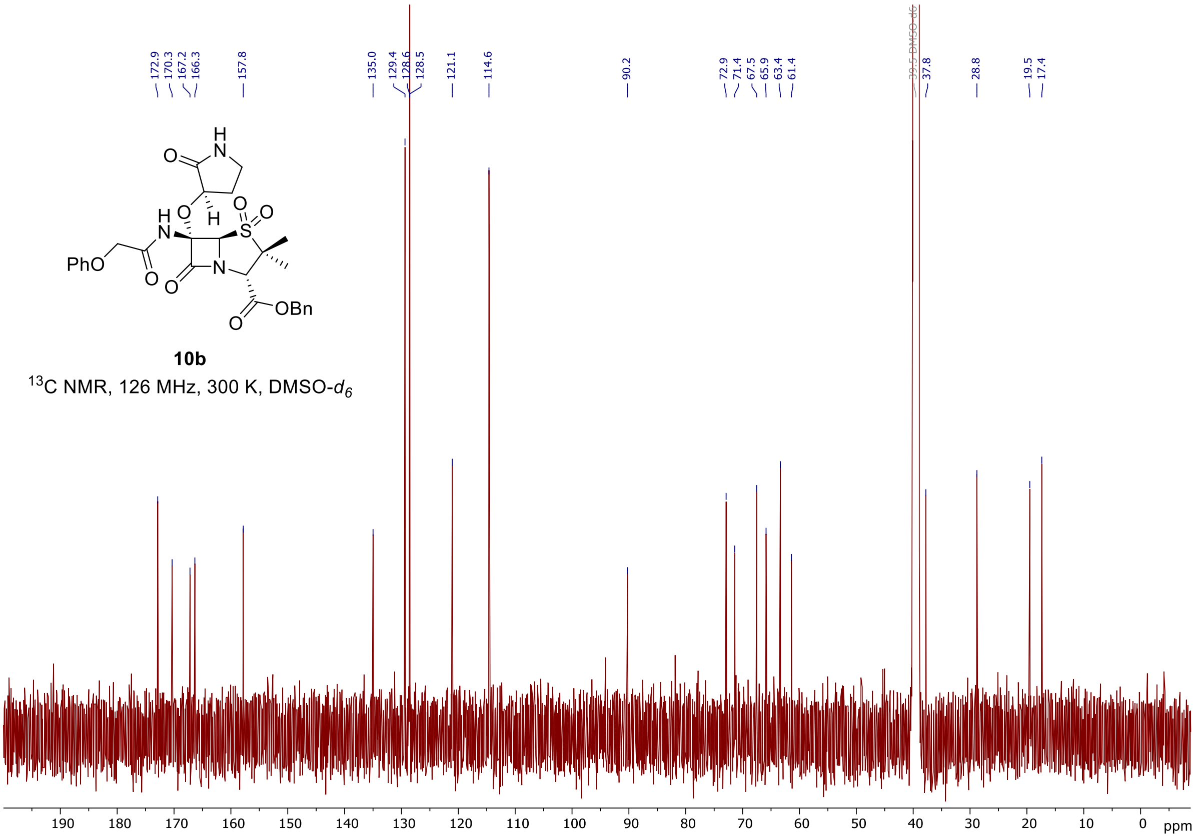

10b

^{13}C NMR, 126 MHz, 300 K, DMSO-d_6

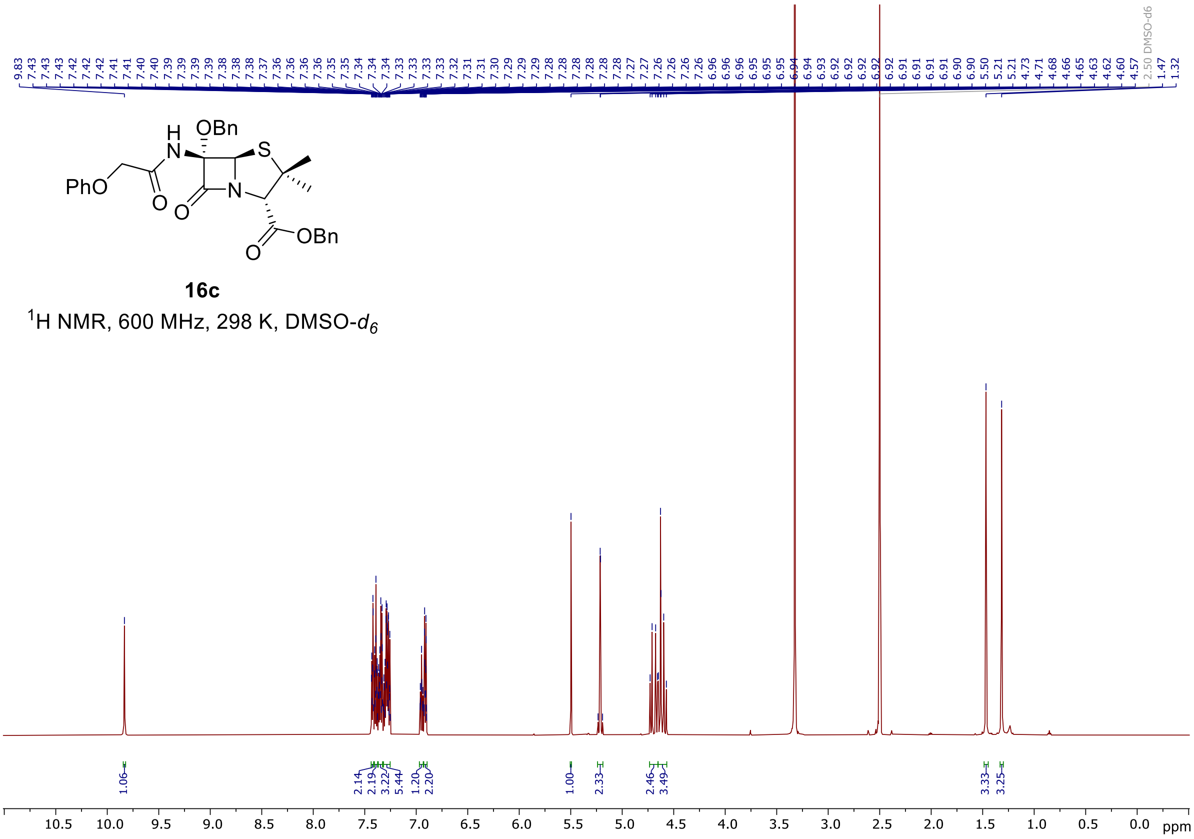

16c

^{1}H NMR, 600 MHz, 298 K, DMSO-d_6

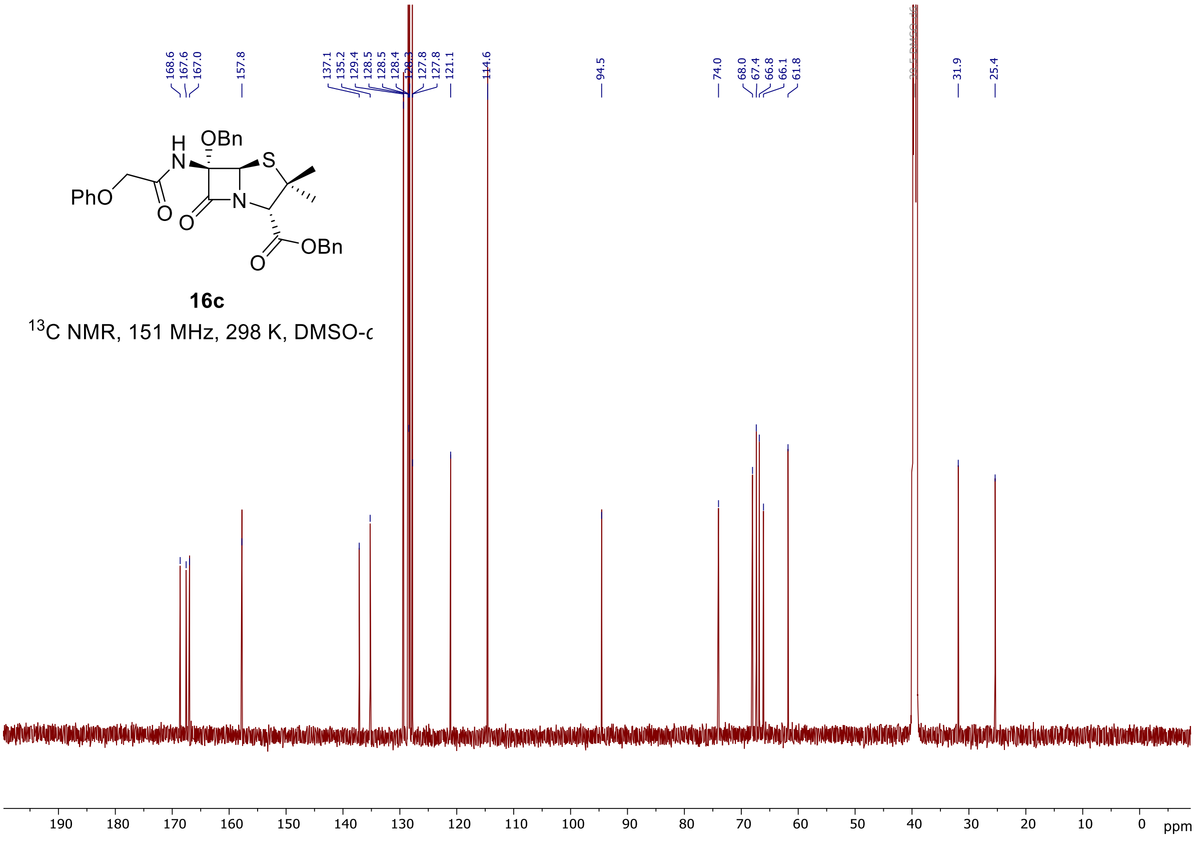

16c

^{13}C NMR, 151 MHz, 298 K, DMSO-[illegible]

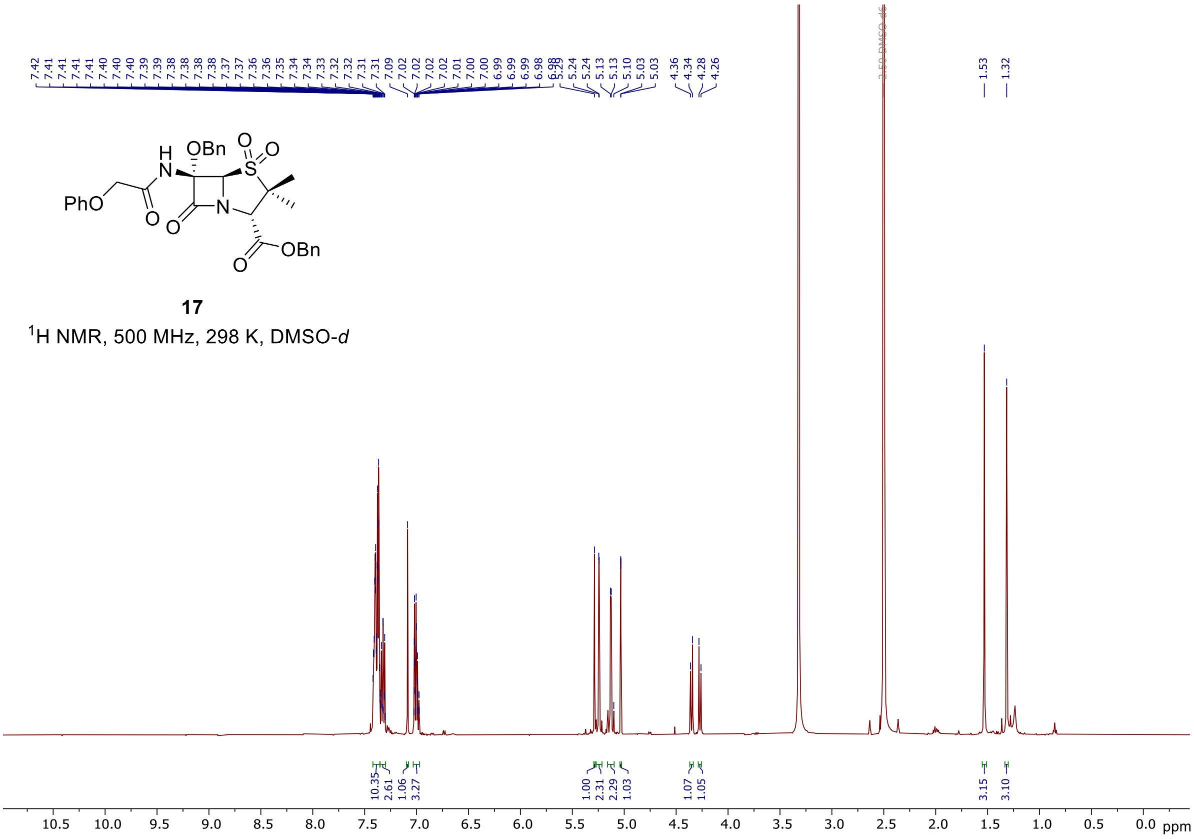

17

^{1}H NMR, 500 MHz, 298 K, DMSO-*d*

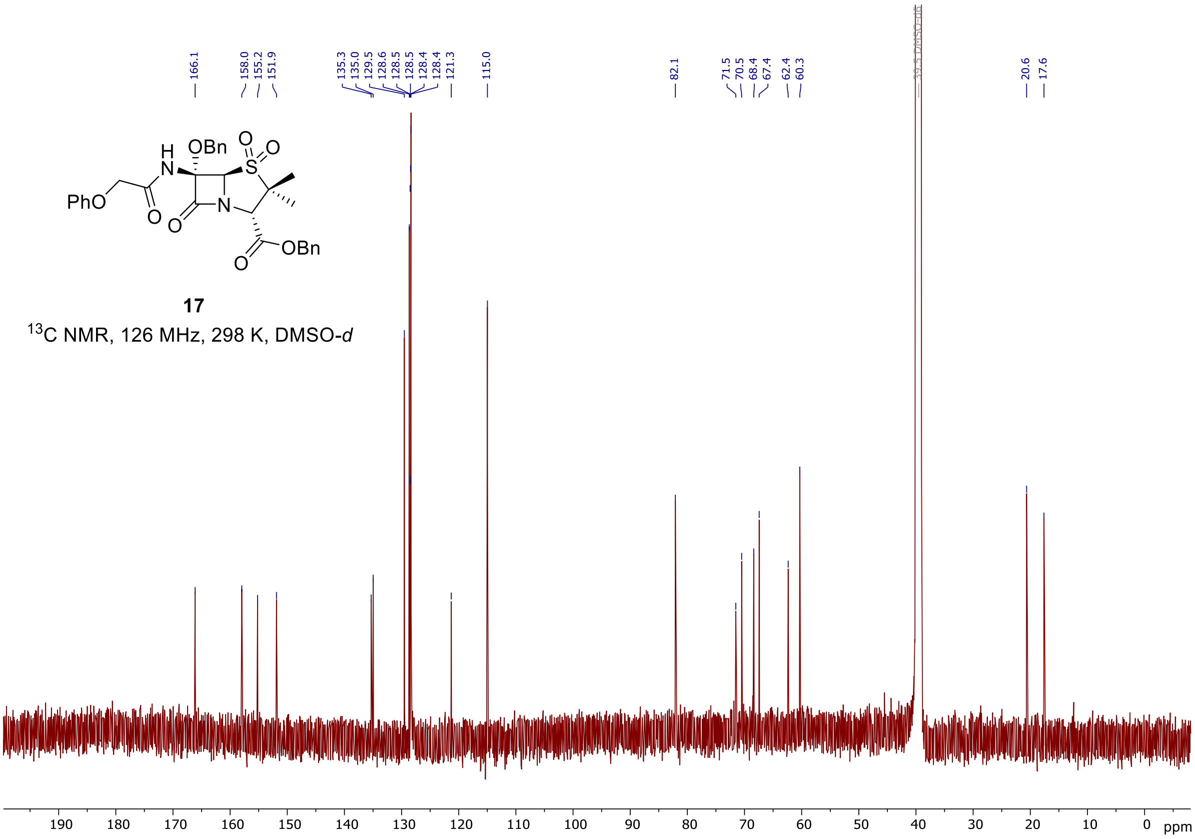

17

^{13}C NMR, 126 MHz, 298 K, DMSO-*d*

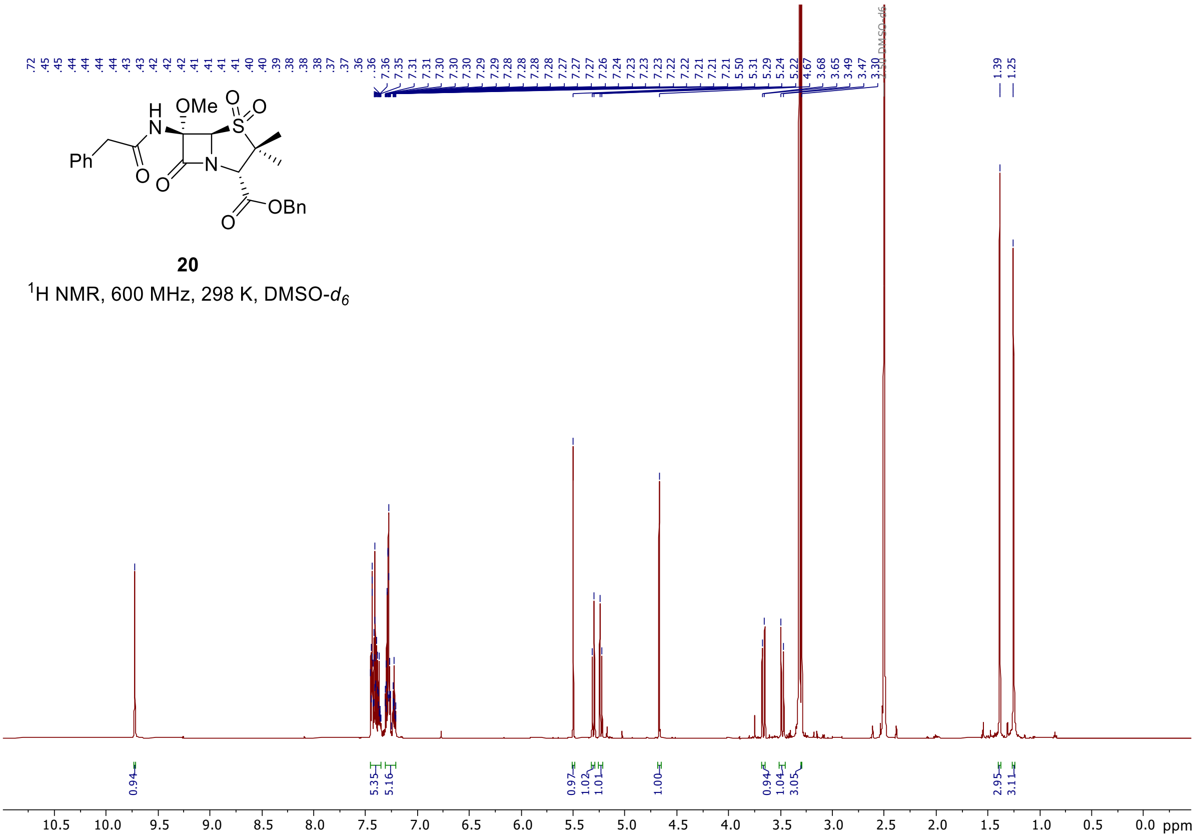

20

^{1}H NMR, 600 MHz, 298 K, DMSO-d_6

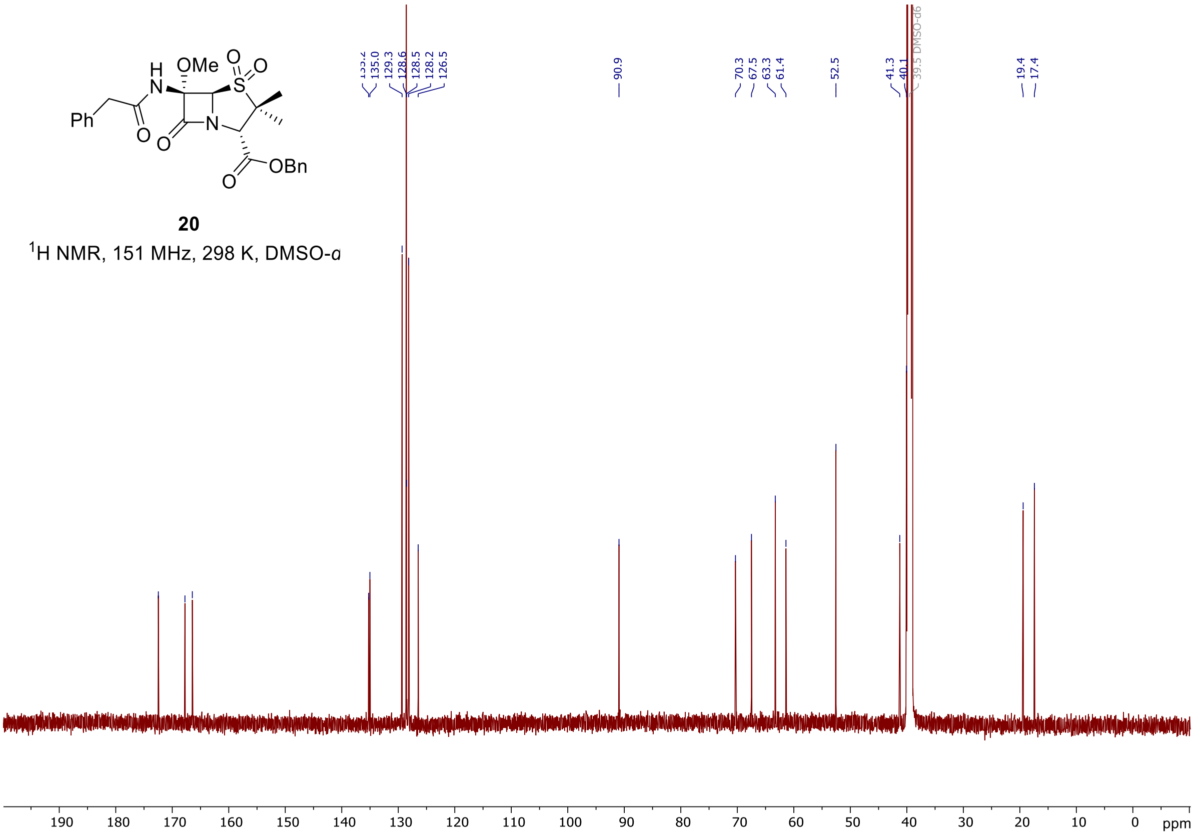

20

^{1}H NMR, 151 MHz, 298 K, DMSO-*d*

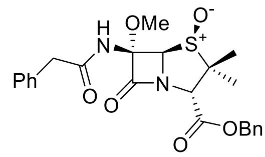

22

^{1}H NMR, 500 MHz, 298 K, DMSO-d_6

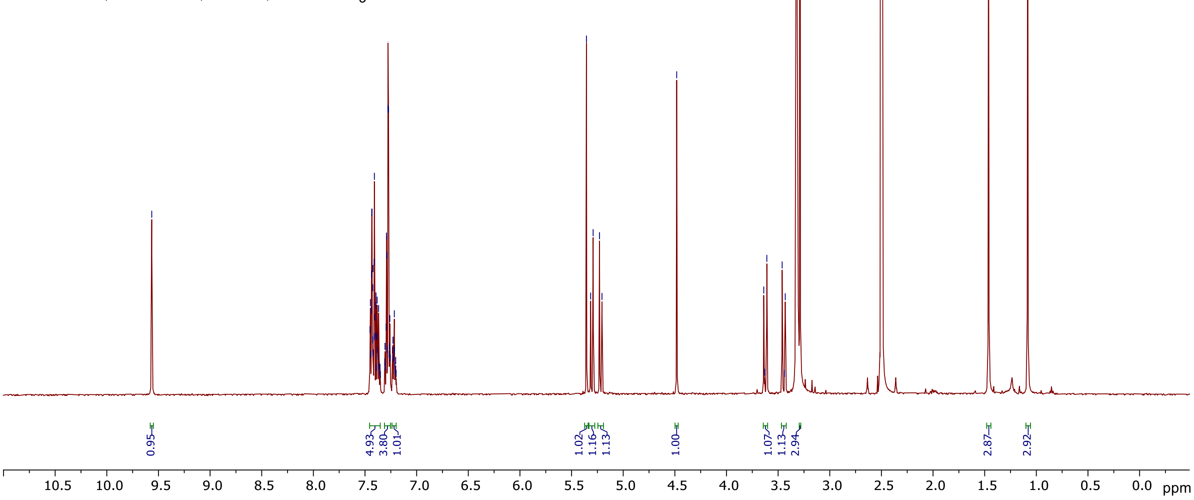

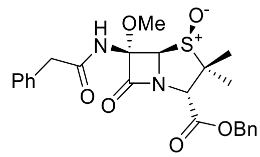

22

^{13}C NMR, 126 MHz, 298 K, DMSO-d_6

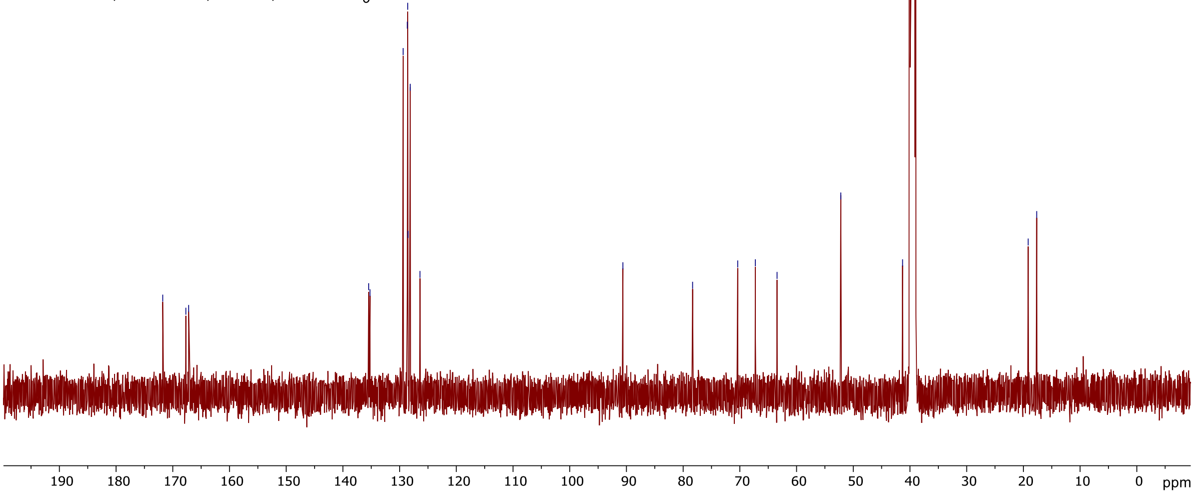

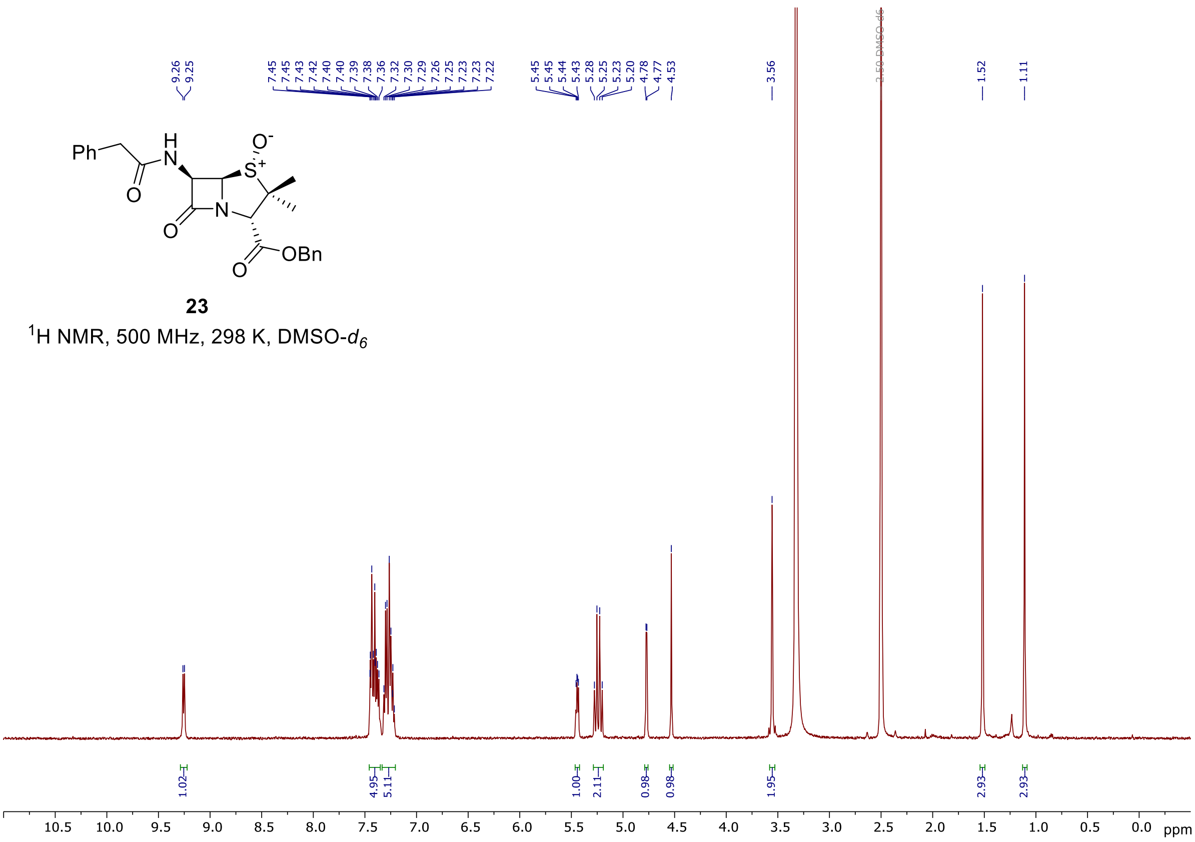

23

^{1}H NMR, 500 MHz, 298 K, DMSO-d_6

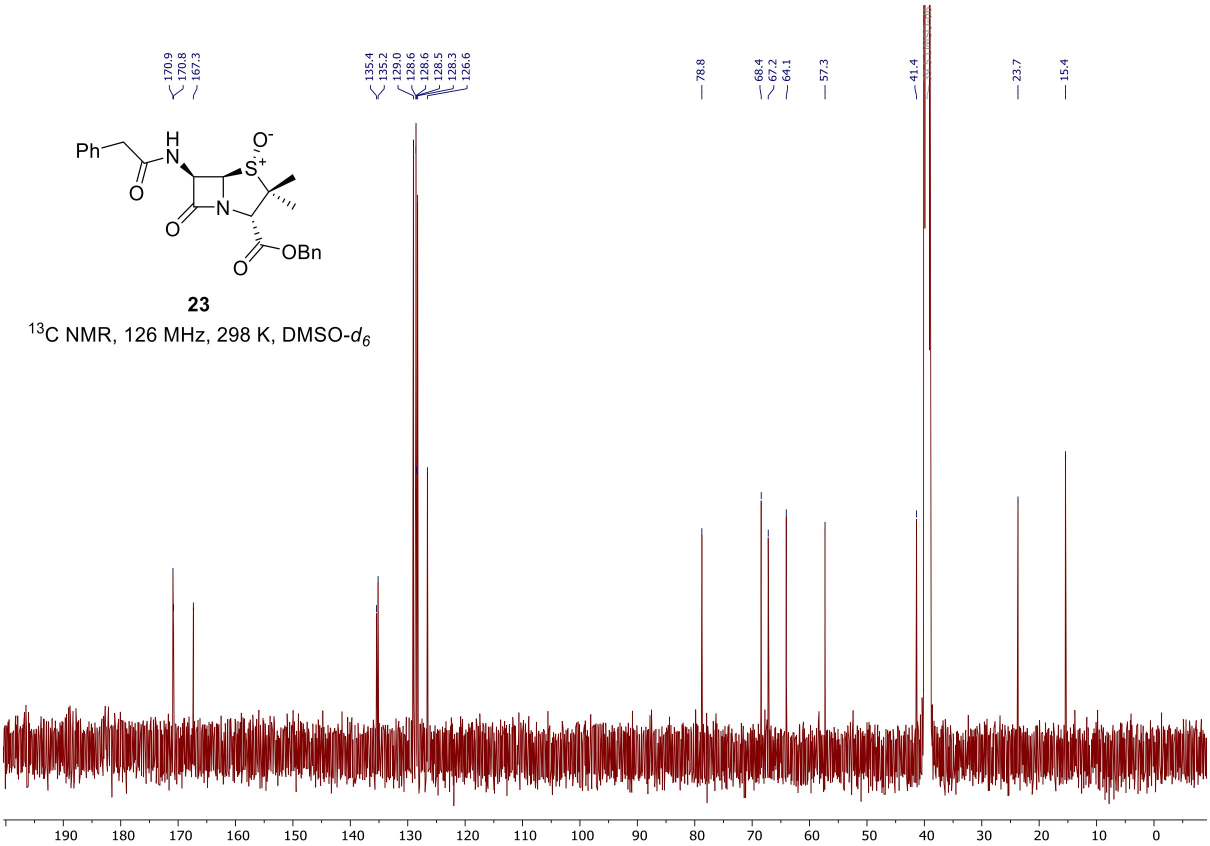

23

^{13}C NMR, 126 MHz, 298 K, DMSO-d_6

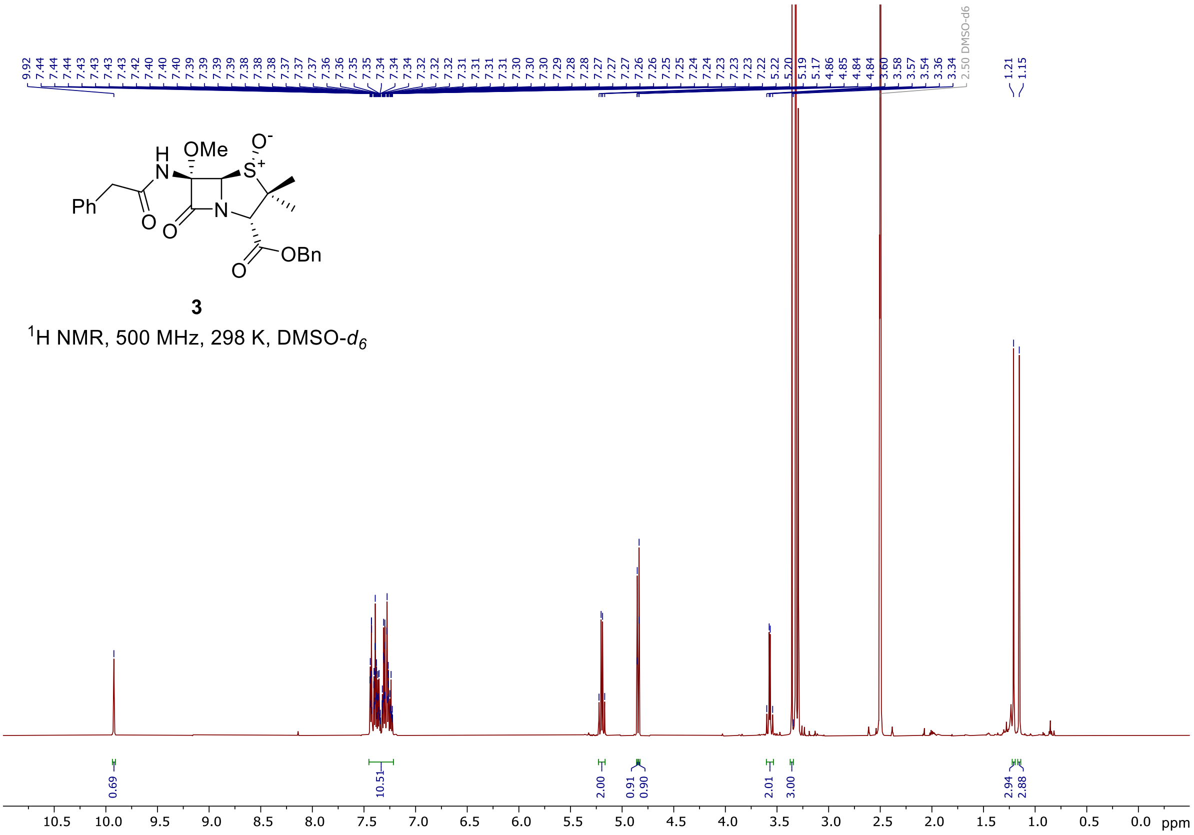

3

^{1}H NMR, 500 MHz, 298 K, DMSO-d_6

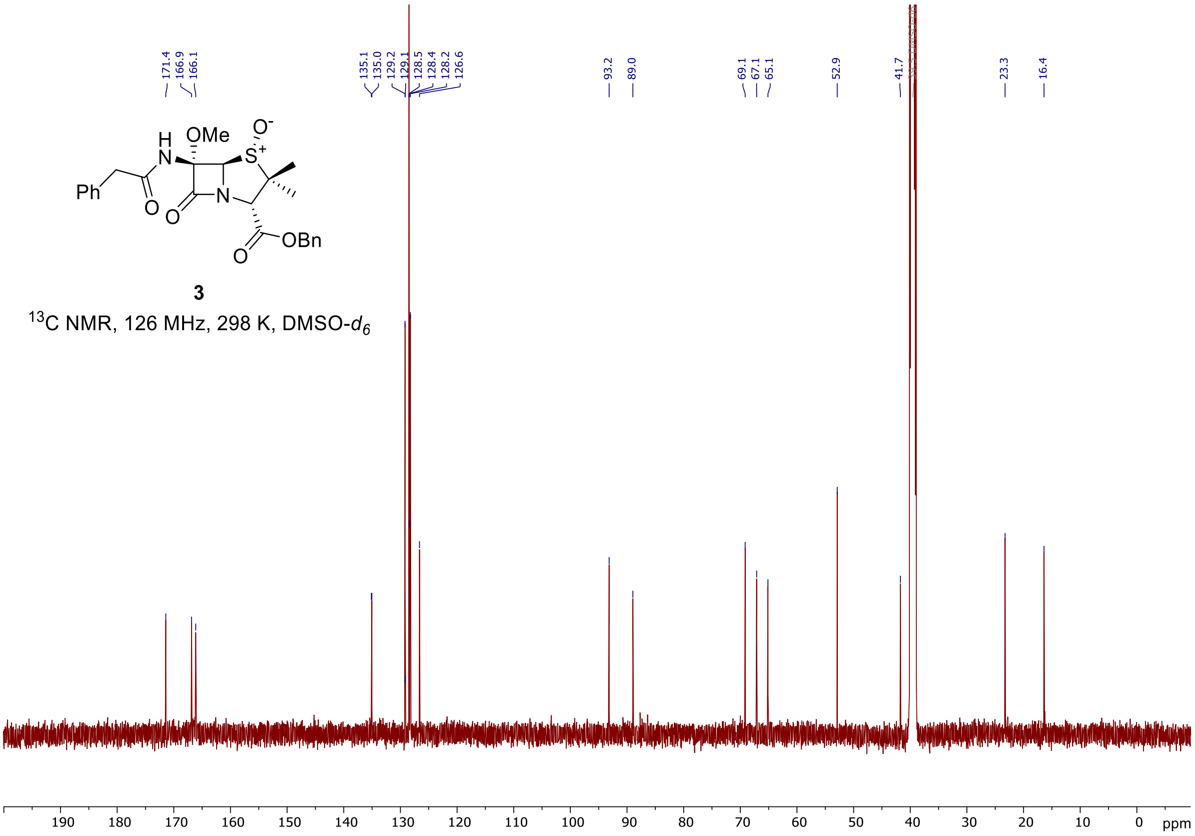

3

^{13}C NMR, 126 MHz, 298 K, DMSO-d_6

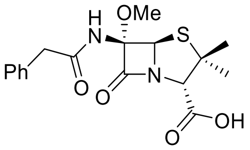

24

^{1}H NMR, 600 MHz, 298 K, DMSO-d_6

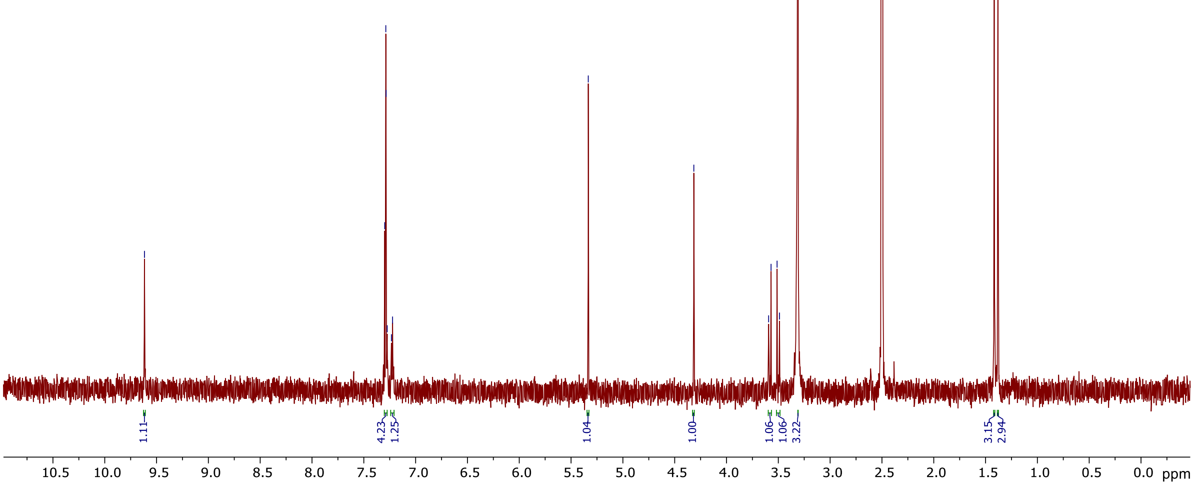

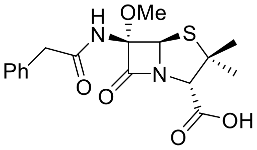

24

^{13}C NMR, 126 MHz, 298 K, DMSO-d_6

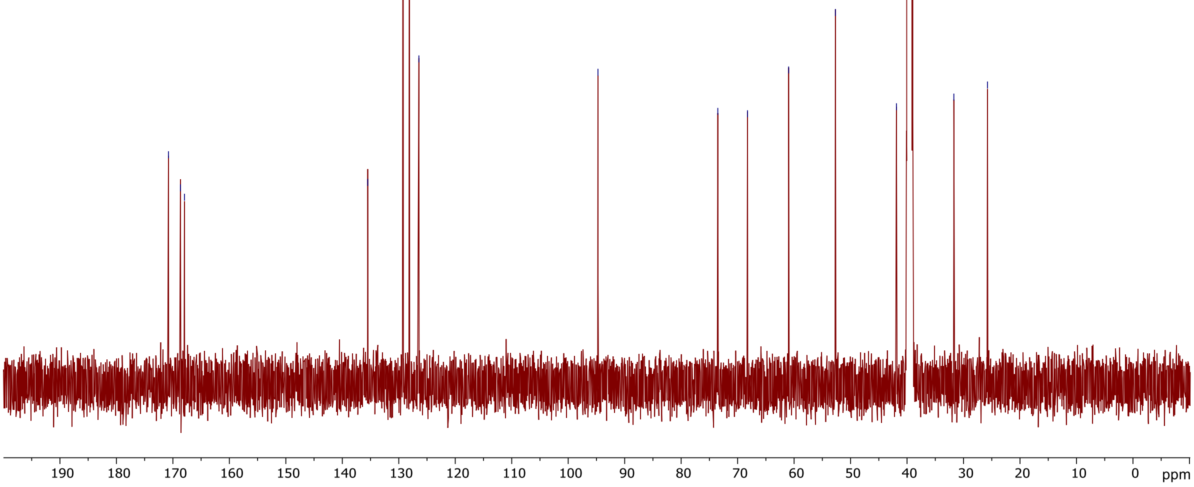

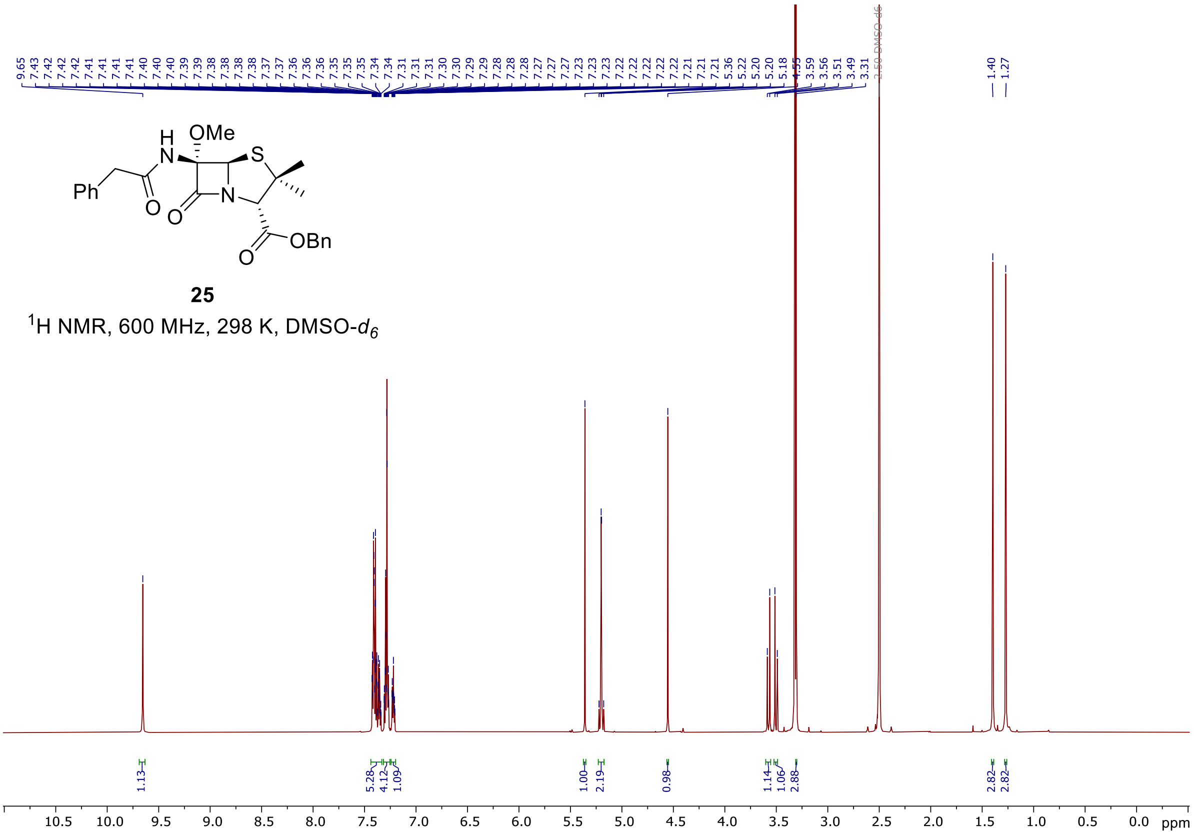
9.65 7.43 7.42 7.42 7.41 7.41 7.41 7.41 7.40 7.40 7.39 7.39 7.38 7.38 7.38 7.38 7.37 7.37 7.36 7.36 7.35 7.35 7.35 7.34 7.34 7.31 7.31 7.30 7.30 7.29 7.29 7.28 7.28 7.27 7.27 7.27 7.23 7.23 7.23 7.22 7.22 7.22 7.22 7.22 7.21 7.21 5.36 5.22 5.20 5.18 4.55 3.59 3.56 3.51 3.49 3.31 2.50 DMSO-d6
1.40
1.27
H
N
OMe
S
Ph
O
O
N
OBn
O
25
1H NMR, 600 MHz, 298 K, DMSO-d6
1.13
5.28
4.12
1.09
1.00
2.19
0.98
1.14
1.06
2.88
2.82
2.82
10.5 10.0 9.5 9.0 8.5 8.0 7.5 7.0 6.5 6.0 5.5 5.0 4.5 4.0 3.5 3.0 2.5 2.0 1.5 1.0 0.5 0.0 ppm

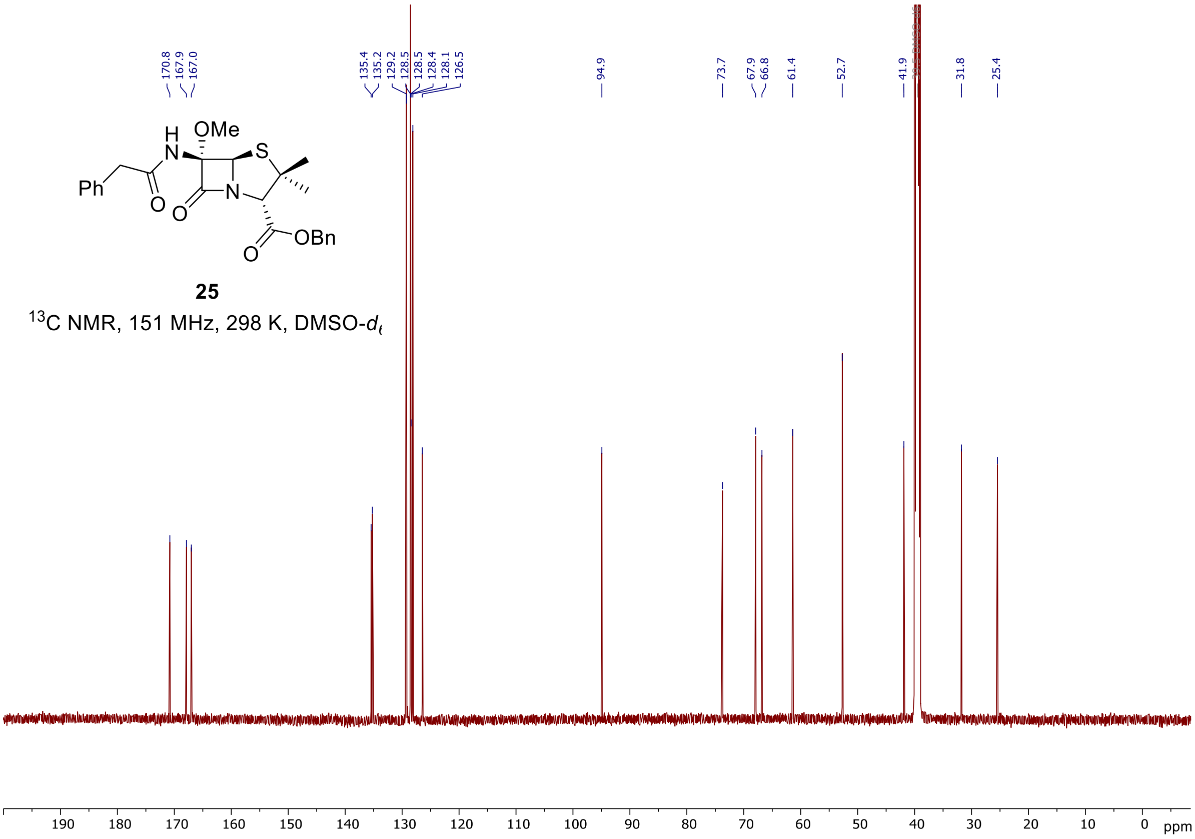
170.8 167.9 167.0
135.4 135.2 129.2 128.5 128.4 128.1 126.5
94.9
73.7
67.9 66.8
61.4
52.7
41.9
31.8
25.4
H
N
OMe
S
Ph
O
O
N
OBn
O
25
13C NMR, 151 MHz, 298 K, DMSO-d6
190 180 170 160 150 140 130 120 110 100 90 80 70 60 50 40 30 20 10 0 ppm

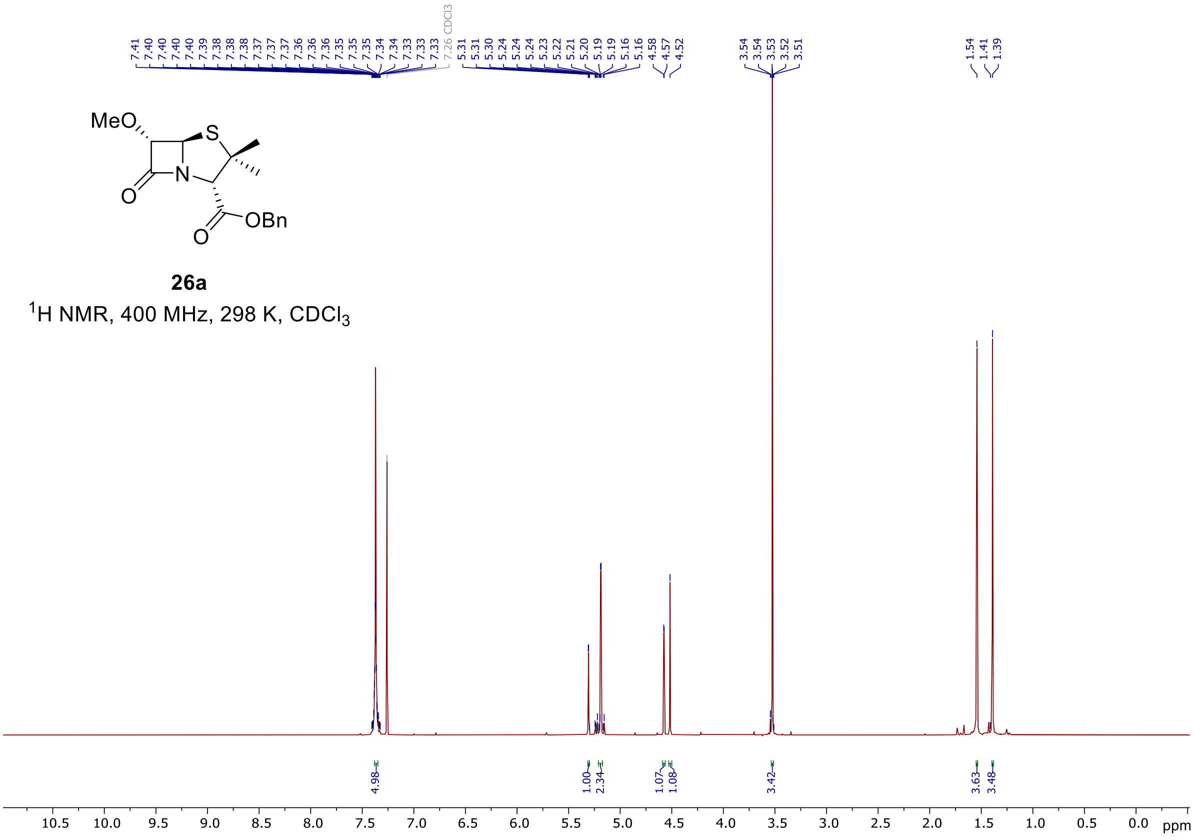

26a

^{1}H NMR, 400 MHz, 298 K, $CDCl_3$

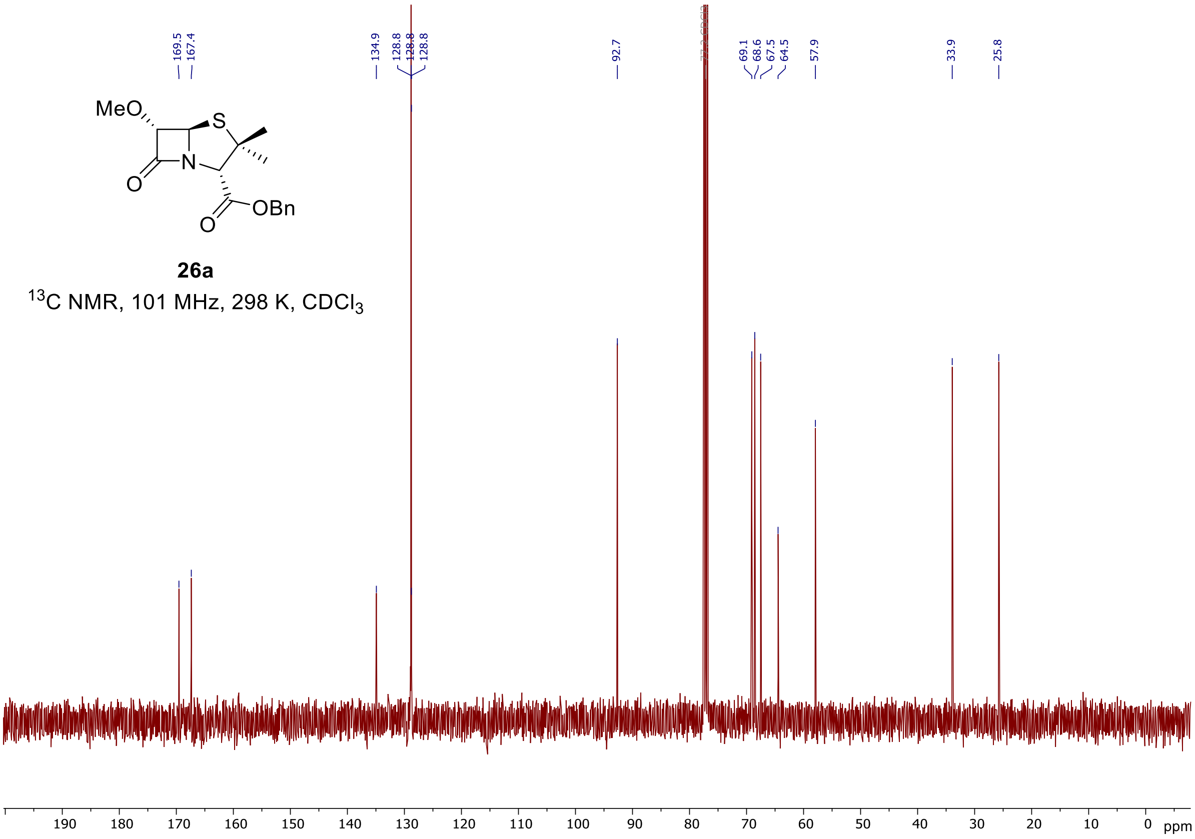

26a

^{13}C NMR, 101 MHz, 298 K, $CDCl_3$

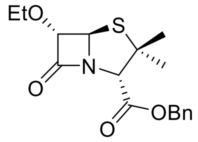

26b

^{1}H NMR, 500 MHz, 298 K, DMSO-d_6

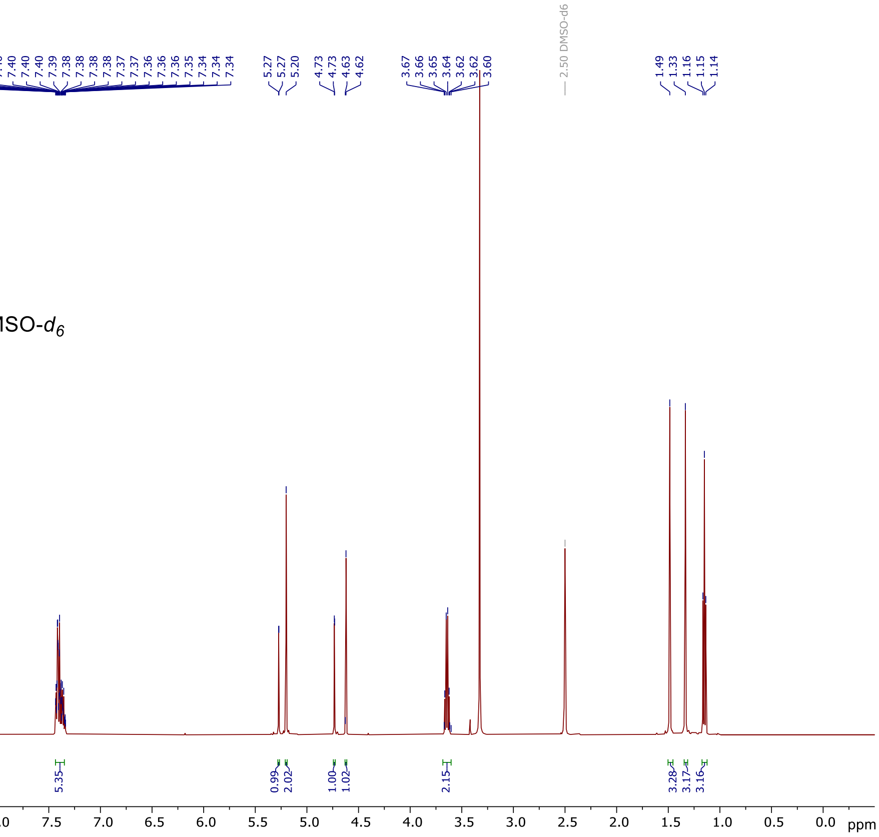

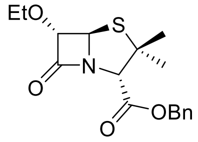

26b

^{13}C NMR, 126 MHz, 298 K, DMSO-d_6

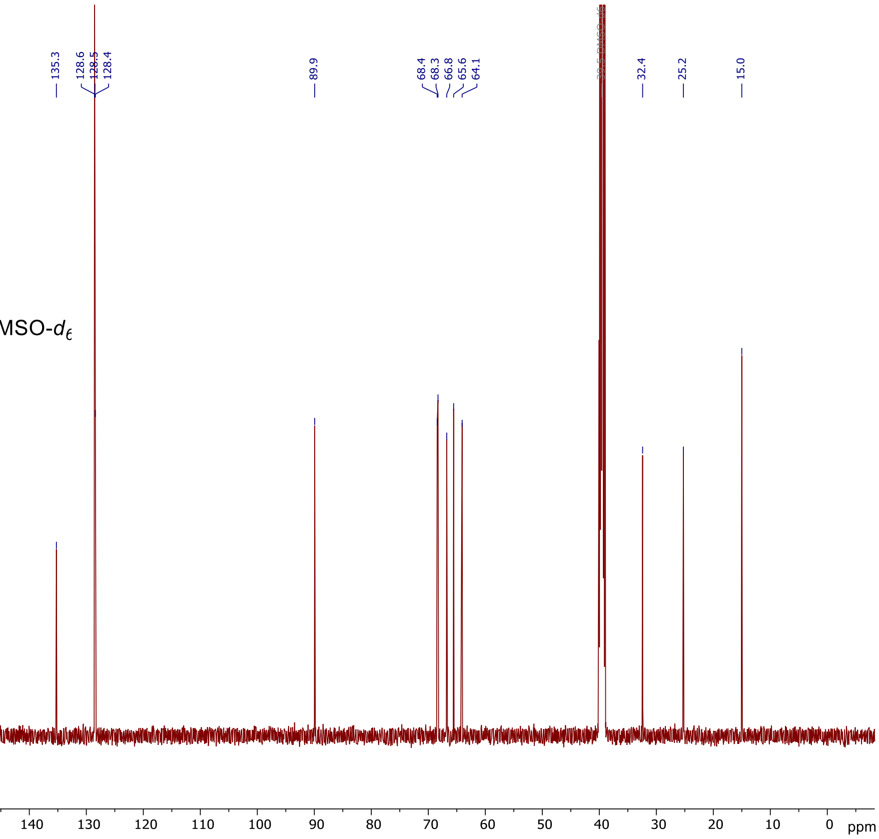

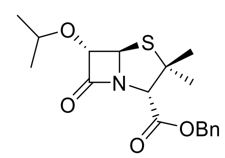

26c

^{1}H NMR, 600 MHz, 298 K, DMSO-d_6

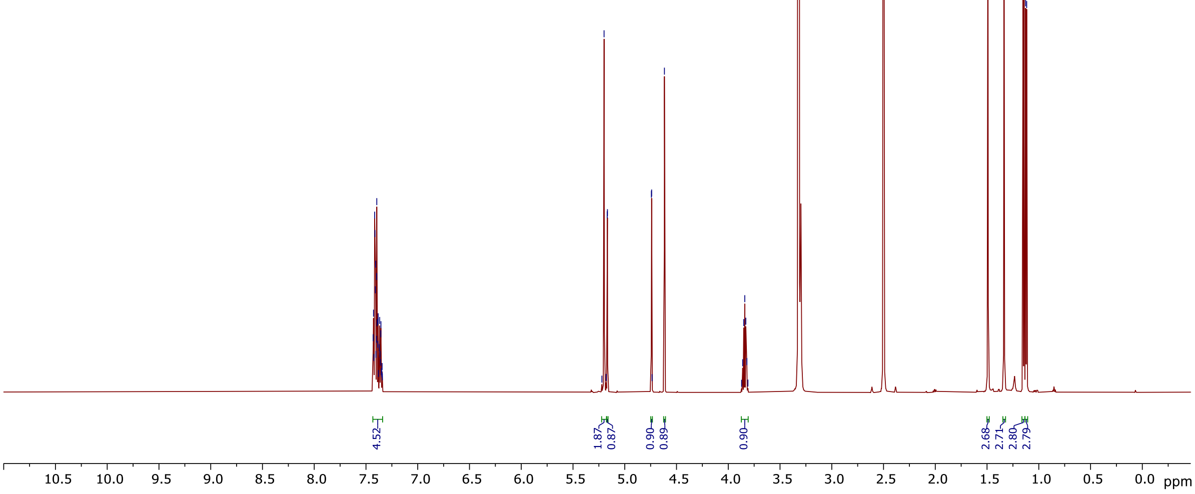

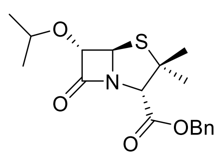

28c

^{13}C NMR, 151 MHz, 298 K, DMSO-d_6

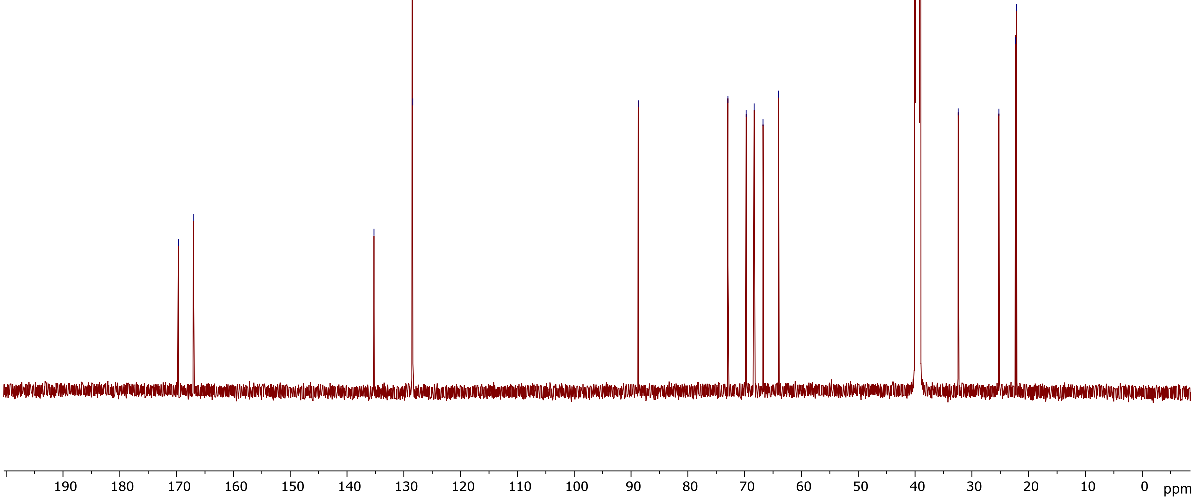

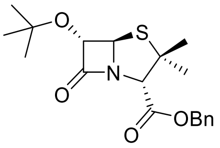

26d

^{1}H NMR, 500 MHz, 298 K, DMSO-d_6

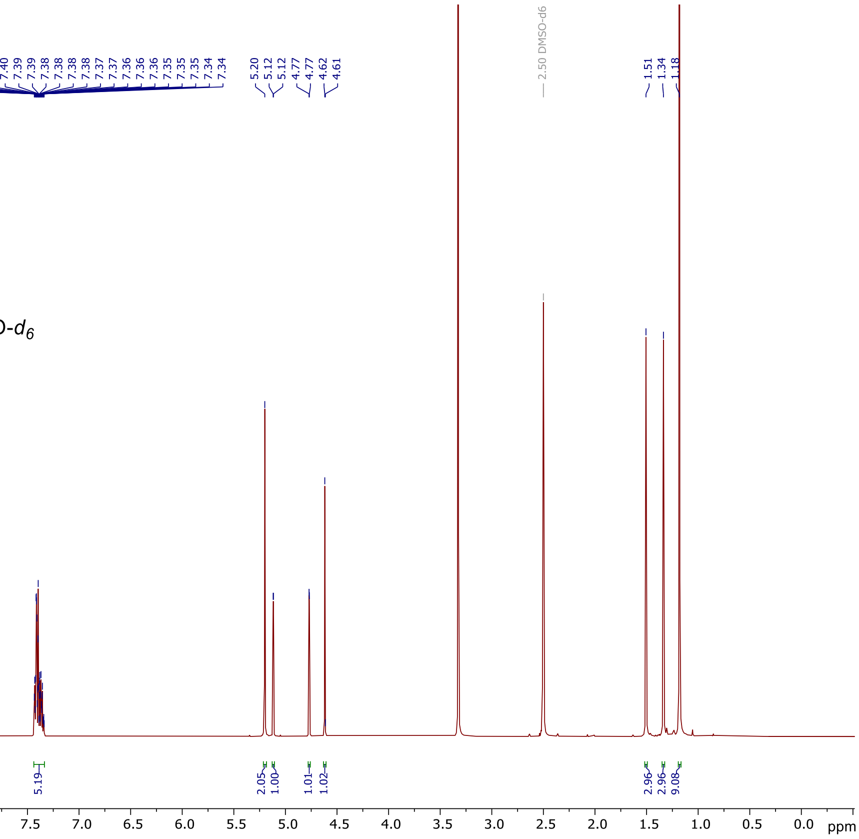

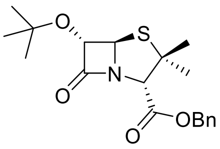

26d

^{13}C NMR, 126 MHz, 298 K, DMSO-d_6

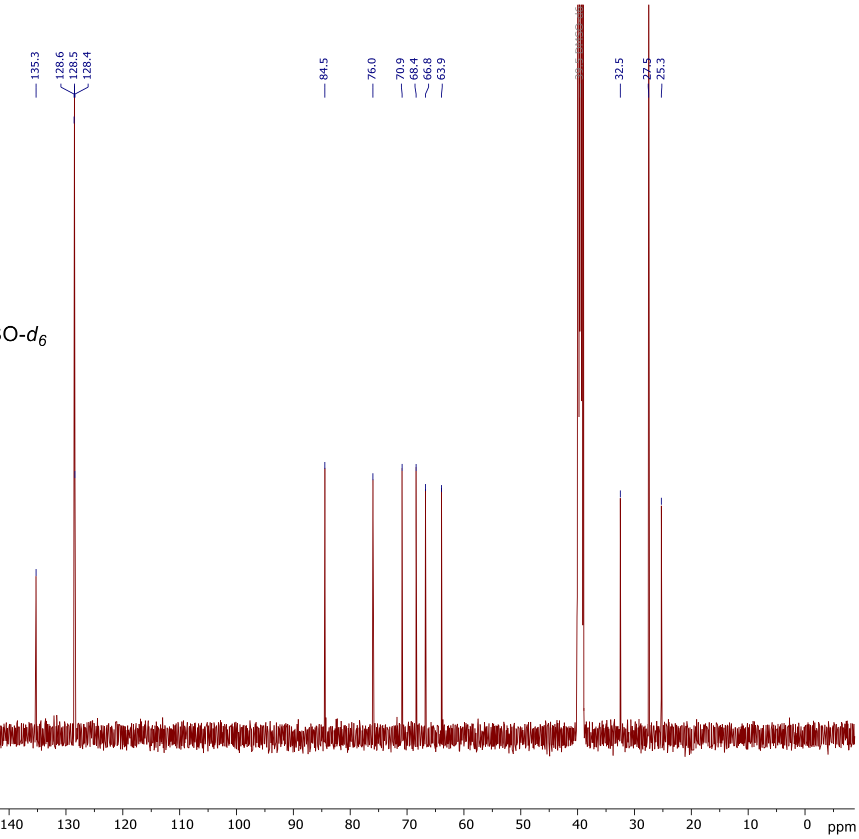

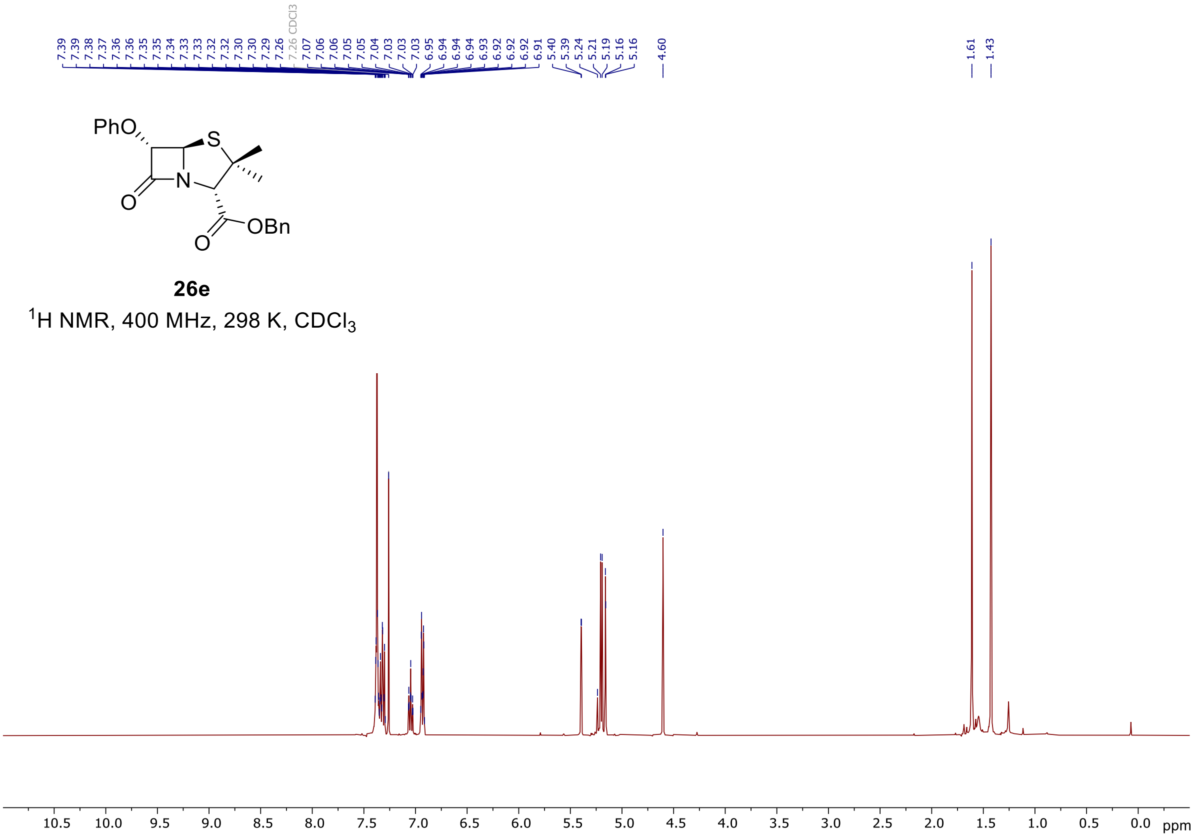

26e

^{1}H NMR, 400 MHz, 298 K, $CDCl_3$

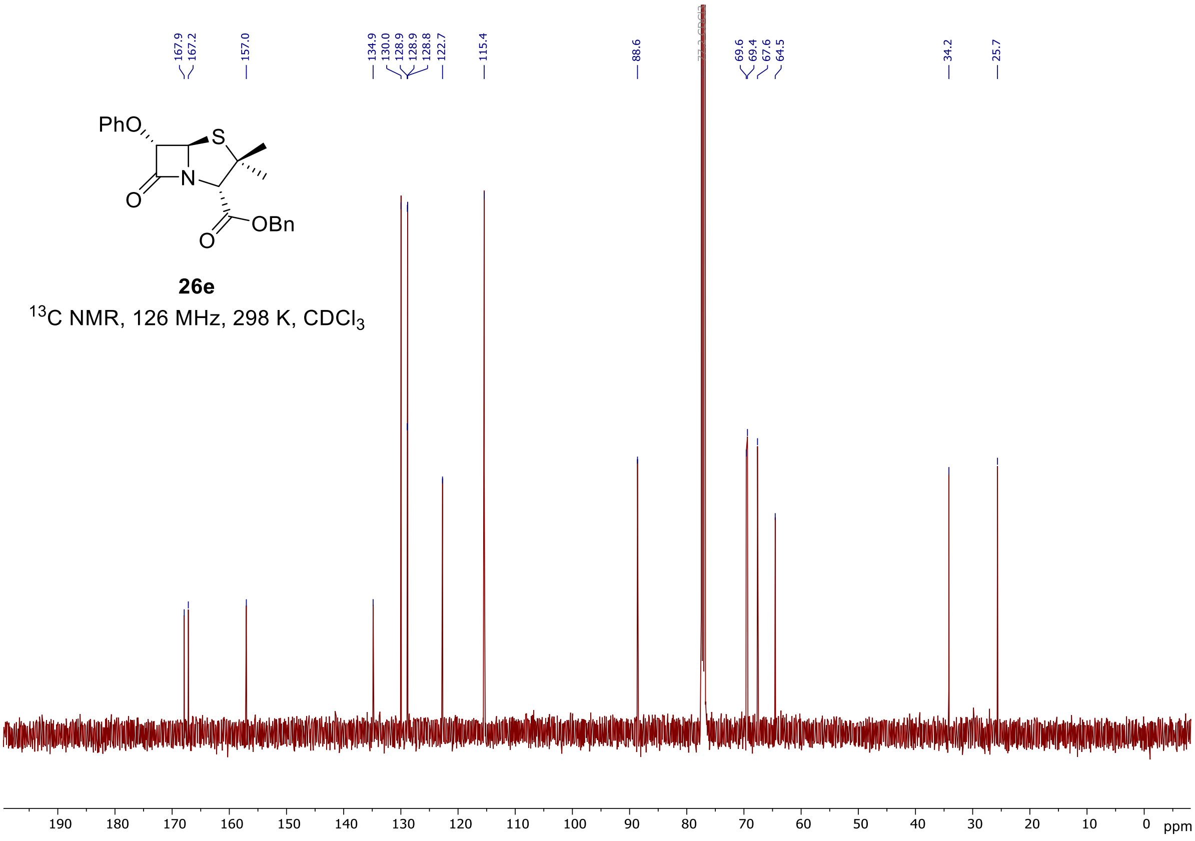

26e

^{13}C NMR, 126 MHz, 298 K, $CDCl_3$

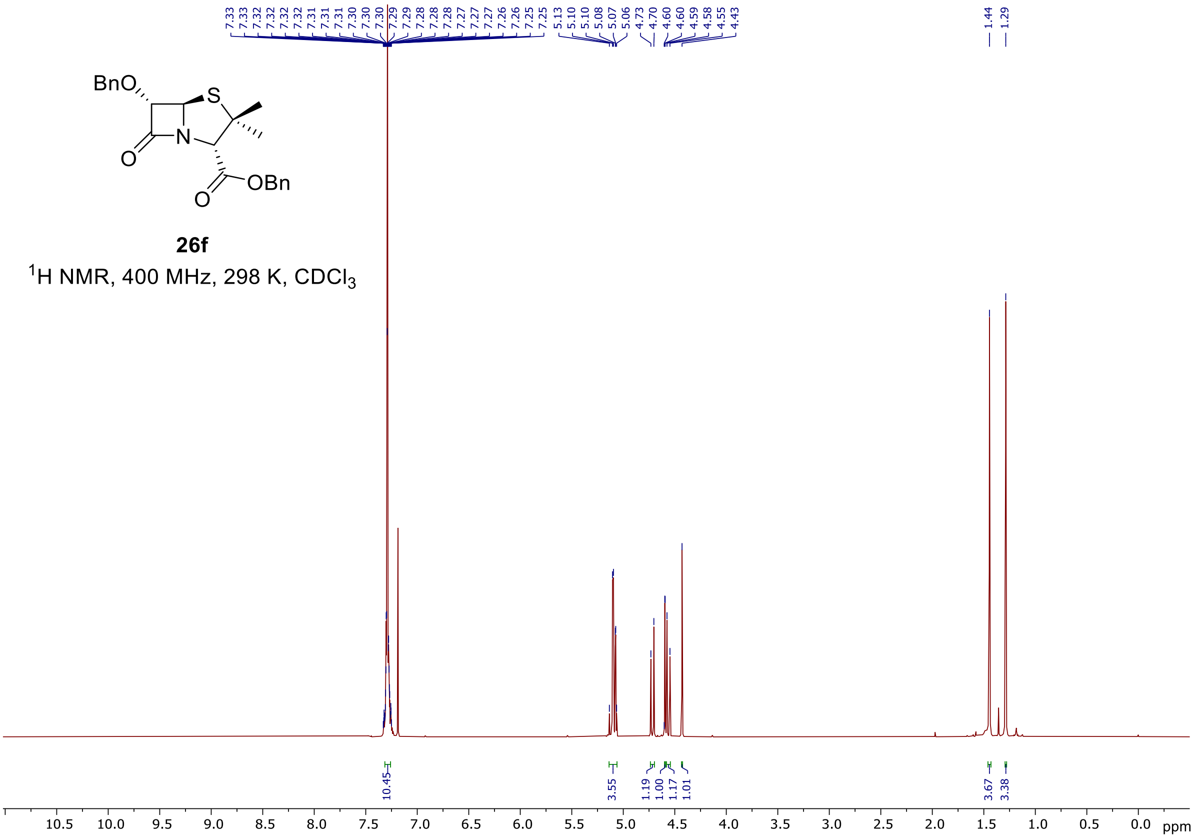

26f

^{1}H NMR, 400 MHz, 298 K, $CDCl_3$

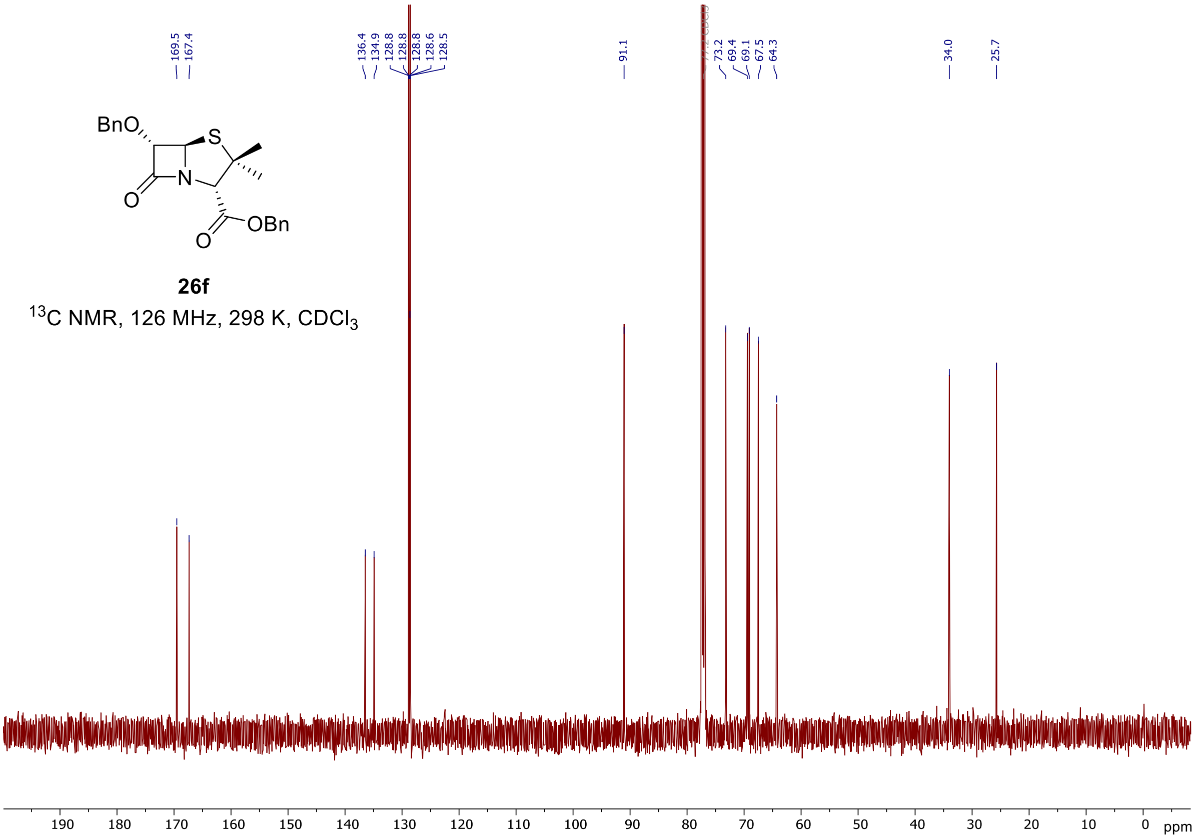

26f

^{13}C NMR, 126 MHz, 298 K, $CDCl_3$

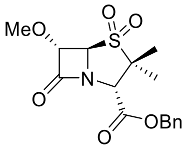

27a

^{1}H NMR, 500 MHz, 298 K, DMSO-d_6

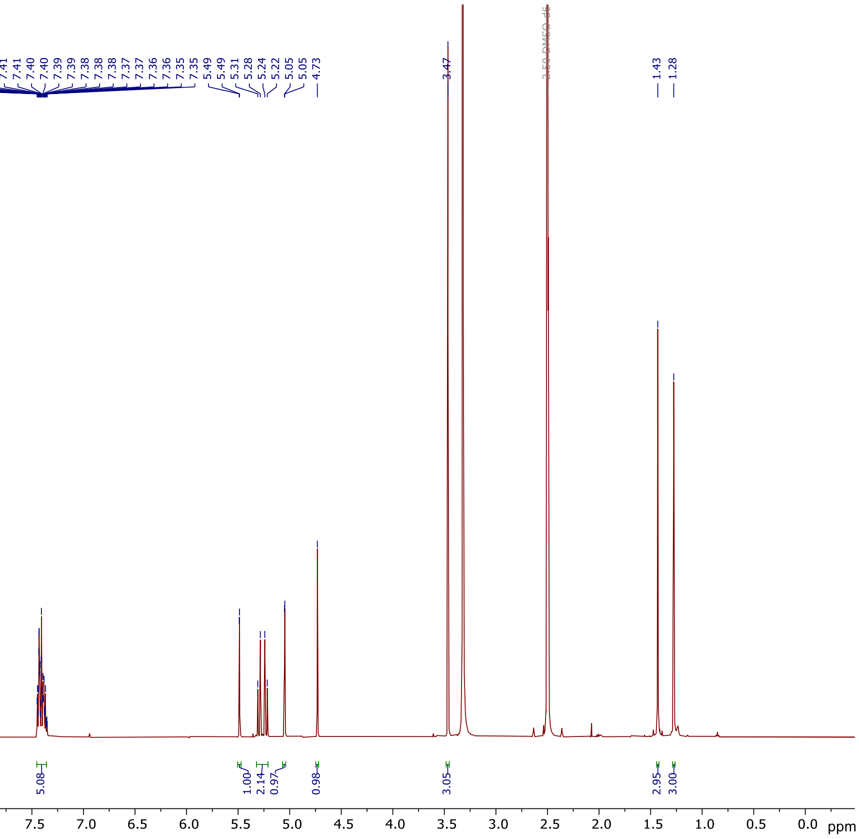

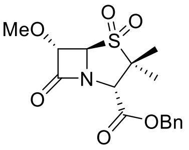

27a

^{13}C NMR, 126 MHz, 298 K, DMSO-d_6

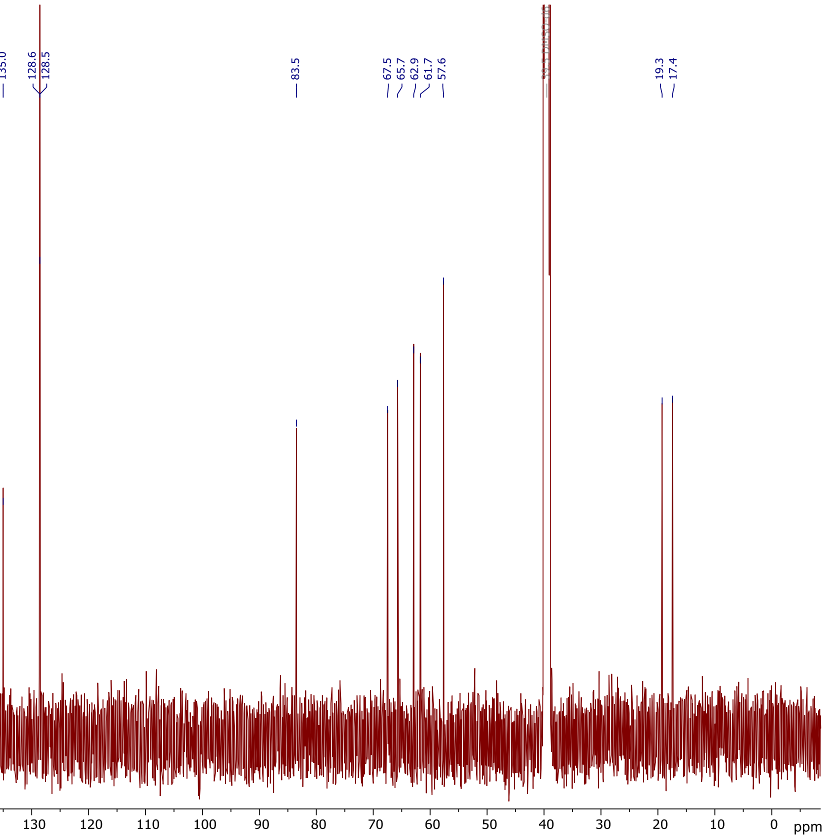

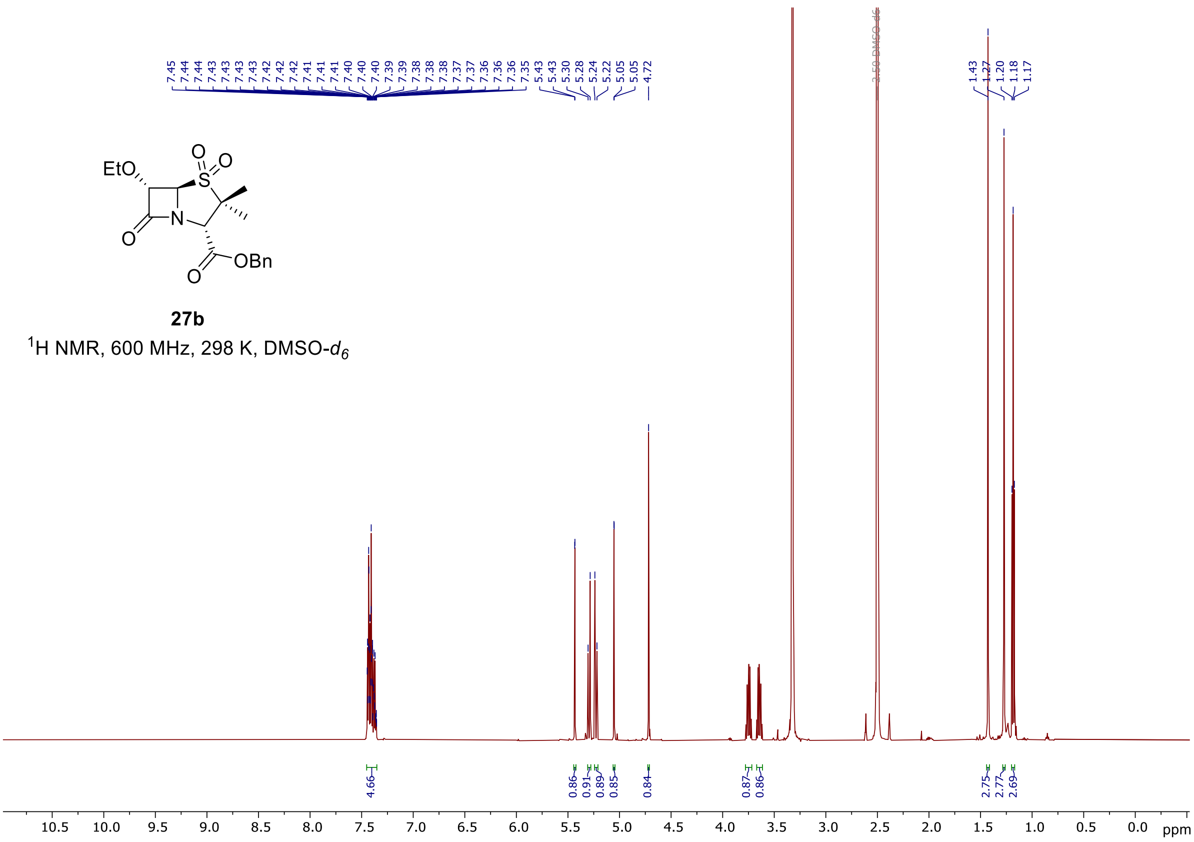
7.45 7.44 7.44 7.43 7.43 7.43 7.43 7.42 7.42 7.42 7.41 7.41 7.41 7.40 7.40 7.40 7.39 7.39 7.38 7.38 7.38 7.37 7.37 7.36 7.36 7.36 7.35
5.43 5.43 5.30 5.28 5.24 5.22 5.05 5.05 4.72
2.50 DMSO-d6
1.43 1.27 1.20 1.18 1.17
EtO
O
O
S
N
O
O
OBn
27b
1H NMR, 600 MHz, 298 K, DMSO-d6
4.66
0.86 0.91 0.89 0.85
0.84
0.87 0.86
2.75 2.77 2.69
10.5 10.0 9.5 9.0 8.5 8.0 7.5 7.0 6.5 6.0 5.5 5.0 4.5 4.0 3.5 3.0 2.5 2.0 1.5 1.0 0.5 0.0 ppm

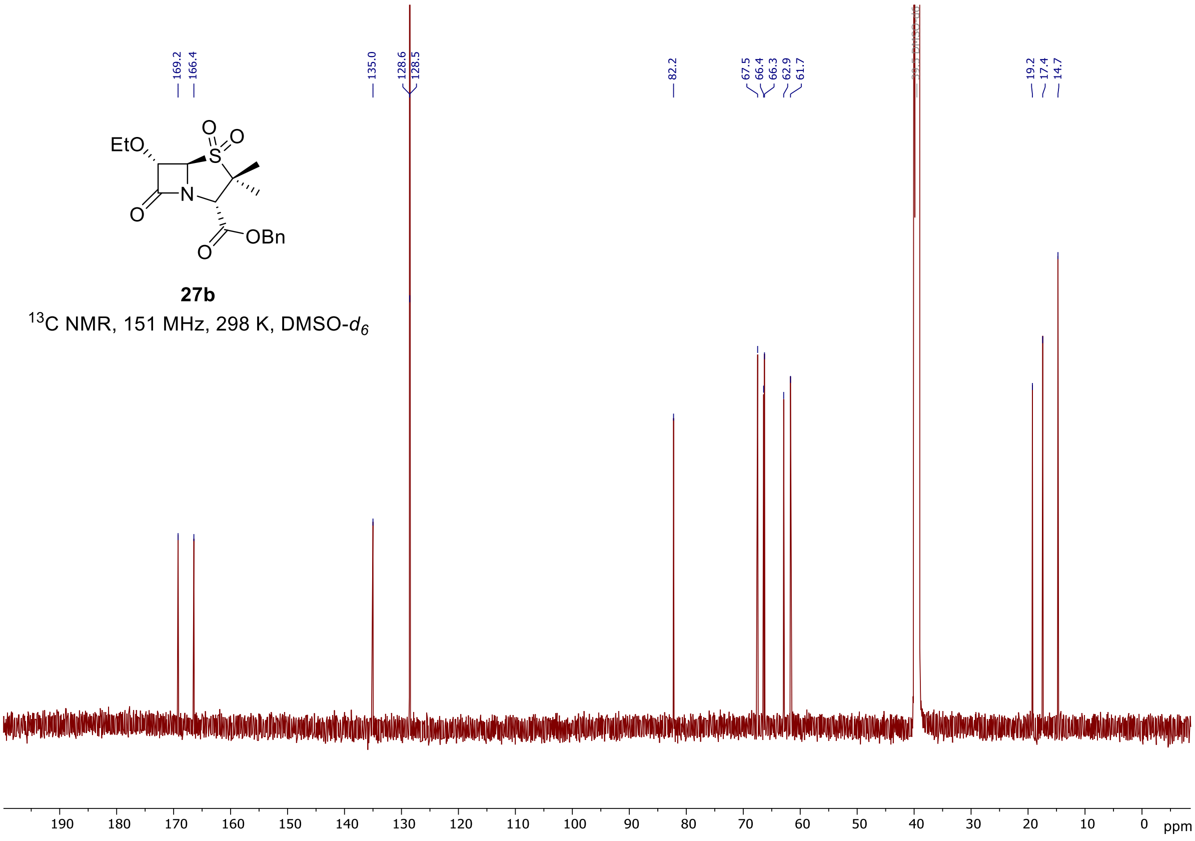
169.2 166.4
135.0
128.6 128.5
82.2
67.5 66.4 66.3 62.9 61.7
19.2 17.4 14.7
EtO
O
O
S
N
O
O
OBn
27b
13C NMR, 151 MHz, 298 K, DMSO-d6
190 180 170 160 150 140 130 120 110 100 90 80 70 60 50 40 30 20 10 0 ppm

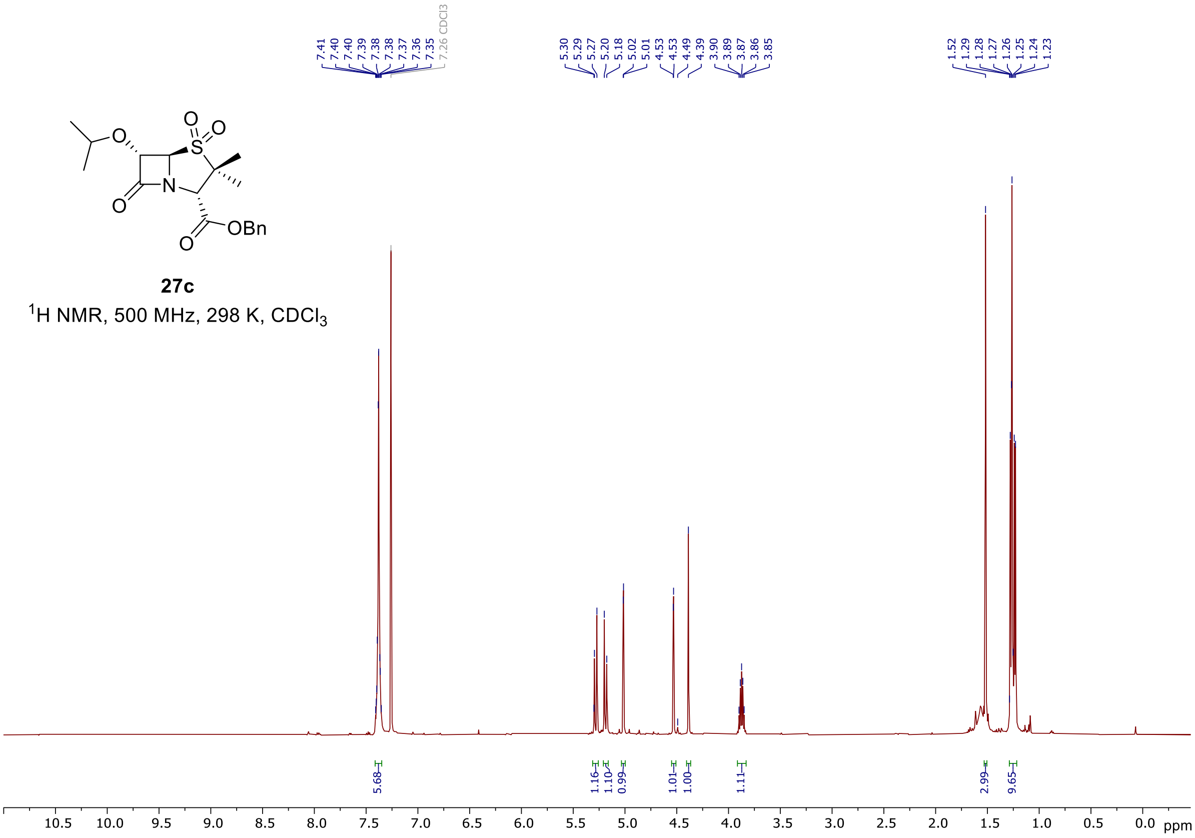

27c

^{1}H NMR, 500 MHz, 298 K, $CDCl_3$

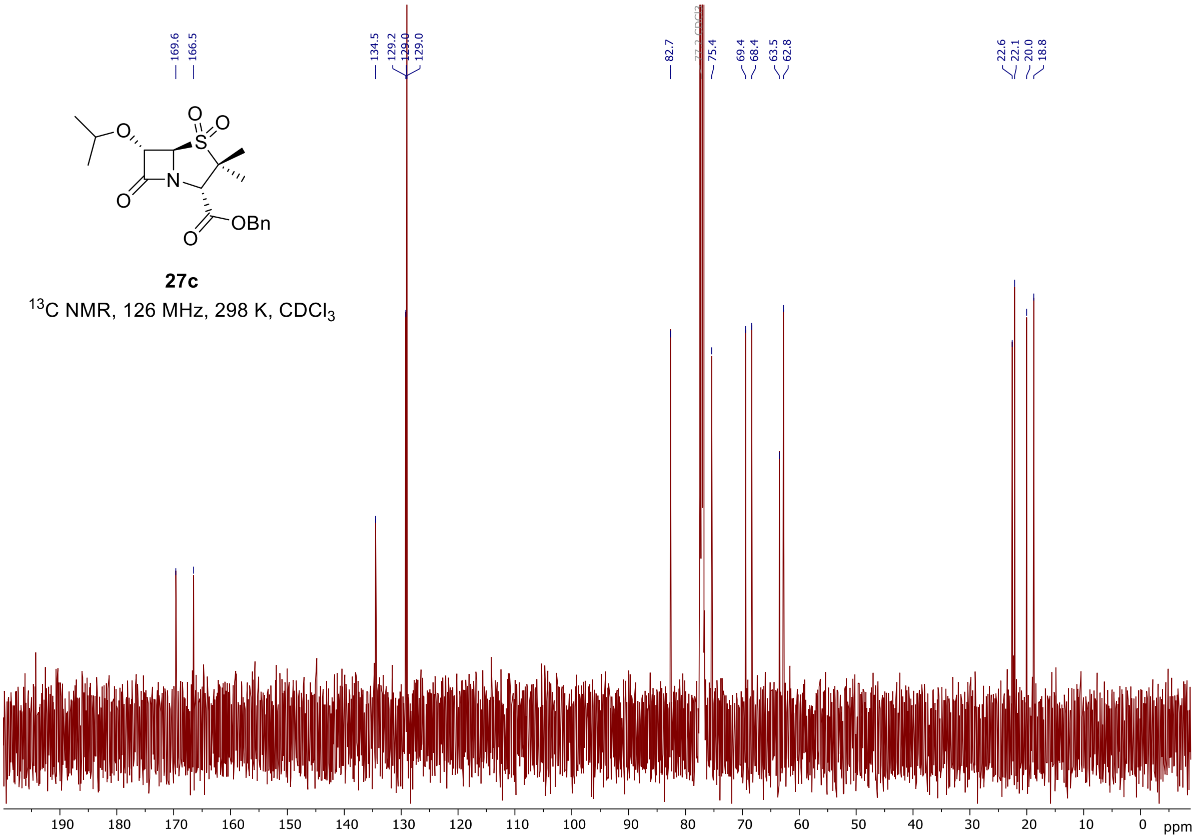

27c

^{13}C NMR, 126 MHz, 298 K, $CDCl_3$

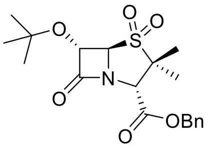

27d

^{1}H NMR, 500 MHz, 298 K, $CDCl_3$

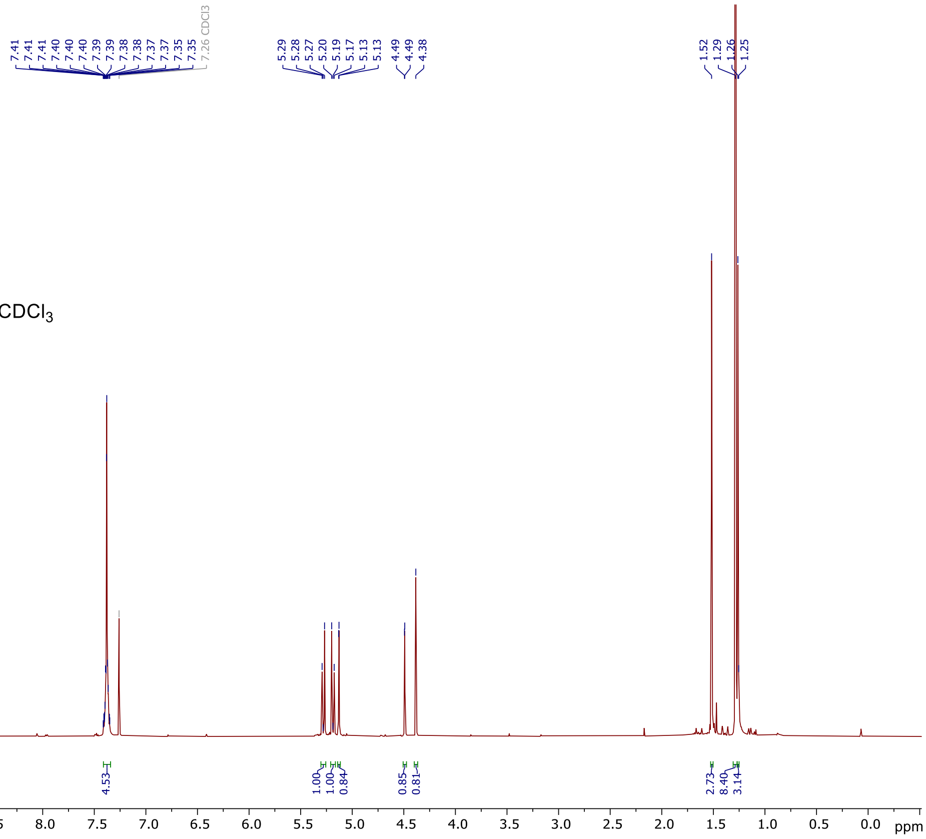

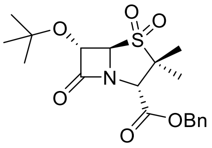

27d

^{13}C NMR, 126 MHz, 298 K, $CDCl_3$

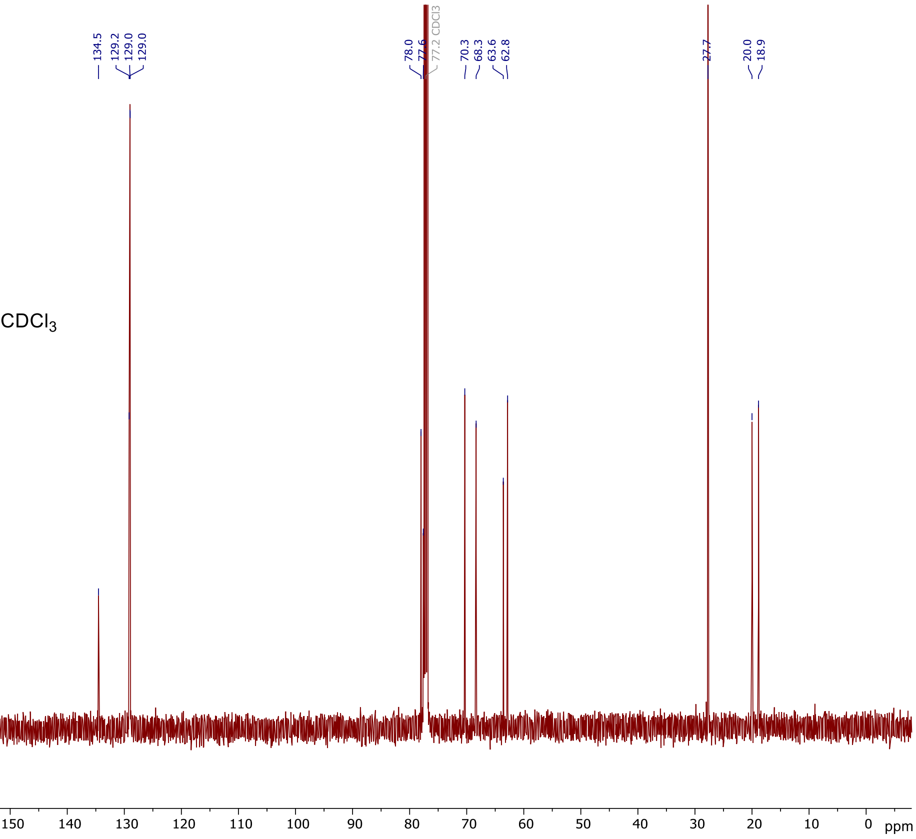

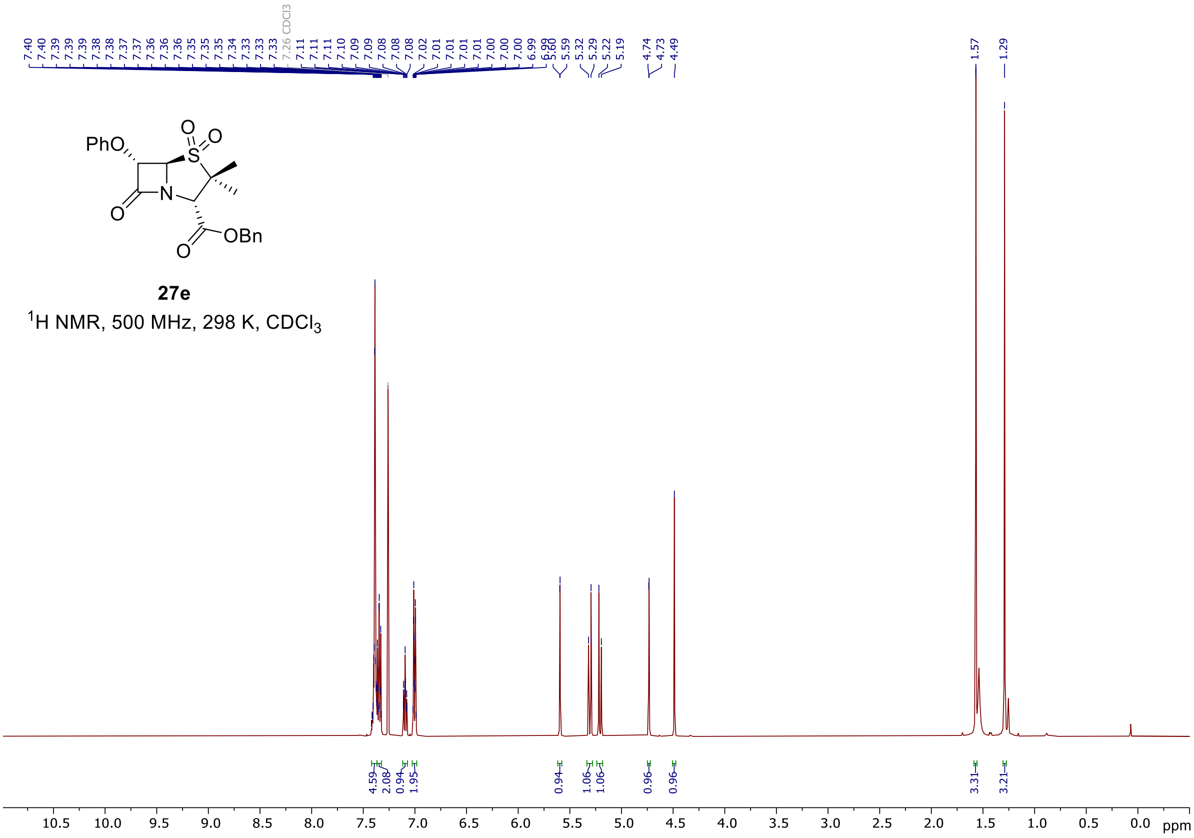

27e

^{1}H NMR, 500 MHz, 298 K, $CDCl_3$

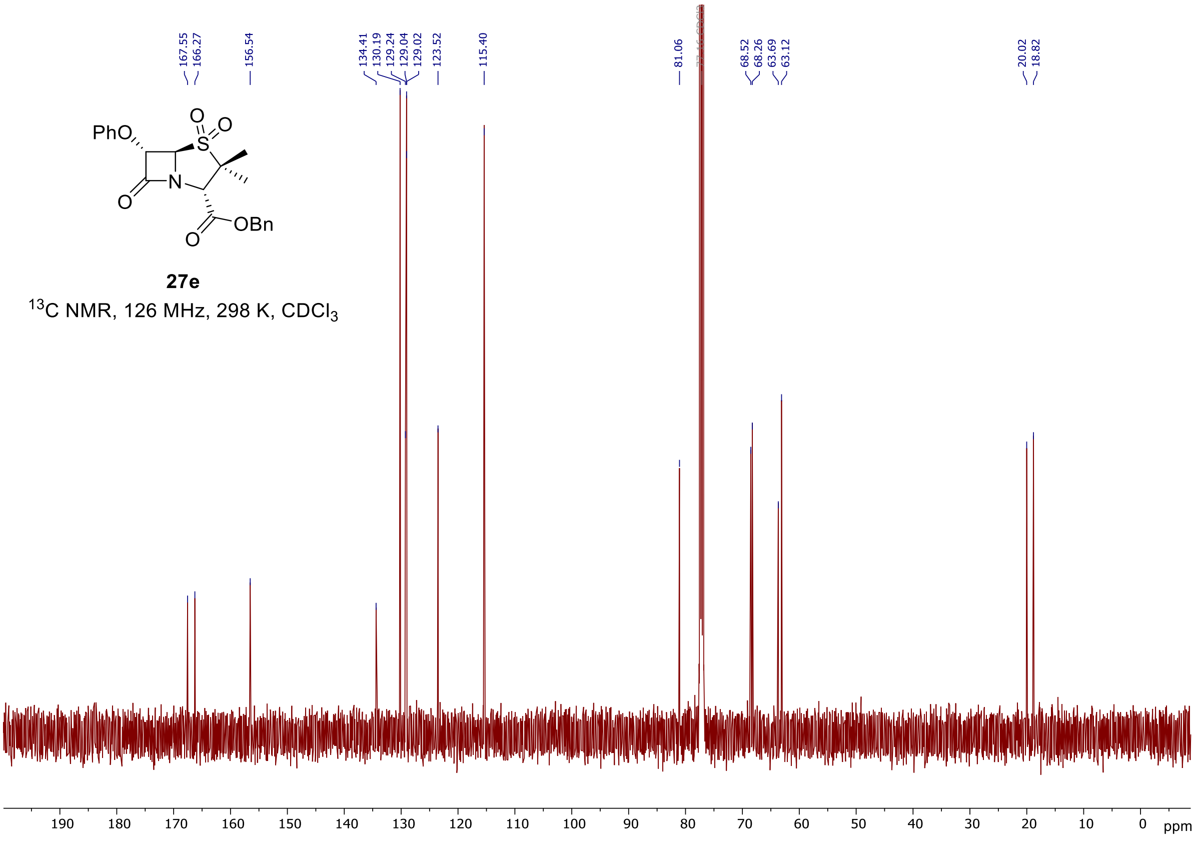

27e

^{13}C NMR, 126 MHz, 298 K, $CDCl_3$

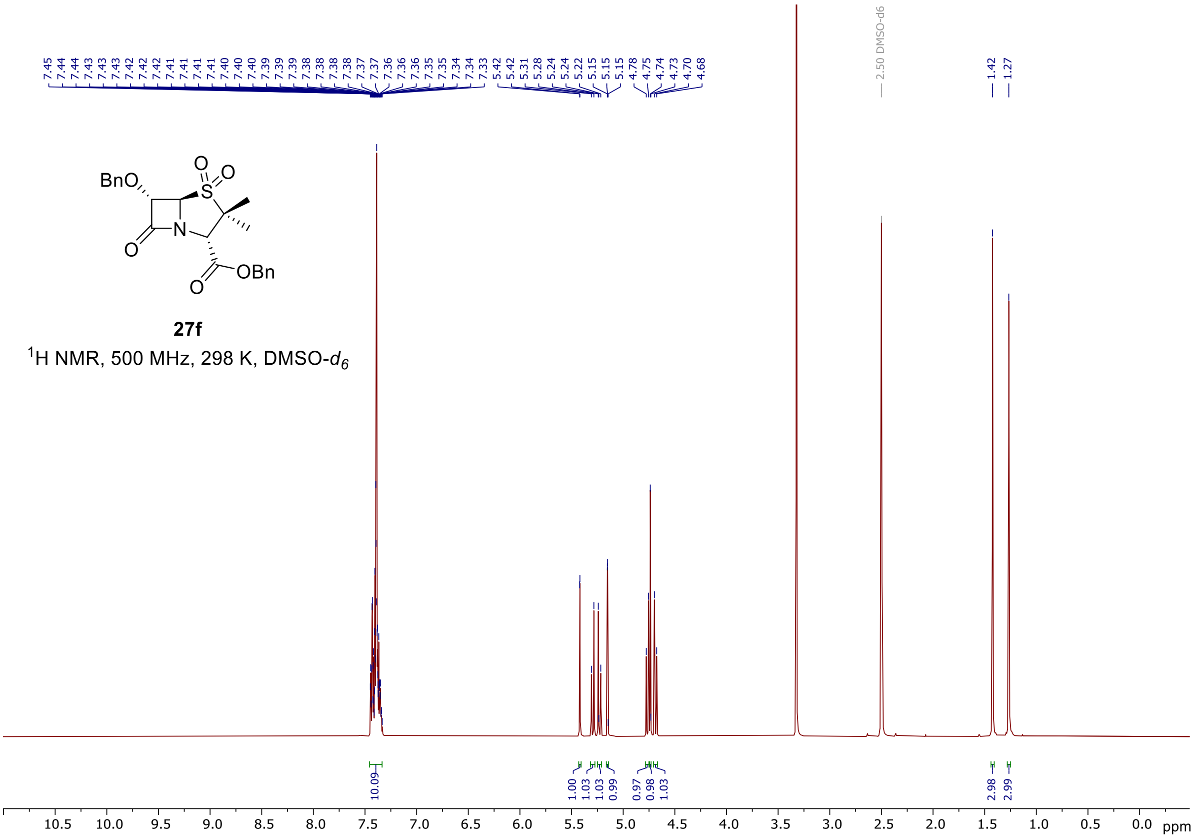

27f

^{1}H NMR, 500 MHz, 298 K, DMSO-d_6

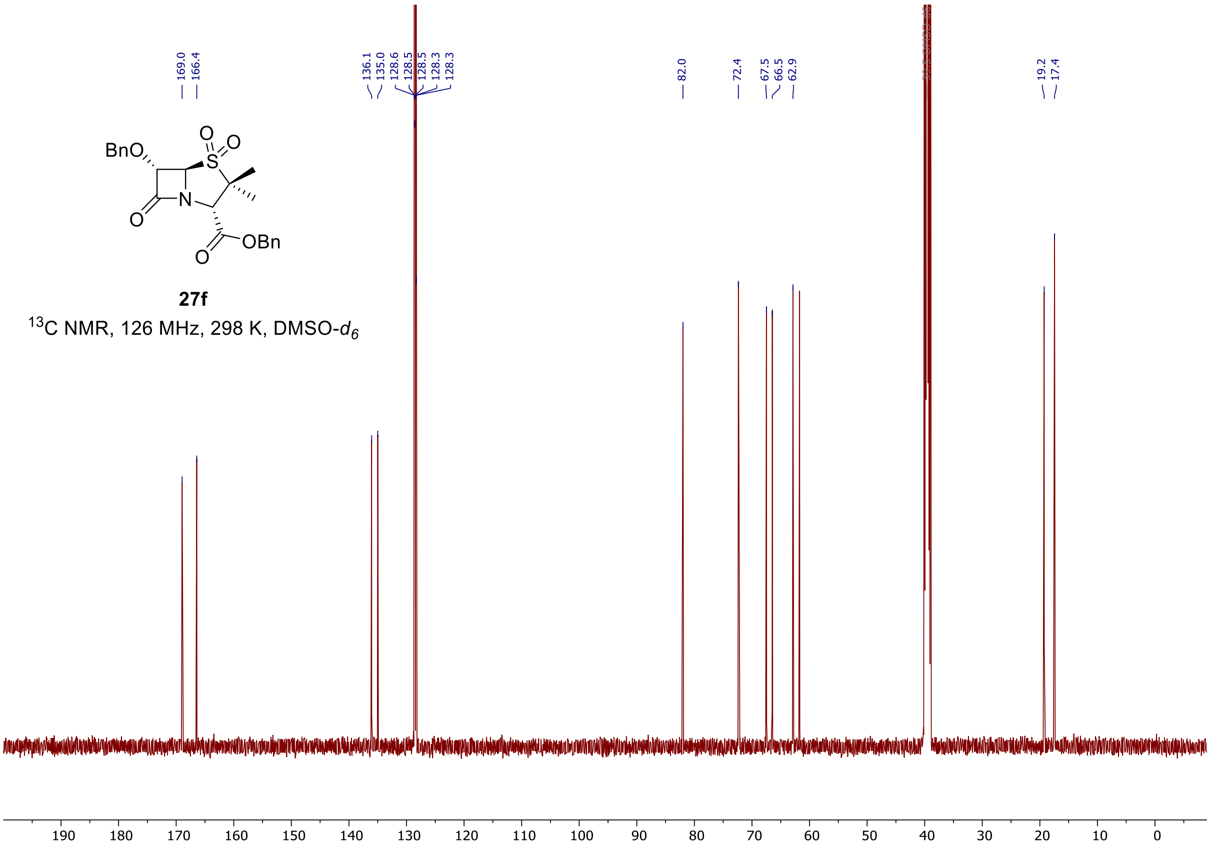

27f

^{13}C NMR, 126 MHz, 298 K, DMSO-d_6
